# Square‐Planar Ruthenium Alkylidyne Complexes Undergo Stepwise Rather Than Concerted [2 + 2] Cycloadditions with Alkynes

**DOI:** 10.1002/anie.202519905

**Published:** 2025-11-10

**Authors:** Mingxu Cui, Markus Leutzsch, Alexander A. Auer, Alois Fürstner

**Affiliations:** ^1^ Max‐Planck‐Institut für Kohlenforschung Mülheim/Ruhr 45470 Germany

**Keywords:** [2 + 2] Cycloaddition, Alkylidyne complexes, Alkyne metathesis, Metallacyclobutadiene, Ruthenium

## Abstract

A new entry into square‐planar, formally d^4^‐configured ruthenium alkylidyne complexes is disclosed, using *p*‐tolyl(trimethylsilyl)diazomethane as a convenient and safe alkylidyne synthon. The method furnished complex **12** supported by an electron‐rich PNP‐pincer ligand, which undergoes remarkably facile [2 + 2] cycloaddition with electron‐rich, electron‐deficient, and strained alkynes; these reactions represent the first examples of metallacyclobutadiene formation by a d^4^‐configured transition metal alkylidyne complex. Strikingly, however, the cycloadditions proceed by a stepwise mechanism, which stands in marked contrast to the concerted pathway entertained by all prototypical d^0^ and d^2^ Schrock‐type alkylidynes, including the molybdenum, tungsten, and rhenium complexes that currently dominate the field of alkyne metathesis. In essence, it is the non‐bonding lone pair forming the largely metal‐centered HOMO of significant d_z2_ character that accounts for this unorthodox behavior. The newly gained insight into this key reactivity determinant also allows the few other known reactions of formally d^4^‐configured alkylidene complexes previously described in the literature to be explained and will empower further explorations of their chemistry.

## Graphical abstract

After decades of research into metal alkylidyne complexes and their extensive use as catalysts for alkyne metathesis, one might think that there is nothing *fundamentally* new to be discovered in this field. The present study into formally d^4^‐configured Ru alkylidynes shows that this notion is incorrect: not only is a novel and convenient entry route disclosed but also an entirely unprecedented stepwise pathway for [2 + 2] cycloaddition reactions is uncovered.

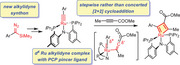

Contemporary alkyne metathesis chemistry is dominated by the use of Schrock‐type d^0^‐alkylidyne (carbyne) complexes **A** of molybdenum and tungsten as the catalysts (Scheme 1).^[^ ^1^, ^2^, ^3^, ^4^, ^5^, ^6^, ^7^, ^8^, ^9^, ^10^, ^11^ ^]^ Although the latest generations combine high activity and ease of handling with an exquisite functional group compatibility,^[^ ^12^, ^13^, ^14^, ^15^, ^16^, ^17^, ^18^, ^19^, ^20^, ^21^, ^22^, ^23^, ^24^, ^25^, ^26^ ^]^ significant limitations still remain that will be difficult to overcome with any such early transition metal complex. High stability toward protic groups in general and the possibility of performing alkyne metathesis reactions in aqueous media are important such desiderata.^[^ ^27^, ^28^ ^]^ To achieve these goals, it might be necessary to search for alternative catalysts based on more “noble” transition metals. Indeed, very encouraging results have recently been disclosed for d^2^‐configured rhenium alkylidynes of type **B** (Scheme 1):^[^ ^29^, ^30^, ^31^, ^32^ ^]^ not only is it noteworthy per se that metal alkylidynes with non‐bonding d electrons show appreciable catalytic activity, but these catalysts were actually found to tolerate unprotected carboxylic acids, which no other currently known type of alkyne metathesis catalyst would be able to withstand. Stoichiometric alkyne metathesis is also documented for d^2^ tungsten alkylidynes;^[^ ^33^ ^]^ moreover, a special type of metalla‐aromatic d^2^ metallapentalynes was shown to undergo stoichiometric [2 + 2] cycloadditions likely driven by strain relief upon relaxation of their extremely distorted endocyclic M≡C bond (M = Ru, Os).^[^ ^34^, ^35^, ^36^ ^]^ In any case, these examples demonstrate that non‐d^0^ metal alkylidynes are able to engage with alkynes in a productive manner.^[^ ^37^, ^38^, ^39^, ^40^, ^41^, ^42^, ^43^, ^44^, ^45^ ^]^

**Scheme 1:**
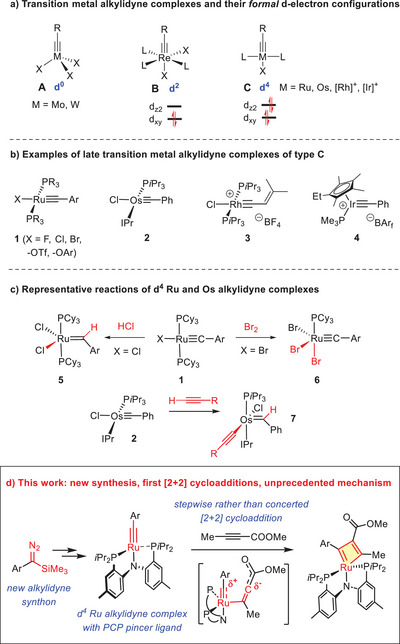
The little‐known chemistry of formally d^4^‐configured late transition metal alkylidyne complexes.

The fundamental question of whether late transition metal alkylidyne complexes with an even higher d‐electron count are candidates for alkyne metathesis catalysts cannot be answered on the basis of the limited knowledge currently available. Only a small number of *formally* d^4^‐configured alkylidyne complexes (counting the alkylidyne as a trianionic ligand)^[^ ^46^ ^]^ of Ru,^[^ ^47^, ^48^, ^49^, ^50^ ^]^ Os,^[^ ^51^ ^]^ Rh,^[^ ^52^, ^53^ ^]^ and Ir^[^ ^54^, ^55^ ^]^ have been described in the literature (Scheme 1b), but surprisingly little is known about their reactivity. For example, Ru alkylidynes **1** are prone to protonation to give Grubbs catalyst **5** (from which they derive); they undergo oxidation with formation of the higher‐valent alkylidyne complexes such as **6**,^[^ ^47^, ^48^, ^49^, ^50^ ^]^ but reactions with alkynes have not been reported. We tested the ruthenium complex **1** ^[^ ^50^ ^]^ ourselves and found it not to react with an assortment of acetylene derivatives. The analogous Os complex **2** was shown to add terminal alkynes across its alkylidyne unit to give the corresponding alkylidene complexes **7**.^[^ ^51^, ^56^ ^]^ There is no information available either if cationic rhodium and iridium alkylidynes such as **3** and **4** are able to activate acetylene derivatives of any sort.^[^ ^52^, ^53^, ^54^, ^55^ ^]^

Because of our longstanding interest in metathesis in general^[^ ^57^, ^58^ ^]^ and alkyne metathesis in particular,^[^ ^2^, ^7^, ^59^, ^60^, ^61^, ^62^, ^63^, ^64^, ^65^, ^66^, ^67^, ^68^, ^69^, ^70^, ^71^, ^72^, ^73^, ^74^ ^]^ we set out to investigate the reactivity of formally d^4^‐configured ruthenium alkylidynes in greater detail. This metal was our first choice in view of the triumph of ruthenium alkylidenes in the mechanistically closely related field of olefin metathesis.^[^ ^75^, ^76^ ^]^ Outlined below, we report a conceptually new method for making formally d^4^‐configured Ru alkylidynes and show for the first time that such complexes are indeed able to engage in [2 + 2] cycloaddition reactions with electron‐rich, electron‐deficient, and strained alkynes. In striking contrast to the literature, however, these reactions follow a stepwise rather than concerted pathway and therefore exemplify a fundamentally new mechanism by which metallacyclobutadienes can be formed.

Inspired by the d^2^‐configured Re alkylidyne system referred to above,^[^ ^29^, ^30^, ^31^ ^]^ it was envisaged that a bulky PNP‐pincer ligand could also predispose a formally d^4^‐configured Ru alkylidyne complex for [2 + 2] cycloaddition. In contrast to the PCy_3_ ligands in **1**, which do not elicit the desired reactivity (see above), we conjectured that the congested ligand environment might disfavor electron back‐donation from the metal center to the π*‐orbital of the incoming alkyne.^[^ ^77^ ^]^ Our attempts at preparing a suitable complex by subjecting known **1** ^[^ ^50^ ^]^ to ligand exchange were unsuccessful. Therefore, we developed an entirely new approach based on the use of α‐silylated diazomethane derivatives **D** as stable, safe, and storable alkylidyne synthon (Scheme 2). On reaction with a suitable metal precursor **E** comprising a halide ligand, **D** was expected to furnish a silylated metal carbene **F** in the first place, which is set up for subsequent elimination of TMSCl to form the targeted M≡C triple bond of complex **G**.

**Scheme 2:**
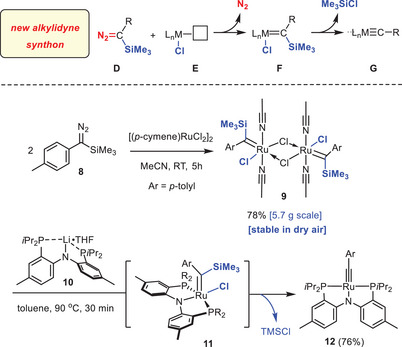
Top: Conceptually new approach to metal alkylidyne complexes using a silylated diazo derivative as alkylidyne synthon. Bottom: application to the synthesis of the formally d^4^‐configured Ru alkylidyne **12** supported by a PNP pincer ligand.

Specifically, when [(*p*‐cymene]RuCl_2_]_2_ was reacted with *p*‐tolyl(trimethylsilyl)diazomethane (**8**)^[^ ^78^, ^79^ ^]^ in MeCN, a green crystalline solid material precipitated from the mixture, which was collected in good yield (78%, 5.7 g scale). X‐ray diffraction analysis showed this product to be the chloride‐bridged dimeric complex **9** with an intact trimethylsilyl group on each of the carbene ligands (Figure 1).^[^ ^80^ ^]^ This complex is bench stable when kept dry and can therefore be easily weighed and handled in air. It is only sparingly soluble in [D_3_]‐MeCN but decomposes instantly in other solvents ([D_8_]‐THF, CD_2_Cl_2_) that might be able to release the stabilizing acetonitrile ligands.

**Figure 1:**
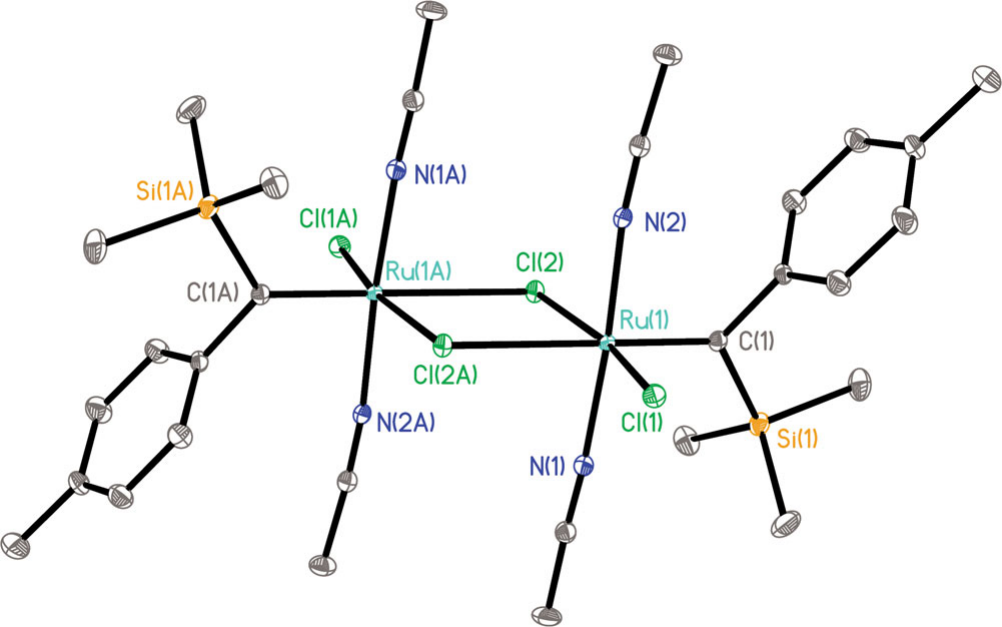
Structure of the chloride‐bridged dimeric ruthenium carbene complex **9** in the solid state (thermal ellipsoids at the 40% probability level; H‐atoms omitted for clarity). Selected bond lengths (Å) and angles (°): Ru(1)─C(1) 1.870(2), Si(1)─C(1) 1.902(3), Ru(1)─N(1) 2.017(2), Ru(1)─N(2) 2.012(2), Ru(1)─Cl(1) 2.3662(7), Ru(1)─Cl(2) 2.3853(7), Ru(1)─Cl(2A) 2.6587(6), Si(1)─C(1)─Ru(1)─Cl(1)─78.47(17).

In contrast to our original expectations, no spontaneous evolution of the silylated carbene into a ruthenium alkylidyne has taken place. This resilience is attributed to the missing interaction between the chloride and the TMS substituent (note that the pertinent dihedral angle Si(1)─C(1)─Ru(1)─Cl(1) is −78.47(17)°). Importantly, however, addition of the lithium salt **10** ^[^ ^81^ ^]^ to a suspension of **9** in toluene led to the formation of the targeted formally d^4^‐configured ruthenium alkylidyne complex **12** by elimination of TMSCl, most likely by passing through **11** as reactive intermediate.^[^ ^82^ ^]^

Complex **12** was isolated in good yield as a dark green solid material. It exhibits a diagnostic resonance of the alkylidyne C‐atom at δ_C_ (C_6_D_6_) = 262.9 ppm (t, *J* = 13.7 Hz), which is notably deshielded relative to that of the chloride‐containing alkylidyne **1** (δ_C _= 237.6 ppm);^[^ ^49^ ^]^ the ^31^P NMR spectrum shows a sharp singlet at δ_P _= 68.06 ppm. In the solid state, the complex adopts a distorted square‐planar geometry (Figure 2).^[^ ^80^ ^]^ With 1.7341(18) Å, the Ru─C distance falls into the range of known Ru≡C triple bonds;^[^ ^47^, ^48^, ^49^, ^50^, ^83^ ^]^ the alkylidyne entity notably deviates from linearity (Ru(1)─C(27)─C(28) 162.63°), as does the N(1)─Ru(1)─C(27) unit (159.96°). Overall, the structure of **12** in the solid state is similar to that of isoelectronic d^4^‐configured Ru(PNP) nitride complexes.^[^ ^84^, ^85^ ^]^

**Figure 2:**
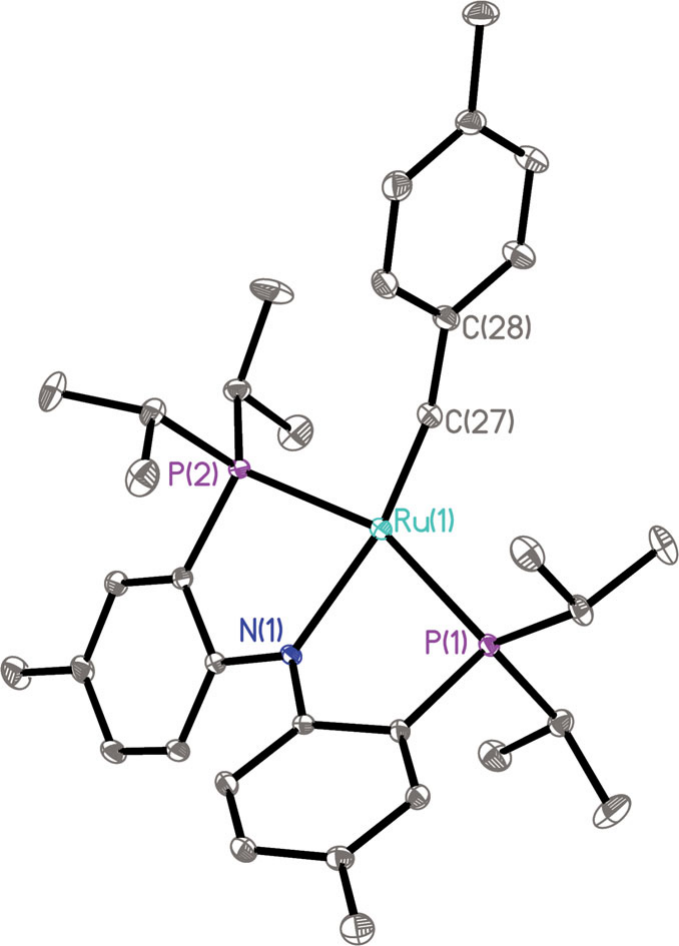
Structure of the ruthenium alkylidyne complex **12** in the solid state (thermal ellipsoids at the 40% probability level; H‐atoms omitted for clarity). Selected bond lengths (Å) and angles (°): Ru(1)─C(27) 1.7341(18), Ru(1)─N(1) 2.1184(14), Ru(1)─P(1) 2.3509(5), Ru(1)─C(27)─C(28) 162.63(15), N(1)─Ru(1)─C(27) 159.95(7).

The new ruthenium alkylidyne **12** did not react with 3‐hexyne, tolane, or 1‐phenylpropyne in toluene even at reflux temperature, where decomposition takes place with time. Phenylacetylene was slowly dimerized on treatment with catalytic amounts of **12** at room temperature, but this reactivity was not investigated any further.^[^ ^86^ ^]^ Gratifyingly, however, a very rapid reaction was observed with 3‐(phenylethynyl)oxazolidin‐2‐one (**13**) in toluene, furnishing the ruthenacyclobutadiene complex **14** exclusively (Scheme 3). The metallacyclic core is manifested in the characteristic deshielded triplets at δ_C_ = 213.7 (*J* = 11.1 Hz) and 205.9 ppm (*J* = 10.5 Hz) for the two inequivalent C_α_ atoms, as well as a less deshielded signal assigned to C_β_ (δ_C_ = 150.8 ppm, t, *J*_C,P _= 5.3 Hz);^[^ ^87^ ^]^ the NOESY data allow the substituents on the metallacyclic ring to be precisely located. The ^31^P NMR spectrum shows a sharp singlet at δ_P _= 47.0 ppm, indicating that the complex is *C*_S_‐symmetric on the NMR time scale at room temperature.

**Scheme 3:**
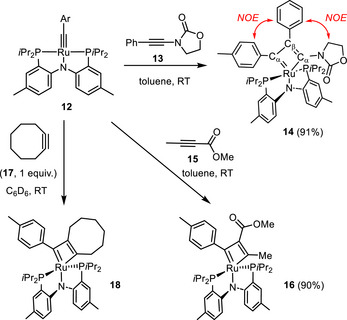
[2 + 2] Cycloaddition reactions of the ruthenium alkylidyne complex **12** with an electron‐rich, an electron‐deficient, and a strained alkyne.

Complex **12** reacts equally well with electron‐deficient methyl 2‐butynoate (**15**) to give the ruthenacyclobutadiene product **16**, which shows similar spectroscopic fingerprints (see the Supporting Information). Dark‐red single crystals of **16** suitable for X‐ray diffraction analysis could be grown, which confirmed the constitution deduced from the NMR data (Figure 3).^[^ ^80^ ^]^ The complex adopts a distorted square‐pyramidal geometry if the η^2^‐C_3_R_3_ unit is viewed as a bidentate ligand. The central metallacyclic array is planar, the C(1)─Ru(1)─C(3) angle is very acute, and the Ru(1)─C(1) and Ru(1)─C(3) bond lengths are notably different (2.001(2) Å and 1.924(3) Å, respectively).^[^ ^88^ ^]^ It is of note that the polar substituents in the metallacyclobutadienes **14** and **16** derived from the electron‐rich ynamide and the electron‐deficient ynoate reside at the α‐C‐atom and β‐C‐atom, respectively. Likewise, alkylidyne **12** reacts within seconds with cyclooctyne (**17**) to give the corresponding ruthenacyclobutadiene **18** in quantitative NMR yield. The three virtual triplets at δ_C_ = 253.0 (t, *J* = 10.6 Hz), 214.2 (t, *J* = 10.5 Hz), and 170.0 (t, *J* = 5.5 Hz) ppm, corresponding to the three C‐atoms of the metallacyclic ring, together with the sharp singlet at δ_P_ = 42.9 ppm, are diagnostic; the HR‐MS data support the assignment.

**Figure 3:**
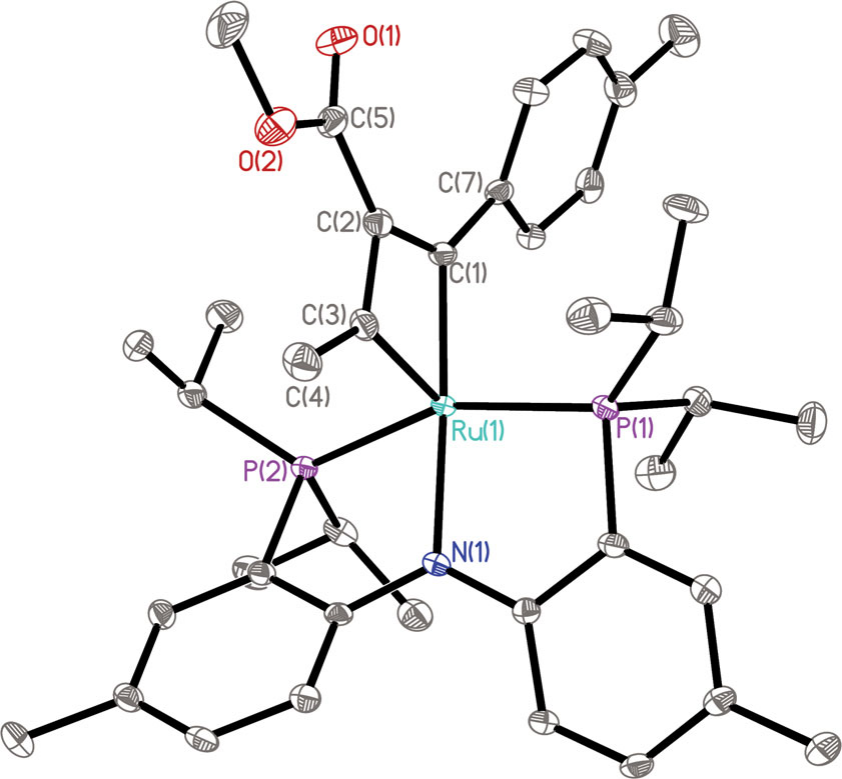
Structure of the ruthenacyclobutadiene complex **16** in the solid state (thermal ellipsoids at the 40% probability level; H‐atoms omitted for clarity). Selected bond lengths (Å) and angles (°): Ru(1)─C(1) 2.001(2), Ru(1)─C(3) 1.924(3), Ru(1)─C(2) 2.583(3), Ru(1)─N(1) 2.146(2), C(1)─C(2) 1.434(3), C(2)─C(3) 1.419(4); C(3)─Ru(1)─C(1) 66.25(11), C(3)─C(2)─C(1) 97.5(2), C(2)─C(1)─Ru(1) 96.10(17), C(2)─C(3)─Ru(1) 100.09(17), C(1)─Ru(1)─N(1) 170.01(9), C(3)─Ru(1)─N(1) 104.24(10), Ru(1)─C(3)─C(1)─C(2) 178.8(3).

When a slight excess of cyclooctyne (1.5 equiv.) was added to the solution of metallacyclobutadiene **18**, two distinct adducts, **19a** and **19b**, were formed as indicated by the additional signals of cyclooctyne ligated to the metal center in the ^13^C NMR spectrum, as well as by HR‐MS data corresponding to [**11 **+ cyclooctyne]^+^ (see Figures S7–S9). **19a** and **19b** become the only detectable species when the excess of cyclooctyne is increased to ≥10 equiv.

Particularly informative was the ^1^H‐^1^H EXSY NMR spectrum of a solution containing a mixture of **18**, **19a**, and **19b**, which proved that these complexes are in exchange with each other (see Figure S10); this observation, in turn, implies that cyclooctyne coordination must be reversible. When the analogous ^31^P‐^31^P EXSY NMR spectrum was recorded with a mixing time of 300 ms, cross peaks of all three species in solution were detected (Scheme 4, bottom right). Upon reducing the mixing time to 30 ms, however, only the exchange between **18**/**19a** and **18**/**19b** could be observed (Scheme 4, bottom left), indicating that no direct interconversion between the two cyclooctyne adducts **19a** and **19b** themselves takes place. Rather, their interconversion evidently occurs by cyclooctyne dissociation and fast tautomerization at the stage of the five‐coordinate metallacyclobutadiene intermediate **18,** which likely proceeds via Berry rotation in analogy to what has been observed with other metallacyclobutadienes rigidified by a chelate ligand framework,^[^ ^89^ ^]^ followed by re‐coordination of the alkyne. Excess cyclooctyne obviously increases the overall barrier to the extent that both discrete tautomers become observable as the corresponding adducts **19a** and **19b**. Despite the high ring strain of cyclooctyne, ring‐opening alkyne metathesis polymerization did not happen.^[^ ^90^, ^91^, ^92^ ^]^

**Scheme 4:**
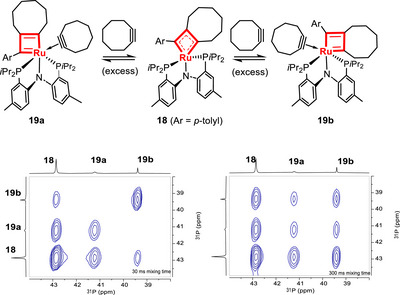
^31^P‐^31^P EXSY spectra of a mixture comprising the three ruthenacyclobutadiene complexes **18**, **19a**, and **19b** recorded at varying mixing times (bottom left: 30 ms; right: 300 ms) show that tautomerization mandates cycloalkyne decoordination; the spectra are plotted with the same intensity threshold.

To the best of our knowledge, these reactions of **12** represent the first examples of [2 + 2] cycloadditions of a formally d^4^‐configured metal alkylidyne in general and of a ruthenium alkylidyne of this type in particular. To better understand the mechanism resulting in metallacyclobutadiene formation, DFT calculations were performed at the B3LYP‐D4/def2‐tzvp/CPCM(toluene) level of theory using the ORCA program suite (Figure 4).^[^ ^93^, ^94^, ^95^ ^]^

**Figure 4:**
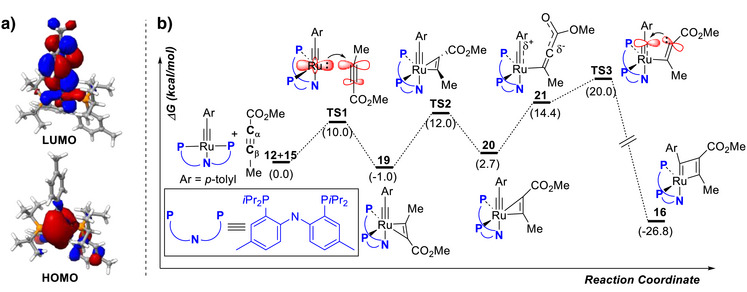
A: HOMO (bottom) and LUMO (top) plots of the Ru alkylidyne complex **12**; B: energy profile of the [2 + 2] cycloaddition reaction of **12** with methyl 2‐butynoate (**15**) [B3LYP‐D4/def2‐tzvp/CPCM(toluene) level of theory].

In a first step, the electronic structure of the four‐coordinate metal alkylidyne complex **12** was analyzed. It adopts a distorted square‐planar geometry because the d_xy_ and d_yz_ orbitals engage in the π‐bonding with the alkylidyne ligand; this leaves d_xz_ and d_z2_ as doubly occupied non‐bonding orbitals, with the latter being the HOMO (for the MO diagram, see Figure S14). As a result, such formally d^4^‐configured 16‐electron transition metal alkylidynes feature frontier orbitals that are reminiscent of those of square‐planar d^8^ complexes.^[^ ^46^ ^]^

The significant d_z2_ character of the HOMO suggests that the Ru center must be fairly electron‐rich, whereas the LUMO is largely located on the alkylidyne fragment. This notion is in line with our experimental observation that two‐electron σ‐donors such as acetonitrile or pyridine do not form adducts with **12**. Since the chelate effect prevents the PNP‐pincer ligand from dissociating (certainly at ambient temperature), coordination of an alkyne to complex **12** should not happen either. If that is the case, however, ordinary [2 + 2] cycloadditions akin to those of d^0^ Mo‐ or W‐alkylidynes commencing with coordination of the substrate to the central atom cannot occur.^[^ ^89^, ^96^, ^97^, ^98^, ^99^, ^100^, ^101^ ^]^

Yet the reaction of **12** with methyl butanoate (**15**), for example, is fast and clean. The computed reaction coordinate shows why that is so (Figure 4): in the first step, the lone pair at the ruthenium center of **12** attacks the vinylogous position of the ynoate (**TS1**). Formally, this transformation can also be seen as an oxidative addition of the alkyne to the Ru center, which donates the non‐bonding electrons in the d_z2_ orbital into the π* orbital of the ligated triple bond. As the Ru–C_α_ interaction in the resulting η^2^‐alkyne‐alkylidyne complex **19** is notably weaker than the Ru─C_β_ bond, rotation is possible via **TS2** to give isomer **20**; upon opening of the weak Ru─C_α_ bond, the ligand gains significant “allenolate” character. This intermediate **21**, in turn, is poised to form the metallacyclobutadiene ring because the nucleophilic carbon center of the allenolate is flanking the electrophilic alkylidyne unit. The computed barriers are low enough to fit the experimental observation that metallacyclobutadiene formation proceeds even at ambient temperature. Importantly, however, the resulting product **16** itself is highly stabilized on thermodynamic grounds, which makes productive cycloreversion and hence catalytic alkyne metathesis with the aid of **12** or closely related complexes unfeasible.

Although the pathway is highly unorthodox by the standards of alkyne metathesis chemistry, it is very plausible according to conventional chemical logic; it is best viewed as a kind of organometallic “Michael addition” reaction triggered by the non‐bonding lone pair of **12**, followed by collapse of the resulting dipolar intermediate. This rationale also allows the reverse substitution pattern of the metallacycle **14** derived from ynamide **13** to be explained: in this case, the C‐atom carrying the oxazolidinone is the more electrophilic site, which is attacked by the lone pair of **12** in the first place; the resulting zwitterionic intermediate will then evolve to the experimentally observed cycloadduct **14** (for details, see the Supporting Information).

In an attempt to gain experimental support for this unprecedented scenario, **12** was reacted with di‐*tert*‐butyl but‐2‐ynedioate (**22**) as a more bulky and even more electron‐deficient substrate in the hope of stabilizing the presumed η^2^‐alkyne–alkylidyne complex to the extent that it can be characterized by spectroscopic means (Scheme 5). Mixing of these reaction partners in [D_8_]‐toluene furnished an unstable dark‐green intermediate **23**, which could nonetheless be characterized at −70 °C by NMR spectroscopy. This compound is distinguished by two resonances at δ_C_ = 127.0 (t, *J* = 7.8 Hz) and 117.0 ppm (t, *J* = 3.2 Hz), which nicely fit to an alkyne tightly bound to a Ru(+2) center able to back‐donate electron density into the antibonding orbitals.^[^ ^102^, ^103^, ^104^, ^105^ ^]^ At the same time, the signal at δ_C_ = 319.2 (t, *J* = 13.0 Hz) shows that the alkylidyne ligand itself is not only intact but also strongly deshielded relative to that of the starting complex **12** (δ_C_ = 262.9 ppm) by a π‐accepting and hence electron‐withdrawing ligand (formally corresponding to an oxidation of the Ru center). Upon warming to ambient temperature, the signals attributed to the ligated triple bond merge into a single resonance, in line with free rotation of the alkyne on the NMR timescale. The dark green intermediate then gradually evolved into the corresponding dark‐red metallacyclobutadiene complex **24**, as evidenced by the characteristic signals at δ_C_ = 252.0 (t, *J* = 10.4) and 238.1 ppm (t, *J* = 9.9 Hz).^[^ ^106^ ^]^

**Scheme 5:**
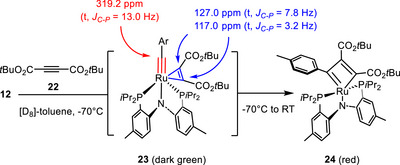
Observation of an η^2^‐alkyne–alkylidyne complex as intermediate en route to the metallacyclobutadiene derived from ruthenium alkylidyne **12** and di‐*tert*‐butyl but‐2‐ynedioate.

In combination with the DFT data, these observations suggest that the d_z2_ orbital as the largely metal‐centered HOMO of **12** is the key reactivity determinant of formally d^4^‐configured metal alkylidyne complexes. Under this premise, other observations concerning their chemical behavior can be readily explained. As mentioned in the introduction, the exceptional ease of protonation of **1** and related species is one of the few well‐documented reactivity patterns (Scheme 1).^[^ ^48^, ^49^, ^50^, ^55^ ^]^ Our results suggest that an acid is unlikely to protonate the alkylidyne right away; rather, the reaction is thought to commence by oxidative addition of, e. g., HCl to the non‐bonding d_z2_ lone pair, followed by 1,2‐hydride shift to furnish Grubbs carbene **5** as the final stable product. It is quite obvious that this pronounced bias, in combination with the new entry route into ruthenium alkylidynes outlined herein, opens a novel and potentially highly enabling gateway to Grubbs‐type catalysts in general; this possibility is currently being intensively studied in our laboratory and will be reported shortly. The reaction of **1** with bromine affords the formally d^2^‐configured Ru alkylidyne **6**, which is an even more stringent illustration, as no migratory insertion does ensue.^[^ ^50^ ^]^ It is safe to assume that the reaction of the Os alkylidyne **2** with a terminal alkyne to give complex **7** follows the same sequence of elementary steps.^[^ ^51^ ^]^

In summary, this investigation proves for the first time that formally d^4^‐configured transition metal alkylidyne complexes can undergo [2 + 2] cycloaddition reactions with alkynes with remarkable ease. Interestingly though, the reactions follow an unprecedented stepwise rather than the canonical concerted pathway; in essence, this switch in mechanism is due to the non‐bonding lone pair forming the largely metal‐centered HOMO of complexes of this type. The tautomerization of the resulting metallacyclobutadiene complexes was also found to have low barriers. The electron‐donating PNP‐pincer ligand favoring this pathway, however, overstabilizes the resulting cycloadducts, such that productive [2 + 2] cycloreversion is prevented and catalytic turnover does not occur. Alkyne metathesis mandates a “flat” reaction‐free energy surface,^[^ ^64^, ^65^, ^107^ ^]^ which the fairly electron‐rich, formally d^4^‐configured metal alkylidyne complexes apparently do not entertain. Attempts at making deliberate use of the fundamentally new reactivity pattern are underway and will be reported in due course.

## Conflict of Interests

A patent application has been filed.

## Supporting information



Supporting information

Supporting information

## Data Availability

The data that support the findings of this study are available in the supplementary material of this article.
