# Supplementary material for: Square‐Planar Ruthenium Alkylidyne Complexes Undergo Stepwise Rather Than Concerted [2 + 2] Cycloadditions with Alkynes
Source: Angew Chem Int Ed Engl. 2025 Nov 10;65(1):e19905. doi: 10.1002/anie.202519905 (PMC12759212; doi:10.1002/anie.202519905)
Supplement: Supplementary file 1 — Supporting information [file ANIE-65-e19905-s002.pdf]

SUPPORTING INFORMATION

Square-Planar Ruthenium Alkylidyne Complexes Undergo Stepwise Rather than Concerted [2+2] Cycloadditions with Alkynes

Mingxu Cui, Markus Leutzsch, Alexander A. Auer, and Alois Fürstner*

Max-Planck-Institut für Kohlenforschung, 45470 Mülheim an der Ruhr, Germany

*E-Mail: fuerstner@kofo.mpg.de

Table of Contents

Crystallographic Information S2

Experimental Procedures S13

General Information S13

Alkylidyne Synthon and PNP Pincer Ligand S14

Synthesis of the Ruthenium Complexes S16

Spectroscopic Observation of the Intermediate 11 .... S19

[2+2] Cycloaddition of the Ruthenium Alkylidyne 12 with Cyclooctyne S22

Tautomerization of the Ruthenacyclobutadienes S27

Spectroscopic Evidence for a Stepwise [2+2] Cycloaddition S29

Computational Study S31

Computational Details S31

Optimized Geometries S35

xyz Coordinates of Computed Structures S36

NMR and IR Spectra S58

References S79

Crystallographic Information

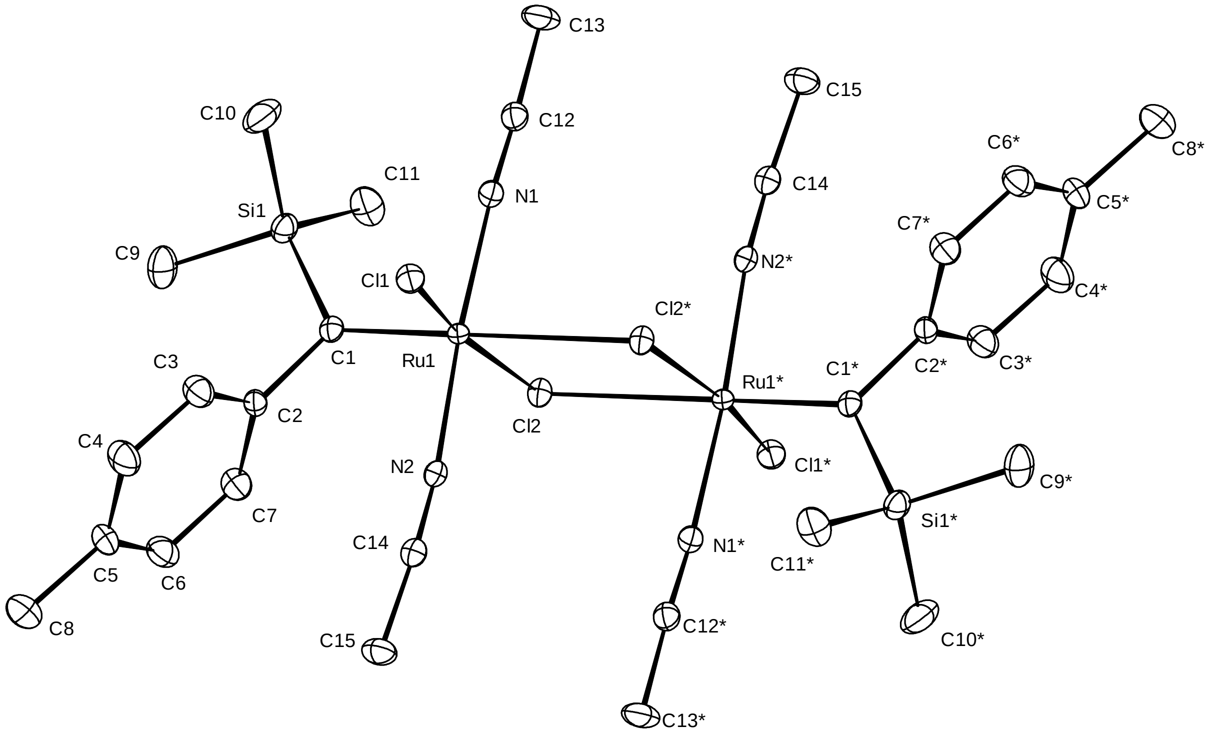

Figure S1. Crystallographic numbering scheme of the Ru carbene dimer complex **9**. The thermal ellipsoid plot is shown at the 50% probability level, H-atoms omitted for clarity.

X-ray Crystal Structure Analysis of Complex 9 [15071]: $C_{30}H_{44}Cl_4N_4Ru_2Si_2$, M_r = 860.81 g · mol^{-1}, green plate, crystal size 0.102 x 0.044 x 0.013 mm^3, monoclinic, space group $P2_1/c$ [14], a = 16.2951(7) Å, b = 8.4477(4) Å, c = 16.0462(7) Å, β = 119.177(2)°, V = 1928.59(15) Å^3, T = 100(2) K, Z = 2, D_{calc} = 1.482 g · cm^3, λ = 0.71073 Å, $\mu(Mo\text{-}K_\alpha)$ = 1.147 mm^{-1}, analytical absorption correction ($T_{\min}$ = 0.91, $T_{\max}$ = 0.99), Bruker-AXS Kappa Mach3 diffractometer with APEX-II detector and IµS micro focus X-ray source, 1.431 < θ < 30.507°, 61950 measured reflections, 5861 independent reflections, 5293 reflections with $I > 2\sigma(I)$, R_{int} = 0.0453, 197 parameters, S = 1.149, residual electron density +0.7 (1.82 Å from H9A) / -1.0 (0.68 Å from Ru1) e · Å^{-3}. The structure was solved by *SHELXT* and refined by full-matrix least-squares (*SHELXL*) against F^2 to R_1 = 0.032 [$I > 2\sigma(I)$], wR_2 = 0.067. **CCDC-2431909**

Table S1. Crystallographic details for complex **9**

Identification code	15071	
Empirical formula	$C_{30} H_{44} Cl_4 N_4 Ru_2 Si_2$	
Color	green	
Formula weight	860.81 g·mol^{-1}	
Temperature	100(2) K	
Wavelength	0.71073 Å	
Crystal system	Monoclinic	
Space group	$P2_1$/c, (no. 14)	
Unit cell dimensions	a = 16.2951(7) Å	α= 90°.
	b = 8.4477(4) Å	β= 119.177(2)°.
	c = 16.0462(7) Å	γ = 90°.
Volume	1928.59(15) Å^3	
Z	2	
Density (calculated)	1.482 Mg·m^{-3}	
Absorption coefficient	1.147 mm^{-1}	
F(000)	872 e	
Crystal size	0.102 x 0.044 x 0.013 mm^3	
θ range for data collection	1.431 to 30.507°.	
Index ranges	-23 ≤ h ≤ 22, -12≤ k ≤ 12, -22 ≤ l ≤ 22	
Reflections collected	61950	
Independent reflections	5861 [R_{int} = 0.0453]	
Reflections with I>2σ(I)	5293	
Completeness to θ = 25.242°	99.2 %	
Absorption correction	Gaussian	
Max. and min. transmission	0.98929 and 0.91456	
Refinement method	Full-matrix least-squares on F^2	
Data / restraints / parameters	5861 / 0 / 197	
Goodness-of-fit on F^2	1.149	
Final R indices [I>2σ(I)]	R_1 = 0.0318	wR^2 = 0.0634
R indices (all data)	R_1 = 0.0395	wR^2 = 0.0668
Extinction coefficient	n/a	
Largest diff. peak and hole	0.729 and -0.987 e·Å^{-3}	

Table S2. Bond lengths [Å] and angles [°] of complex **9**

Ru(1)-C(1)	1.870(2)	Ru(1)-N(2)	2.012(2)
Ru(1)-N(1)	2.017(2)	Ru(1)-Cl(1)	2.3662(7)
Ru(1)-Cl(2)	2.3853(7)	Ru(1)-Cl(2)#1	2.6587(6)
Si(1)-C(11)	1.863(3)	Si(1)-C(9)	1.868(3)
Si(1)-C(10)	1.874(3)	Si(1)-C(1)	1.902(3)
N(1)-C(12)	1.138(4)	N(2)-C(14)	1.138(3)
C(1)-C(2)	1.481(4)	C(2)-C(3)	1.390(4)
C(2)-C(7)	1.398(4)	C(3)-C(4)	1.390(4)
C(4)-C(5)	1.391(4)	C(5)-C(6)	1.384(4)
C(5)-C(8)	1.509(4)	C(6)-C(7)	1.392(4)
C(12)-C(13)	1.453(4)	C(14)-C(15)	1.453(4)
C(1)-Ru(1)-N(2)	90.21(11)	C(1)-Ru(1)-N(1)	93.66(11)
N(2)-Ru(1)-N(1)	176.10(9)	C(1)-Ru(1)-Cl(1)	95.64(8)
N(2)-Ru(1)-Cl(1)	89.63(7)	N(1)-Ru(1)-Cl(1)	90.48(7)
C(1)-Ru(1)-Cl(2)	96.26(8)	N(2)-Ru(1)-Cl(2)	89.34(7)
N(1)-Ru(1)-Cl(2)	89.75(7)	Cl(1)-Ru(1)-Cl(2)	168.07(2)
C(1)-Ru(1)-Cl(2)#1	177.49(9)	N(2)-Ru(1)-Cl(2)#1	90.48(7)
N(1)-Ru(1)-Cl(2)#1	85.64(7)	Cl(1)-Ru(1)-Cl(2)#1	86.78(2)
Cl(2)-Ru(1)-Cl(2)#1	81.34(2)	Ru(1)-Cl(2)-Ru(1)#1	98.66(2)
C(11)-Si(1)-C(9)	106.79(15)	C(11)-Si(1)-C(10)	114.31(17)
C(9)-Si(1)-C(10)	107.16(16)	C(11)-Si(1)-C(1)	112.82(14)
C(9)-Si(1)-C(1)	108.47(14)	C(10)-Si(1)-C(1)	107.03(14)
C(12)-N(1)-Ru(1)	176.1(2)	C(14)-N(2)-Ru(1)	173.0(2)
C(2)-C(1)-Ru(1)	121.46(19)	C(2)-C(1)-Si(1)	113.56(18)
Ru(1)-C(1)-Si(1)	124.85(15)	C(3)-C(2)-C(7)	118.1(3)
C(3)-C(2)-C(1)	120.4(3)	C(7)-C(2)-C(1)	121.4(3)
C(2)-C(3)-C(4)	120.7(3)	C(3)-C(4)-C(5)	121.3(3)
C(6)-C(5)-C(4)	117.9(3)	C(6)-C(5)-C(8)	121.2(3)
C(4)-C(5)-C(8)	120.9(3)	C(5)-C(6)-C(7)	121.4(3)
C(6)-C(7)-C(2)	120.5(3)	N(1)-C(12)-C(13)	177.1(3)
N(2)-C(14)-C(15)	176.1(3)		

Symmetry transformations used to generate equivalent atoms:
#1 -x+1,-y,-z+1

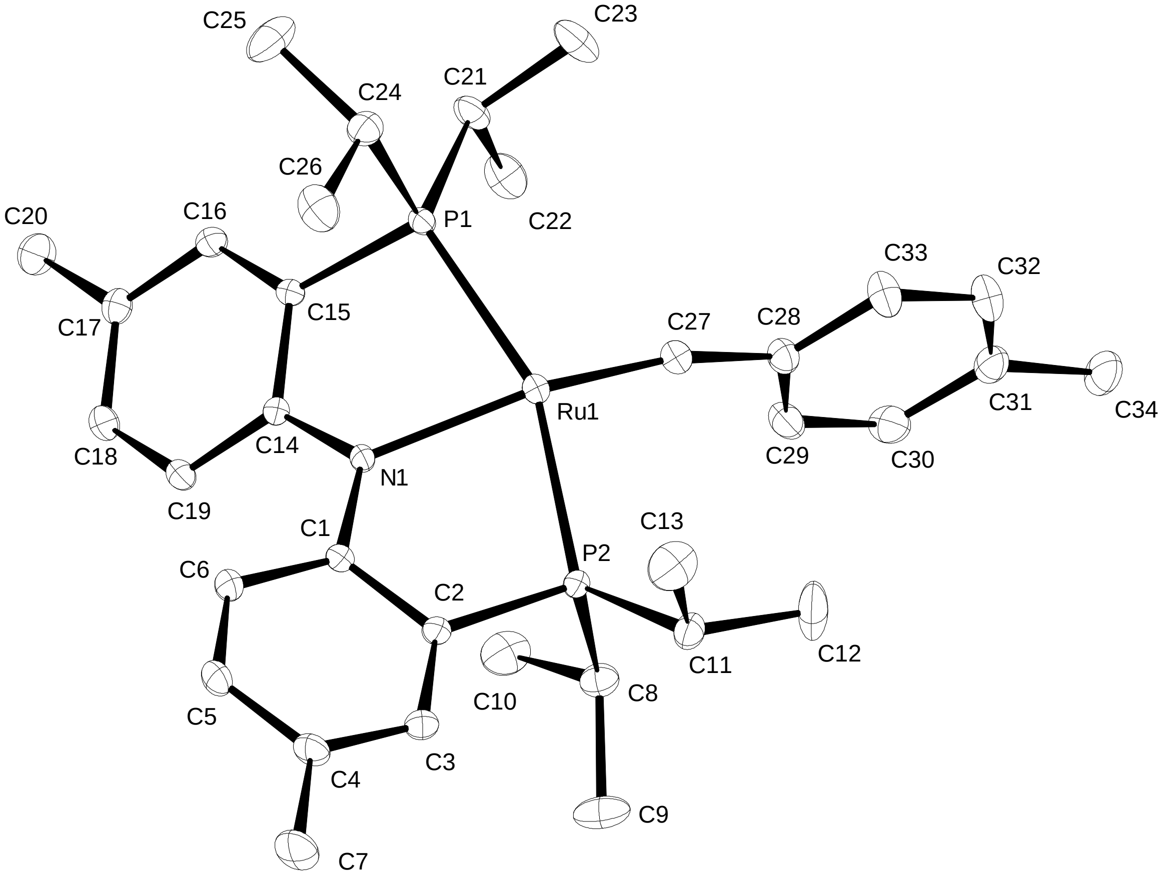

Figure S2. Crystallographic numbering scheme of the Ru alkylidyne complex **12**. The thermal ellipsoid plot is shown at the 50% probability level, H-atoms omitted for clarity.

X-ray Crystal Structure Analysis of Complex 12 [16129]: C_{34} H_{47} N P_2 Ru, M_r = 632.73 g · mol^{-1}, brown plate, crystal size 0.105 x 0.100 x 0.022 mm^3, triclinic, space group $P\bar{1}$ [2], a = 9.3582(2) Å, b = 10.5994(2) Å, c = 17.2462(4) Å, α = 75.1960(10)°, β = 76.6880(10)°, γ = 79.6660(10)°, V = 1596.18(6) Å^3, T = 150(2) K, Z = 2, D_{calc} = 1.316 g · cm^3, λ = 0.71073 Å, μ(Mo-K_α) = 0.614 mm^{-1}, analytical absorption correction ($T_{\min}$ = 0.95, $T_{\max}$ = 0.99), Bruker-AXS D8 VENTURE diffractometer with APEX-II detector and IµS micro focus X-ray source, 2.004 < θ < 31.560°, 180091 measured reflections, 10667 independent reflections, 9407 reflections with $I > 2\sigma(I)$, R_{int} = 0.0773, 354 parameters, S = 1.078, residual electron density +0.9 (0.53 Å from P2) / -0.7 (0.46 Å from Ru1) e · Å^{-3}. The structure was solved by *SHELXT* and refined by full-matrix least-squares (*SHELXL*) against F^2 to R_1 = 0.031 [$I > 2\sigma(I)$], wR_2 = 0.087. **CCDC-2431913**

Table S3. Crystallographic details for complex **12**

Identification code	16129	
Empirical formula	$C_{34} H_{47} N P_2 Ru$	
Color	brown	
Formula weight	632.73 g · mol^{-1}	
Temperature	100(2) K	
Wavelength	0.71073 Å	
Crystal system	TRICLINIC	
Space group	P$\bar{1}$, (no. 2)	
Unit cell dimensions	a = 9.3582(2) Å	α= 75.1960(10)°.
	b = 10.5994(2) Å	β= 76.6880(10)°.
	c = 17.2462(4) Å	γ = 79.6660(10)°.
Volume	1596.18(6) Å^3	
Z	2	
Density (calculated)	1.316 Mg · m^{-3}	
Absorption coefficient	0.614 mm^{-1}	
F(000)	664 e	
Crystal size	0.105 x 0.100 x 0.022 mm^3	
θ range for data collection	2.004 to 31.560°.	
Index ranges	-13 ≤ h ≤ 13, -15≤ k ≤ 15, -25 ≤ l ≤ 25	
Reflections collected	180091	
Independent reflections	10667 [R_{int} = 0.0773]	
Reflections with I>2σ(I)	9407	
Completeness to θ = 25.242°	99.9 %	
Absorption correction	Gaussian	
Max. and min. transmission	0.99 and 0.95	
Refinement method	Full-matrix least-squares on F^2	
Data / restraints / parameters	10667 / 0 / 354	
Goodness-of-fit on F^2	1.078	
Final R indices [I>2σ(I)]	R_1 = 0.0313	wR^2 = 0.0840
R indices (all data)	R_1 = 0.0382	wR^2 = 0.0872
Largest diff. peak and hole	0.9 and -0.7 e · Å^{-3}	

Table S4. Bond lengths [Å] and angles [°] of complex **12**

Ru(1)-P(1)	2.3509(5)	Ru(1)-P(2)	2.3142(4)
Ru(1)-N(1)	2.1184(14)	Ru(1)-C(27)	1.7341(18)
P(1)-C(15)	1.8184(17)	P(1)-C(21)	1.8399(18)
P(1)-C(24)	1.8574(19)	P(2)-C(2)	1.8137(17)
P(2)-C(8)	1.8543(19)	P(2)-C(11)	1.8386(19)
N(1)-C(1)	1.398(2)	N(1)-C(14)	1.396(2)
C(1)-C(2)	1.411(2)	C(1)-C(6)	1.407(2)
C(2)-C(3)	1.395(2)	C(3)-C(4)	1.397(3)
C(4)-C(5)	1.392(3)	C(4)-C(7)	1.508(3)
C(5)-C(6)	1.390(2)	C(8)-C(9)	1.525(3)
C(8)-C(10)	1.533(3)	C(11)-C(12)	1.522(3)
C(11)-C(13)	1.524(3)	C(14)-C(15)	1.414(2)
C(14)-C(19)	1.405(2)	C(15)-C(16)	1.395(2)
C(16)-C(17)	1.399(2)	C(17)-C(18)	1.392(3)
C(17)-C(20)	1.505(2)	C(18)-C(19)	1.391(2)
C(21)-C(22)	1.521(3)	C(21)-C(23)	1.535(3)
C(24)-C(25)	1.530(3)	C(24)-C(26)	1.527(3)
C(27)-C(28)	1.452(2)	C(28)-C(29)	1.403(3)
C(28)-C(33)	1.396(3)	C(29)-C(30)	1.391(3)
C(30)-C(31)	1.397(3)	C(31)-C(32)	1.381(3)
C(31)-C(34)	1.507(3)	C(32)-C(33)	1.393(3)
P(2)-Ru(1)-P(1)	157.987(17)	N(1)-Ru(1)-P(1)	78.36(4)
N(1)-Ru(1)-P(2)	79.63(4)	C(27)-Ru(1)-P(1)	105.10(6)
C(27)-Ru(1)-P(2)	95.44(6)	C(27)-Ru(1)-N(1)	159.95(7)
C(15)-P(1)-Ru(1)	99.87(5)	C(15)-P(1)-C(21)	107.06(8)
C(15)-P(1)-C(24)	104.27(8)	C(21)-P(1)-Ru(1)	120.97(7)
C(21)-P(1)-C(24)	104.07(9)	C(24)-P(1)-Ru(1)	118.76(6)
C(2)-P(2)-Ru(1)	101.09(6)	C(2)-P(2)-C(8)	105.27(8)
C(2)-P(2)-C(11)	105.34(8)	C(8)-P(2)-Ru(1)	114.06(6)
C(11)-P(2)-Ru(1)	124.11(6)	C(11)-P(2)-C(8)	105.09(9)
C(1)-N(1)-Ru(1)	119.31(11)	C(14)-N(1)-Ru(1)	118.62(11)
C(14)-N(1)-C(1)	121.69(14)	N(1)-C(1)-C(2)	118.19(15)
N(1)-C(1)-C(6)	124.15(15)	C(6)-C(1)-C(2)	117.40(15)
C(1)-C(2)-P(2)	114.01(12)	C(3)-C(2)-P(2)	125.19(13)
C(3)-C(2)-C(1)	120.70(16)	C(2)-C(3)-C(4)	121.71(16)
C(3)-C(4)-C(7)	121.38(17)	C(5)-C(4)-C(3)	117.24(16)
C(5)-C(4)-C(7)	121.38(17)	C(6)-C(5)-C(4)	122.16(17)
C(5)-C(6)-C(1)	120.76(16)	C(9)-C(8)-P(2)	114.35(14)
C(9)-C(8)-C(10)	110.39(18)	C(10)-C(8)-P(2)	109.93(13)
C(12)-C(11)-P(2)	110.39(14)	C(12)-C(11)-C(13)	111.29(19)
C(13)-C(11)-P(2)	110.20(13)	N(1)-C(14)-C(15)	117.75(15)

N(1)-C(14)-C(19)	124.61(15)	C(19)-C(14)-C(15)	117.48(15)
C(14)-C(15)-P(1)	113.75(12)	C(16)-C(15)-P(1)	125.60(13)
C(16)-C(15)-C(14)	120.55(15)	C(15)-C(16)-C(17)	121.84(16)
C(16)-C(17)-C(20)	121.74(17)	C(18)-C(17)-C(16)	117.15(16)
C(18)-C(17)-C(20)	121.01(16)	C(19)-C(18)-C(17)	122.17(16)
C(18)-C(19)-C(14)	120.81(16)	C(22)-C(21)-P(1)	109.96(13)
C(22)-C(21)-C(23)	110.82(17)	C(23)-C(21)-P(1)	110.19(13)
C(25)-C(24)-P(1)	113.67(14)	C(26)-C(24)-P(1)	109.62(13)
C(26)-C(24)-C(25)	110.74(18)	C(28)-C(27)-Ru(1)	162.63(15)
C(29)-C(28)-C(27)	119.57(17)	C(33)-C(28)-C(27)	122.25(17)
C(33)-C(28)-C(29)	118.17(17)	C(30)-C(29)-C(28)	120.40(18)
C(29)-C(30)-C(31)	121.04(19)	C(30)-C(31)-C(34)	120.10(19)
C(32)-C(31)-C(30)	118.48(18)	C(32)-C(31)-C(34)	121.42(19)
C(31)-C(32)-C(33)	121.00(19)	C(32)-C(33)-C(28)	120.86(19)

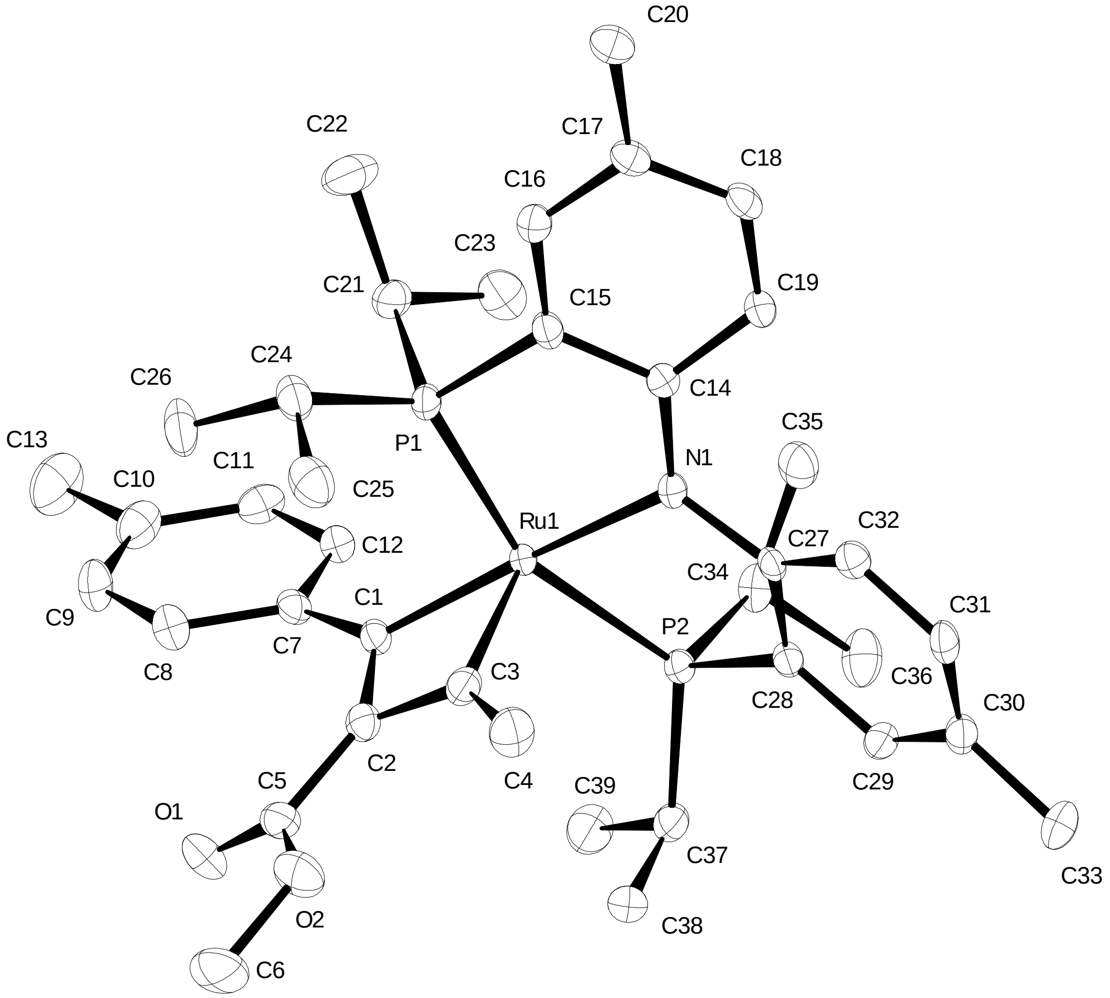

Figure S3. Crystallographic numbering scheme of the ruthenacyclobutadiene complex **16**. The thermal ellipsoid plot is shown at the 50% probability level, H-atoms omitted for clarity

X-ray Crystal Structure Analysis of Complex 16 [15968]: $C_{39} H_{53} N O_2 P_2 Ru$, M_r = 730.83 g · mol^{-1}, black plate, crystal size 0.134 x 0.055 x 0.013 mm^3, monoclinic, space group $P2_1/c$ [14], a = 12.3404(4) Å, b = 9.3880(3) Å, c = 31.5342(10) Å, β = 94.390(2)°, V = 3642.6(2) Å^3, T = 100(2) K, Z = 4, D_{calc} = 1.333 g · cm^3, λ = 0.71073 Å, $\mu(Mo\text{-}K_\alpha)$ = 0.552 mm^{-1}, analytical absorption correction (T_{min} = 0.94, T_{max} = 0.99), Bruker AXS Enraf-Nonius KappaCCD diffractometer with APEX-II detector and IµS micro focus X-ray source, 1.295 < θ < 31.688°, 95671 measured reflections, 12216 independent reflections, 8261 reflections with $I > 2\sigma(I)$, R_{int} = 0.0930, 419 parameters, S = 1.012, residual electron density +0.9 (1.17 Å from Ru1) / -1.2 (0.91 Å from Ru1) e · Å^{-3}. The structure was solved by *SHELXT* and refined by full-matrix least-squares (*SHELXL*) against F^2 to R_1 = 0.049 [$I > 2\sigma(I)$], wR_2 = 0.097. **CCDC-2431910**

Table S5. Crystallographic details for complex **16**

Identification code	15968	
Empirical formula	$C_{39} H_{53} N O_2 P_2 Ru$	
Color	black	
Formula weight	730.83 g · mol^{-1}	
Temperature	100(2) K	
Wavelength	0.71073 Å	
Crystal system	MONOCLINIC	
**Space group**	**$P2_1/c$, (no. 14)**	
Unit cell dimensions	a = 12.3404(4) Å	α= 90°.
	b = 9.3880(3) Å	β= 94.390(2)°.
	c = 31.5342(10) Å	γ = 90°.
Volume	3642.6(2) $Å^3$	
Z	4	
Density (calculated)	1.333 Mg · m^{-3}	
Absorption coefficient	0.552 mm^{-1}	
F(000)	1536 e	
Crystal size	0.134 x 0.055 x 0.013 mm^3	
θ range for data collection	1.295 to 31.688°.	
Index ranges	-18 ≤ h ≤ 17, -13≤ k ≤ 13, -46 ≤ l ≤ 46	
Reflections collected	95671	
Independent reflections	12216 [R_{int} = 0.0930]	
Reflections with I>2σ(I)	8261	
Completeness to θ = 25.242°	100.0 %	
Absorption correction	Gaussian	
Max. and min. transmission	0.99 and 0.94	
Refinement method	Full-matrix least-squares on F^2	
Data / restraints / parameters	12216 / 0 / 419	
Goodness-of-fit on F^2	1.012	
Final R indices [I>2σ(I)]	R_1 = 0.0489	wR^2 = 0.0837
R indices (all data)	R_1 = 0.0954	wR^2 = 0.0967
Largest diff. peak and hole	0.9 and -1.2 e · $Å^{-3}$	

Table S6. Bond lengths [Å] and angles [°] of complex **16**

Ru(1)-P(1)	2.3226(7)	Ru(1)-P(2)	2.3476(7)
Ru(1)-N(1)	2.146(2)	Ru(1)-C(1)	2.001(2)
Ru(1)-C(3)	1.924(3)	P(1)-C(15)	1.813(3)
P(1)-C(21)	1.859(3)	P(1)-C(24)	1.838(3)
P(2)-C(28)	1.809(2)	P(2)-C(34)	1.863(3)
P(2)-C(37)	1.841(3)	O(1)-C(5)	1.207(3)
O(2)-C(5)	1.368(3)	O(2)-C(6)	1.438(3)
N(1)-C(14)	1.389(3)	N(1)-C(27)	1.398(3)
C(1)-C(2)	1.434(3)	C(1)-C(7)	1.458(3)
C(2)-C(3)	1.419(4)	C(2)-C(5)	1.456(4)
C(3)-C(4)	1.485(4)	C(7)-C(8)	1.399(4)
C(7)-C(12)	1.402(4)	C(8)-C(9)	1.383(4)
C(9)-C(10)	1.385(4)	C(10)-C(11)	1.392(4)
C(10)-C(13)	1.503(4)	C(11)-C(12)	1.386(4)
C(14)-C(15)	1.422(3)	C(14)-C(19)	1.412(3)
C(15)-C(16)	1.397(3)	C(16)-C(17)	1.387(3)
C(17)-C(18)	1.394(4)	C(17)-C(20)	1.510(3)
C(18)-C(19)	1.383(3)	C(21)-C(22)	1.518(4)
C(21)-C(23)	1.526(4)	C(24)-C(25)	1.525(4)
C(24)-C(26)	1.534(4)	C(27)-C(28)	1.424(3)
C(27)-C(32)	1.406(3)	C(28)-C(29)	1.394(3)
C(29)-C(30)	1.391(4)	C(30)-C(31)	1.382(4)
C(30)-C(33)	1.509(3)	C(31)-C(32)	1.390(3)
C(34)-C(35)	1.528(4)	C(34)-C(36)	1.534(4)
C(37)-C(38)	1.523(4)	C(37)-C(39)	1.533(4)
P(1)-Ru(1)-P(2)	158.27(2)	N(1)-Ru(1)-P(1)	82.09(6)
N(1)-Ru(1)-P(2)	79.38(6)	C(1)-Ru(1)-P(1)	95.90(7)
C(1)-Ru(1)-P(2)	104.27(7)	C(1)-Ru(1)-N(1)	170.01(9)
C(3)-Ru(1)-P(1)	98.13(8)	C(3)-Ru(1)-P(2)	97.35(8)
C(3)-Ru(1)-N(1)	104.24(10)	C(3)-Ru(1)-C(1)	66.25(11)
C(15)-P(1)-Ru(1)	101.60(8)	C(15)-P(1)-C(21)	104.48(12)
C(15)-P(1)-C(24)	107.99(12)	C(21)-P(1)-Ru(1)	103.28(9)
C(24)-P(1)-Ru(1)	131.24(9)	C(24)-P(1)-C(21)	105.62(12)
C(28)-P(2)-Ru(1)	99.57(8)	C(28)-P(2)-C(34)	103.33(12)
C(28)-P(2)-C(37)	107.86(12)	C(34)-P(2)-Ru(1)	114.87(9)
C(37)-P(2)-Ru(1)	125.16(9)	C(37)-P(2)-C(34)	103.83(13)
C(5)-O(2)-C(6)	114.7(2)	C(14)-N(1)-Ru(1)	119.61(15)
C(14)-N(1)-C(27)	121.9(2)	C(27)-N(1)-Ru(1)	118.09(15)
C(2)-C(1)-Ru(1)	96.10(17)	C(2)-C(1)-C(7)	130.0(2)
C(7)-C(1)-Ru(1)	133.16(18)	C(1)-C(2)-C(5)	132.5(2)
C(3)-C(2)-C(1)	97.5(2)	C(3)-C(2)-C(5)	129.8(2)

C(2)-C(3)-Ru(1)	100.09(17)	C(2)-C(3)-C(4)	131.0(2)
C(4)-C(3)-Ru(1)	128.9(2)	O(1)-C(5)-O(2)	121.2(2)
O(1)-C(5)-C(2)	127.5(3)	O(2)-C(5)-C(2)	111.3(2)
C(8)-C(7)-C(1)	123.3(2)	C(8)-C(7)-C(12)	117.3(2)
C(12)-C(7)-C(1)	119.1(2)	C(9)-C(8)-C(7)	120.9(3)
C(8)-C(9)-C(10)	121.8(3)	C(9)-C(10)-C(11)	117.6(3)
C(9)-C(10)-C(13)	121.2(3)	C(11)-C(10)-C(13)	121.1(3)
C(12)-C(11)-C(10)	121.3(3)	C(11)-C(12)-C(7)	121.0(2)
N(1)-C(14)-C(15)	118.8(2)	N(1)-C(14)-C(19)	125.2(2)
C(19)-C(14)-C(15)	115.9(2)	C(14)-C(15)-P(1)	116.36(18)
C(16)-C(15)-P(1)	122.84(19)	C(16)-C(15)-C(14)	120.4(2)
C(17)-C(16)-C(15)	122.6(2)	C(16)-C(17)-C(18)	116.8(2)
C(16)-C(17)-C(20)	121.0(2)	C(18)-C(17)-C(20)	122.1(2)
C(19)-C(18)-C(17)	121.9(2)	C(18)-C(19)-C(14)	122.0(2)
C(22)-C(21)-P(1)	116.3(2)	C(22)-C(21)-C(23)	111.6(2)
C(23)-C(21)-P(1)	107.08(17)	C(25)-C(24)-P(1)	110.23(19)
C(25)-C(24)-C(26)	112.1(2)	C(26)-C(24)-P(1)	110.7(2)
N(1)-C(27)-C(28)	117.7(2)	N(1)-C(27)-C(32)	126.2(2)
C(32)-C(27)-C(28)	115.9(2)	C(27)-C(28)-P(2)	115.25(17)
C(29)-C(28)-P(2)	123.8(2)	C(29)-C(28)-C(27)	121.0(2)
C(30)-C(29)-C(28)	122.2(2)	C(29)-C(30)-C(33)	120.7(2)
C(31)-C(30)-C(29)	116.8(2)	C(31)-C(30)-C(33)	122.6(2)
C(30)-C(31)-C(32)	122.5(2)	C(31)-C(32)-C(27)	121.6(2)
C(35)-C(34)-P(2)	110.44(18)	C(35)-C(34)-C(36)	109.3(2)
C(36)-C(34)-P(2)	113.29(19)	C(38)-C(37)-P(2)	111.1(2)
C(38)-C(37)-C(39)	111.7(2)	C(39)-C(37)-P(2)	109.17(19)

Experimental Procedures

General Information

All reactions were carried out under argon in flame-dried glassware using standard Schlenk techniques unless stated otherwise.[1] Air-sensitive compounds were stored and transferred under Ar. The solvents were purified by distillation over the drying agents indicated and were transferred under Ar: tetrahydrofuran (magnesium/anthracene), toluene ($NaAlEt_4$), benzene (CaH_2), dichloromethane (CaH_2), acetonitrile (CaH_2), diethyl ether (Na/K), *n*-hexane (Na/K), *n*-pentane (Na/K) and hexamethyldisiloxane (CaH_2). Deuterated solvents were degassed by three freeze-pump-thaw cycles and were then stored over molecular sieves (3 Å). The molecular sieves were dried for 24 h at 150°C (sand bath) under vacuum (10^{-3} mbar) prior to use and were stored and transferred under Ar. All commercially available compounds (abcr, BLD, Aldrich, TCI) were used as received, unless stated otherwise. 3-(Phenylethynyl)oxazolidin-2-one (**13**)[2] and cyclooctyne (**17**)[3] were prepared according to the literature procedures.

NMR spectra were acquired on a Bruker Avance III 400 MHz, 500 MHz, or an Avance Neo 600 MHz NMR (equipped with a Bruker BBO CryoProbe) spectrometer in the solvent indicated. Chemical shifts (δ) are given in ppm relative to peaks of residual undeuterated solvent ($CDCl_3$: δ_H = 7.26 ppm; δ_C = 77.16 ppm; CD_2Cl_2: δ_H = 5.32 ppm; δ_C = 53.84 ppm; C_6D_6: δ_H = 7.16 ppm; δ_C = 128.06 ppm; [D_8]-toluene: δ_H = 2.09 ppm; δ_C = 20.43 ppm; [D_3]-MeCN: δ_H = 1.94 ppm; δ_C = 1.32 ppm).[4] $^{31}P\{^1H\}$ chemical shifts are reported relative 85% H_3PO_4 (δ = 0 ppm; Ξ = 40.480742%). 1D ^{29}Si NMR spectra were acquired with a polarization transfer pulse sequence (refocused INEPT) and broadband proton decoupling. ^{29}Si chemical shifts were referenced indirectly to the ^1H chemical shift of the solvent according to IUPAC recommendations using the xiref macro in Bruker Topspin. ^{29}Si chemical shifts are reported relative to TMS (δ = 0 ppm; Ξ = 19.867187%). Coupling constants (*J*) are given in Hz.

IR spectra were recorded on an ALPHA (Bruker) FT-IR spectrometer with an ATR unit; the signals are given in wavenumbers (ṽ) in cm^{-1}.

HRMS data were recorded on a Bruker APEX III FT-MS (7T magnet), MAT 95 (Finnigan), Thermo Scientific LTQ-FT, or Thermo Scientific Exactive Spectrometer.

Elemental analyses were carried out by Mikroanalytisches Laboratorium Kolbe, Oberhausen, Germany.

Alkylidyne Synthon and PNP Pincer Ligand

The alkylidyne synthon **8** was prepared in three steps by modified literature procedures. [5,6]

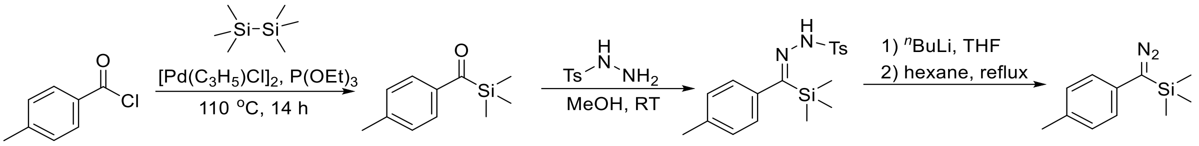

S1 (87%) **S2** (72%) **1** (96 %)

***p*-Tolyl(trimethylsilyl)methanone (S1).**[7] A Schlenk flask equipped with a stir bar was charged

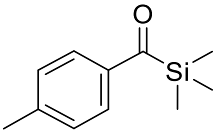

with $[Pd(C_3H_5)Cl]_2$ (229 mg, 0.62 mmol), triethyl phosphite (0.415 g, 2.5 mmol) and Si_2Me_6 (3.95 g, 27.0 mmol). The resulting mixture was stirred for 5 min to give a pale yellow suspension. Freshly distilled *p*-methylbenzoyl chloride (3.87 g, 25.0 mmol) was then added to the suspension, forming a clear yellow solution. The mixture was degassed by three freeze-pump-thaw cycles, the flask was refilled with Ar and the mixture stirred at 110 °C (bath temperature) for 14 h. The mixture was filtered through a plug of Celite to remove Pd black, which was rinsed with pentane. The combined organic phases were evaporated using a rotary evaporator. The residue was purified by Kugelrohr distillation (110°C at 10^{-3} bar; the temperature must not exceed 125°C to avoid decomposition), yielding the title compound as a yellow liquid (4.190 g, 87%). ^{1}H NMR (400 MHz, $CDCl_3$) δ 7.75 (app d, *J* = 8.1 Hz, 2H), 7.27 (app d, *J* = 7.9 Hz, 2H), 2.41 (s, 3H), 0.37 (s, 9H). ^{13}C{^{1}H} NMR (101 MHz, $CDCl_3$) δ 235.0, 143.6, 139.3, 129.5, 127.8, 21.8, −1.2.
The spectroscopic data are consistent with those reported in the literature.[7]

***p*-Tolyl(trimethylsilyl)methanone tosylhydrazone (S2).**[6] This reaction was conducted in air.

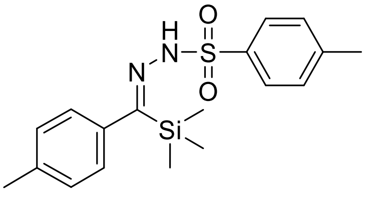

MeOH (2 mL) was added at room temperature to a vigorously stirred suspension of toluene-*p*-sulfonyl hydrazide (3.725 g, 20 mmol) and *p*-tolyl(trimethylsilyl)methanone (3.847 g, 20 mmol). The suspension initially turned clear within minutes before it solidified in seconds while the yellow color faded away. *Note*: *This reaction is exothermic*. Once the yellow color disappeared, the flask was immediately immersed in an ice bath. The resulting white solid material was ground into a powder, washed with cold MeOH (2 × 5 mL) and dried under vacuum to give the title compound as a white solid (5.214 g, 72%). ^{1}H NMR (400 MHz, $CDCl_3$) δ 7.78 (app d, *J* = 8.3 Hz, 2H), 7.67 (br s, 1H), 7.31 (app d, *J* = 8.1 Hz, 2H), 7.20 (app d, *J* = 7.7 Hz, 2H), 6.74 (app d, *J* = 8.1 Hz, 2H), 2.45 (s, 3H), 2.36 (s, 3H), 0.08 (s, 9H). ^{13}C{^{1}H} NMR (101 MHz, $CDCl_3$) δ 168.7, 144.0, 138.9, 135.8, 131.5, 130.4, 129.5, 128.1, 126.0, 21.8, 21.4, –2.2.
The spectroscopic data are consistent with those reported in the literature.[6]

***p*-Tolyl(trimethylsilyl)diazomethane (8).**[6] A three-necked flask equipped with a condenser

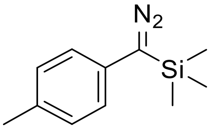

was charged with *p*-tolyl(trimethylsilyl)methanone tosylhydrazone (5.000 g, 13.9 mmol) and THF (45 mL). The mixture was cooled to 0 °C before *n*BuLi (1.6 M, 9.1 mL, 14.6 mmol) was added dropwise. Once the addition was

complete, the mixture was stirred at 0 °C for 1 h. The volatile materials were removed under vacuum. The residue was suspended in hexane (100 mL) and the resulting mixture was stirred at reflux temperature (80°C bath temperature) for 3 h. The reaction was quenched with wet pentane at 0 °C. The red solution was filtered through a plug of Celite to remove inorganic salts, and the filtrate was evaporated on a rotary evaporator to afford the title compound as a red oil (2.711 g, 96%). ^{1}H NMR (400 MHz, $CDCl_3$) δ 7.14 – 7.08 (m, 2H), 6.92 (app d, *J* = 8.2 Hz, 2H), 2.31 (s, 3H), 0.34 (s, 9H). ^{1}H NMR (400 MHz, CD_2Cl_2) δ 7.14 – 7.09 (m, 2H), 6.91 (app d, *J* = 8.2 Hz, 2H), 2.29 (s, 3H), 0.33 (s, 9H). ^{1}H NMR (400 MHz, C_6D_6) δ 6.96 (s, 4H), 2.11 (s, 3H), 0.13 (s, 9H). ^{13}C{^{1}H} NMR (101 MHz, $CDCl_3$) δ 133.1, 130.1, 129.9, 123.1, 41.7 (brs, *C*N_2), 21.0, –0.8. IR (ATR, neat) 2958, 2922, 2032, 1509, 1283, 1252, 1174, 940, 835, 805, 753, 633, 612, 497 cm^{-1}. HRMS-EI (*m/z*): calcd. for $C_{11}H_{16}N_2Si^+$ [M^+]: 204.1077; found: 204.1076.

Lithium Amide 10. *n*-BuLi (1.6 M in hexane, 46.9 mL, 75.0 mmol) was slowly added to a solution of bis(2-bromo-4-methylphenyl)amine (8.875 g, 25.0 mmol) in Et_2O (250 mL) at –35 °C. The mixture was allowed to warm to room temperature and stirred for 3 h before it was cooled back to –35 °C. Diisopropylchlorophosphine (7.630 g, 50.0 mmol) was added and the resulting mixture was allowed to warm to room temperature while stirring. After 24 h, the volatiles were removed under vacuum and the orange residue was suspended in toluene (100 mL) and the suspension filtered through a pad of Celite. THF (5 mL) was added to the filtrate and the resulting mixture was stirred for 30 min at room temperature. The volatiles were removed under vacuum and the reddish residue was washed with pentane (4 × 25 mL) and dried under vacuum to afford the title compound as a yellow solid (6.352 g). The pentane filtrates were combined and stored at –20 °C for 2 d, yielding the second crop of the product as orange crystals, which were collected by filtration, washed with cold pentane (3 mL × 4) and dried under vacuum (1.924 g). Total yield: 8.276 g, 65%. ^{1}H NMR (400 MHz, C_6D_6) δ 7.63 (dd, *J* = 8.4, 5.2 Hz, 2H), 7.06 (dd, *J* = 4.6, 2.2 Hz, 2H), 7.00 (dd, *J* = 8.4, 2.2 Hz, 2H), 3.44 – 3.35 (m, 4H), 2.32 (s, 6H), 2.02 (pd, *J* = 7.0, 4.0 Hz, 4H), 1.22 – 1.17 (m, 4H), 1.17 – 1.03 (m, 24H). ^{13}C{^{1}H} NMR (101 MHz, C_6D_6) δ 161.3 (d, *J* = 18.7 Hz), 132.8 (d, *J* = 2.4 Hz), 131.7, 121.5 (d, *J* = 2.3 Hz), 119.4 (d, *J* = 10.1 Hz), 116.6 (d, *J* = 4.1 Hz), 68.2, 25.4, 22.7 (d, *J* = 2.5 Hz), 21.2, 20.3 (d, *J* = 13.2 Hz), 19.8 (d, *J* = 8.9 Hz). ^{31}P{^{1}H} NMR (162 MHz, C_6D_6) δ –4.6 (q, J_{7LiP} = 48.4 Hz).

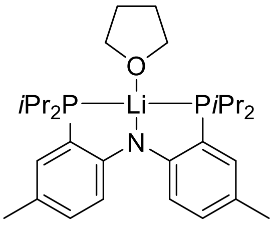

The spectroscopic data are consistent with those reported in the literature.[8]

Synthesis of the Ruthenium Complexes

{Ru[=C(*p*-tolyl)TMS]Cl(NCMe)$_2$}$_2$(*μ*-Cl)$_2$ (9). A three-necked flask equipped with a gas inlet, an oil bubbler and a stir bar was charged with [(*p*-cymene)$RuCl_2$]$_2$ (5.171 g, 8.4 mmol) and degassed MeCN (80 mL). The mixture was stirred until a homogeneous orange solution had formed. *p*-Tolyl(trimethylsilyl)-diazomethane (**8**) (3.630 g, 17.8 mmol) was then added dropwise to the solution. After stirring at room temperature for 30 min, a green crystalline solid started to precipitate from the mixture. After 5 h, the green solid material was collected by filtration, washed with MeCN (3 × 5 mL), toluene (10 mL) and pentane (3 × 10 mL), and dried under vacuum to give the title complex as a green crystalline solid (5.651 g, 78%).

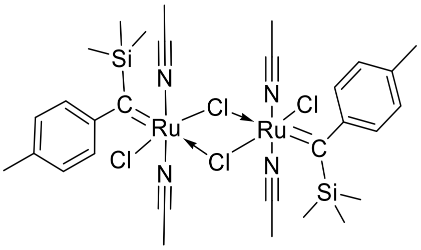

Single crystals suitable for X-ray diffraction analysis were obtained by performing the reaction in an NMR tube: To an NMR tube were added 1.2 mg of [(*p*-cymene)$RuCl_2$]$_2$, CH_2Cl_2 (0.5 mL) and MeCN (0.1 mL). The tube was shaken to obtain a homogeneous solution. An aliquot (0.12 mL) of a stock solution of *p*-tolyl(trimethylsilyl)diazomethane (prepared by dissolving 5 mg of **8** in 0.5 mL of *n*-hexane) was added, the tube was sealed, shaken and allowed to stand at room temperature overnight. The resulting crystals were suitable for X-ray diffraction analysis.

Since the compound is only sparingly soluble in acetonitrile, only the ^{1}H NMR spectrum could be recorded. In CD_3CN solution, the compound forms a pair of CD_3CN-ligated *cis-trans* isomers, which are tentatively assigned as {**Ru[=C(*p*-tolyl)TMS]Cl$_2$(NCMe)$_3$**} containing three (deuterated) acetonitrile ligands, see copies of spectra. ^{1}H NMR (400 MHz, CD_3CN) δ [7.25 (d, J = 7.8 Hz), 7.15 (d, J = 7.8 Hz), 2H], [6.79 (d, J = 8.1 Hz), 6.70 (d, J = 8.0 Hz), 2H], [2.36 (s), 2.31 (s), 3H], 1.96 (s, 6H, free CH_3CN), [0.20 (s), 0.16 (s), 9H]. IR (ATR, powder): 2968, 2917, 1496, 1415, 1245, 1021, 947, 909, 877, 834, 795, 763, 743, 685, 622, 512, 459 cm^{-1}. Anal. calcd. for $C_{30}H_{44}Cl_4N_4Ru_2Si_2$: C 41.86, H 5.15, N 6.51; found: C 41.72, H 5.11, N 6.48.

{Ru(≡C-*p*-tolyl)(^{iPr}PNP)} (12). A Schlenk flask was charged with complex **9** (430 mg, 0.50 mmol), lithium amide **10** (533 mg, 1.05 mmol) and toluene (15 mL). The mixture was stirred at room temperature for 30 min before the flask was immersed in an oil bath preheated to 90 °C. After stirring at this temperature for another 30 min, the mixture was cooled to room temperature, all volatile materials were evaporated under vacuum, and the resulting brown residue was extracted with pentane (3 × 3 mL). The combined pentane extracts were concentrated to a total volume of ca. 5 mL, to which was added hexamethyldisiloxane (HMDSO, 5 mL). The mixture was kept at room temperature for 2 d. The precipitated dark green crystals were collected by filtration and washed with HMDSO (3 × 1 mL). The green crystals were extracted with pentane (4 × 5 mL) and the combined pentane extracts were evaporated under vacuum to afford the desired alkylidyne complex as a dark green solid (480 mg, 76%).

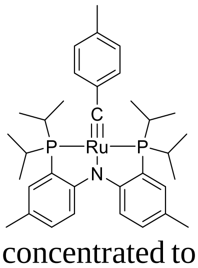

Single crystals suitable for X-ray diffraction analysis were obtained by storing a dilute solution of the complex in pentane at –20 °C for a week.

^{1}H NMR (400 MHz, C_6D_6) δ 7.72 (d, *J* = 7.9 Hz, 2H), 7.46 (dt, *J* = 8.5, 2.1 Hz, 2H), 7.23 – 7.17 (m, 2H), 6.82 – 6.76 (m, 2H), 6.61 (d, *J* = 7.8 Hz, 2H), 2.73 – 2.53 (m, 4H), 2.23 (s, 6H), 1.72 (s, 3H), 1.39 – 1.29 (m, 24H). ^{13}C{^{1}H} NMR (101 MHz, C_6D_6) δ 262.9 (t, *J* = 13.7 Hz), 161.8 (t, *J* = 13.0 Hz), 146.1, 138.7, 132.2, 131.9, 129.2, 126.9, 125.1 (t, *J* = 3.2 Hz), 123.6 (t, *J* = 17.6 Hz), 115.9 (t, *J* = 5.3 Hz), 26.7 (t, *J* = 11.5 Hz), 21.9, 20.4 (t, *J* = 3.7 Hz), 19.3. ^{31}P{^{1}H} NMR (162 MHz, C_6D_6) δ 68.06. Anal. calcd. for $C_{34}H_{47}NP_2Ru$: C 64.54, H 7.49, N 2.21; found: C 64.12, H 7.49, N 2.21.

Ruthenacyclobutadiene 14. A Schlenk tube was charged with the Ru alkylidyne complex **12** (76 mg, 0.12 mmol), 3-(phenylethynyl)oxazolidin-2-one (**13**) (19 mg, 0.10 mmol) and toluene (5 mL). After stirring the mixture at room temperature for 30 min, the reaction was complete as indicated by ^{1}H NMR spectroscopy. All volatile materials were removed under vacuum and the residue was washed with pentane (3 × 3 mL) and dried under vacuum to give a first crop of the title complex as a black solid (52 mg). The combined pentane filtrates were left standing at room temperature for 2 d, leading to the precipitation of additional black solid material (23 mg), which was collected, washed with pentane (3 × 1 mL) and dried under vacuum. Total yield: 75 mg, 91%. ^{1}H NMR (600 MHz, C_6D_6) δ 8.12 (dt, *J* = 8.6, 2.2 Hz, 2H), 7.24 – 7.19 (m, 4H), 7.13 (d, *J* = 8.1 Hz, 2H), 7.12 – 7.07 (m, 3H), 6.97 (dd, *J* = 8.6, 2.0 Hz, 2H), 6.46 (d, *J* = 7.9 Hz, 2H), 3.26 – 3.20 (m, 2H), 3.12 – 3.07 (m, 2H), 2.63 – 2.51 (m, 4H), 2.28 (s, 6H), 1.50 (s, 3H), 1.30 (td, *J* = 7.6, 6.9 Hz, 6H), 1.14 – 1.05 (m, 12H), 1.00 (dt, *J* = 6.9, 6.1 Hz, 6H). ^{13}C{^{1}H} NMR (151 MHz, C_6D_6) δ 213.7 (t, *J* = 11.1 Hz), 205.9 (t, *J* = 10.5 Hz), 162.3 (t, *J* = 10.3 Hz), 158.5, 150.8 (t, *J* = 5.3 Hz), 143.5 (t, *J* = 0.9 Hz), 136.1, 138.6 (t, *J* = 2.3 Hz), 132.5, 131.3, 130.0 (t, *J* = 2.2 Hz), 129.08 (brs), 129.06, 126.4, 123.6 (t, *J* = 3.0 Hz), 123.3 (t, *J* = 17.2 Hz), 117.0 (t, *J* = 5.0 Hz), 61.6, 47.6, 27.0 (t, *J* = 10.1 Hz), 24.0 (t, *J* = 10.0 Hz), 21.6, 20.77, 19.8 (t, *J* = 3.3 Hz), 19.2 (t, *J* = 1.0 Hz), 18.4 (t, *J* = 3.1 Hz), 17.8. ^{31}P{^{1}H} NMR (243 MHz, C_6D_6) δ 47.0. Anal. calcd. for $C_{45}H_{56}N_2O_2P_2Ru$: C 65.92, H 6.88, N 3.42; found: C 65.06, H 6.89, N 3.39.

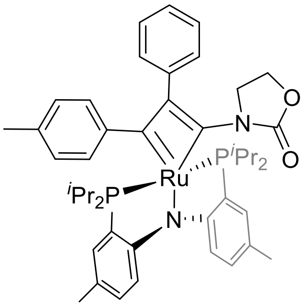

Ruthenacyclobutadiene 16. A Schlenk tube was charged with the Ru alkylidyne complex **12** (101 mg, 0.16 mmol), methyl 2-butynoate (**15**) (24 mg, 0.24 mmol), and toluene (5 mL). After stirring of the solution at room temperature for 30 min, the reaction was complete as indicated by ^{31}P NMR spectroscopy. The volatiles were removed under vacuum, and the residue was extracted with pentane (4 × 5 mL). The combined pentane extracts were concentrated to a total volume of ca. 5 mL and the solution was stored at –20 °C for 3 d. The red crystals deposited on the bottom of the flask were collected by filtration, washed with cold pentane (3 × 1 mL) and dried under vacuum to give the title complex as a red solid material (105 mg, 90%). ^{1}H NMR (600 MHz, C_6D_6) δ 8.01 – 7.95 (m, 2H), 7.96 – 7.91 (m, 2H), 6.95 – 6.90 (m, 6H), 3.56 (s, 3H), 2.39 – 2.28 (m, 2H), 2.25 (s, 6H), 2.24 – 2.16 (m, 2H), 1.76 (s, 3H), 1.56 (t, *J* = 1.3 Hz, 3H), 1.22 – 1.14 (m, 6H), 1.00 (q, *J* = 7.2 Hz, 6H),

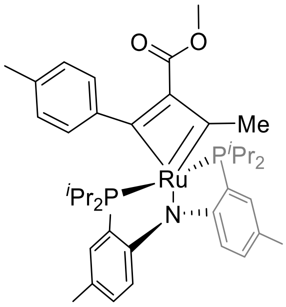

0.92 – 0.85 (m, 6H), 0.80 (q, *J* = 6.7 Hz, 6H). $^{13}C\{^{1}H\}$ NMR (151 MHz, C_6D_6) δ 273.6 (t, *J* = 9.8 Hz), 259.6 (t, *J* = 9.7 Hz), 162.0 (t, *J* = 10.1 Hz), 156.7 (t, *J* = 4.8 Hz), 150.6 (t, *J* = 2.5 Hz), 149.1, 137.1, 132.7, 131.9, 129.5, 127.2, 123.9 (t, *J* = 3.1 Hz), 122.1 (t, *J* = 17.8 Hz), 116.5 (t, *J* = 4.9 Hz), 49.4, 41.0, 25.7 (t, *J* = 10.6 Hz), 25.2 (t, *J* = 10.9 Hz), 22.0, 20.6, 19.0 (t, *J* = 2.8 Hz), 18.6 (t, *J* = 3.1 Hz), 18.5 (t, *J* = 1.1 Hz), 17.9. $^{31}P\{^{1}H\}$ NMR (162 MHz, C_6D_6) δ 44.7. Anal. calcd. for $C_{39}H_{53}NO_2P_2Ru$: C 64.09, H 7.31, N 1.92; found: C 63.71, H 7.30, N 1.93.

Spectroscopic Observation of the Intermediate 11

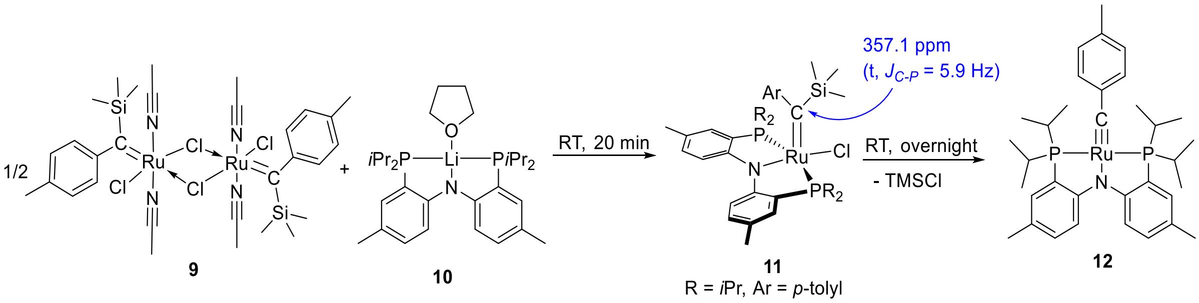

A J-Young NMR tube was charged with Ru carbene complex **9** (8.0 mg), ligand **10** (8.0 mg) and [D$_8$]-toluene (0.7 mL) under Ar. The NMR tube was sealed and shaken for 20 min at room temperature, resulting in the formation of a brown solution. The sample was transferred to a Bruker 600 MHz NMR spectrometer, the magnet of which was pre-cooled to 0 °C (273 K). After shimming, tuning and matching, the spectra were acquired. In the ^{31}P{^{1}H} NMR spectrum, the intermediate **11** displays a broad singlet at δ_P = 46.3 ppm. Ru alkylidyne complex **12,** which resonates at δ_P = 67.7 ppm, has already been formed (Figure S4).

Upon warming the NMR tube to room temperature, more Ru alkylidyne complex **12** is gradually formed. After standing overnight, the sample was subjected to the same Bruker 600 MHz NMR magnet and spectra were recorded at 273 K. The ^{31}P{^{1}H} NMR spectrum indicated that the conversion of **11** into **12** was essentially complete (Figure S4).

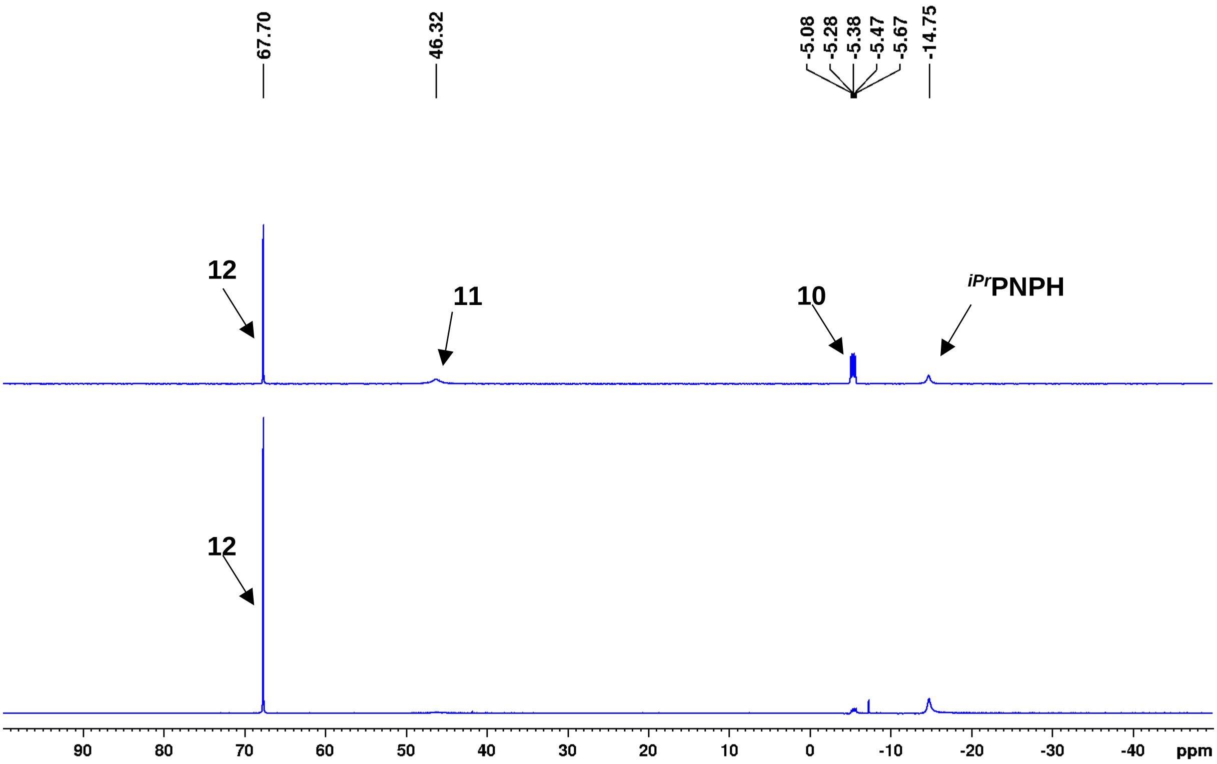

Figure S4. Stacked ^{31}P{^{1}H} NMR spectra of the reaction of the Ru carbene complex **9** with the ligand **10** after 20 min (**a**) and overnight (**b**) at room temperature. Both spectra were recorded at 273 K in [D$_8$]-toluene.

The $^{13}C\{^1H\}$ and ^1H-^{13}C HMBC NMR spectra of the intermediate **11** were recorded at 273 K. The resonance of the carbene carbon of **11** appears at 357.1 ppm (t, J_{C-P} = 5.9 Hz), which has a cross peak in the ^1H-^{13}C HMBC spectrum with the signal of TMS protons (δ_H = 0.55 ppm) (Figure S5). The chemical shift of the TMS protons in ^1H NMR spectrum was determined by a ^1H-^{29}Si HMBC spectrum, in which the signal of the TMS protons (δ_H = 0.55 ppm) has a cross peak with the signal of the Si atom (δ_{Si} = 1.66 ppm) (Figure S6). These results strongly support the formation of the silylated Ru carbene complex **11**.

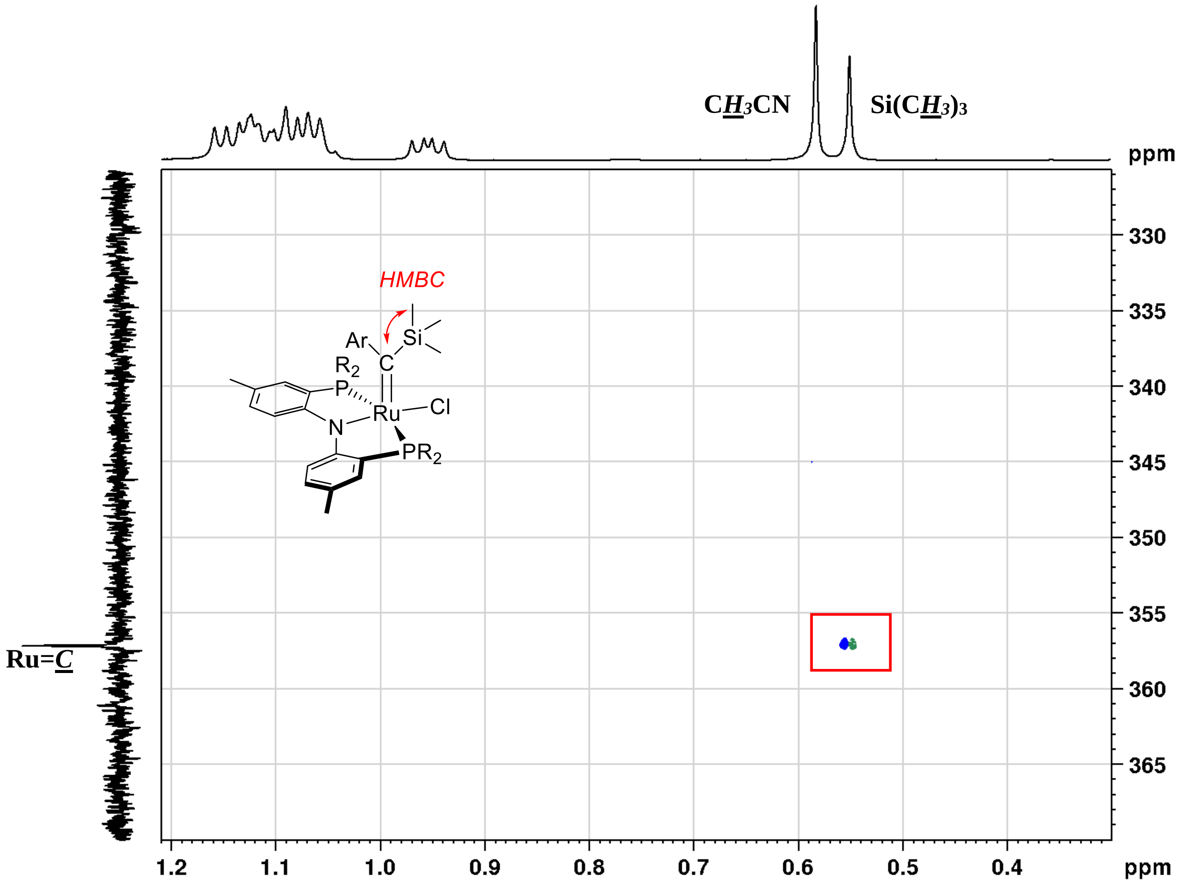

Figure S5. Relevant part of the ^1H-^{13}C HMBC spectrum of the silylated ruthenium carbene **11** in [D8]-toluene at 273K, showing three-bond correlation between the carbene C-atom and the protons of the TMS group.

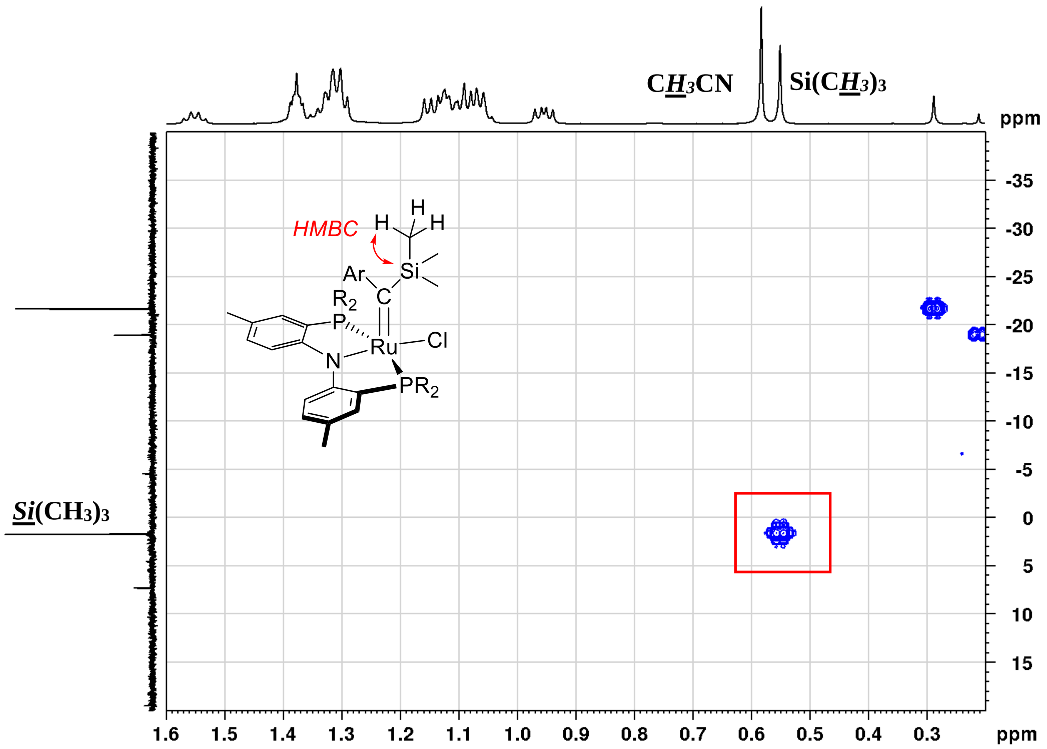

Figure S6. Relevant part of the ^{1}H-^{29}Si HMBC spectrum of the silylated ruthenium carbene **11** in [D_8]-toluene at 273K, showing the two-bond correlation between the Si atom and the protons of the TMS group.

[2+2] Cycloaddition of the Ruthenium Alkylidyne 12 with Cyclooctyne

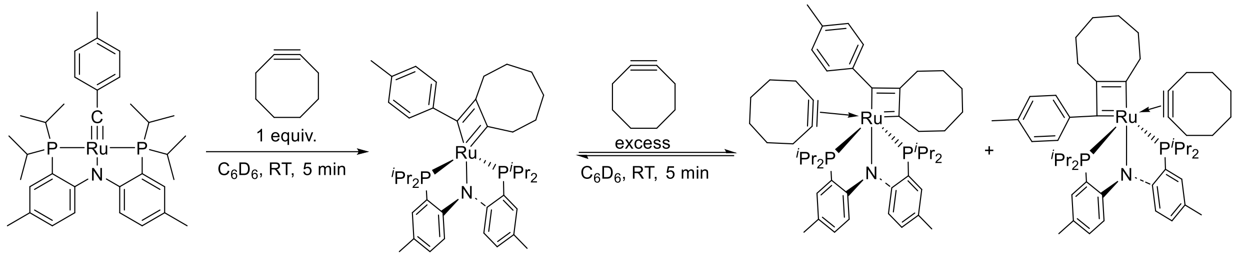

12 **18** **19a** **19b**

Upon addition of cyclooctyne (**17**, 1 equiv.) to a solution of the alkylidyne complex **12** in C_6D_6, the color changed immediately from brown to dark red. According to NMR, the expected ruthenacyclobutadiene complex **18** was formed quantitatively. Complex **18** displays a sharp singlet at δ_P = 42.9 ppm in the $^{31}P\{^1H\}$ NMR spectrum in C_6D_6 (Figure S7, b). Signals in the ^{13}C NMR spectrum at δ_C = 253.0 (t, *J* = 10.6 Hz), 214.2 (t, *J* = 10.5 Hz) and 170.0 (t, *J* = 5.5 Hz) ppm are characteristic of the three carbons on a metallacyclobutadiene (MCBD) ring. Six sets of multiplets in the region between 2.89 – 1.43 ppm in the ^1H NMR spectrum show that all CH_2 groups are magnetically inequivalent.

Upon addition of more cyclooctyne (0.5 equiv.), the sharp singlet at δ_P = 42.9 ppm was broadened, and two additional (broad) singlets were detected at δ_P = 41.2 and 39.4 ppm (Figure S7, c).

The reaction of alkylidyne complex **12** with a large excess of cyclooctyne (10 equiv.) led only to these two new species with the broad singlets at δ_P = 41.2 and 39.4 ppm (Figure S7, d). These new complexes are assigned as the corresponding adducts **19a** and **19b**, see below. The ratio **19a**:**19b** was 1:0.87 in C_6D_6 at 298 K.

When the solution containing only **19a** and **19b** and excess cyclooctyne was evaporated under high vacuum and the residue was re-dissolved in C_6D_6, the signal of the MCBD complex **18** re-appeared in the mixture. This observation suggests that the formation of **19a,b** is reversible.

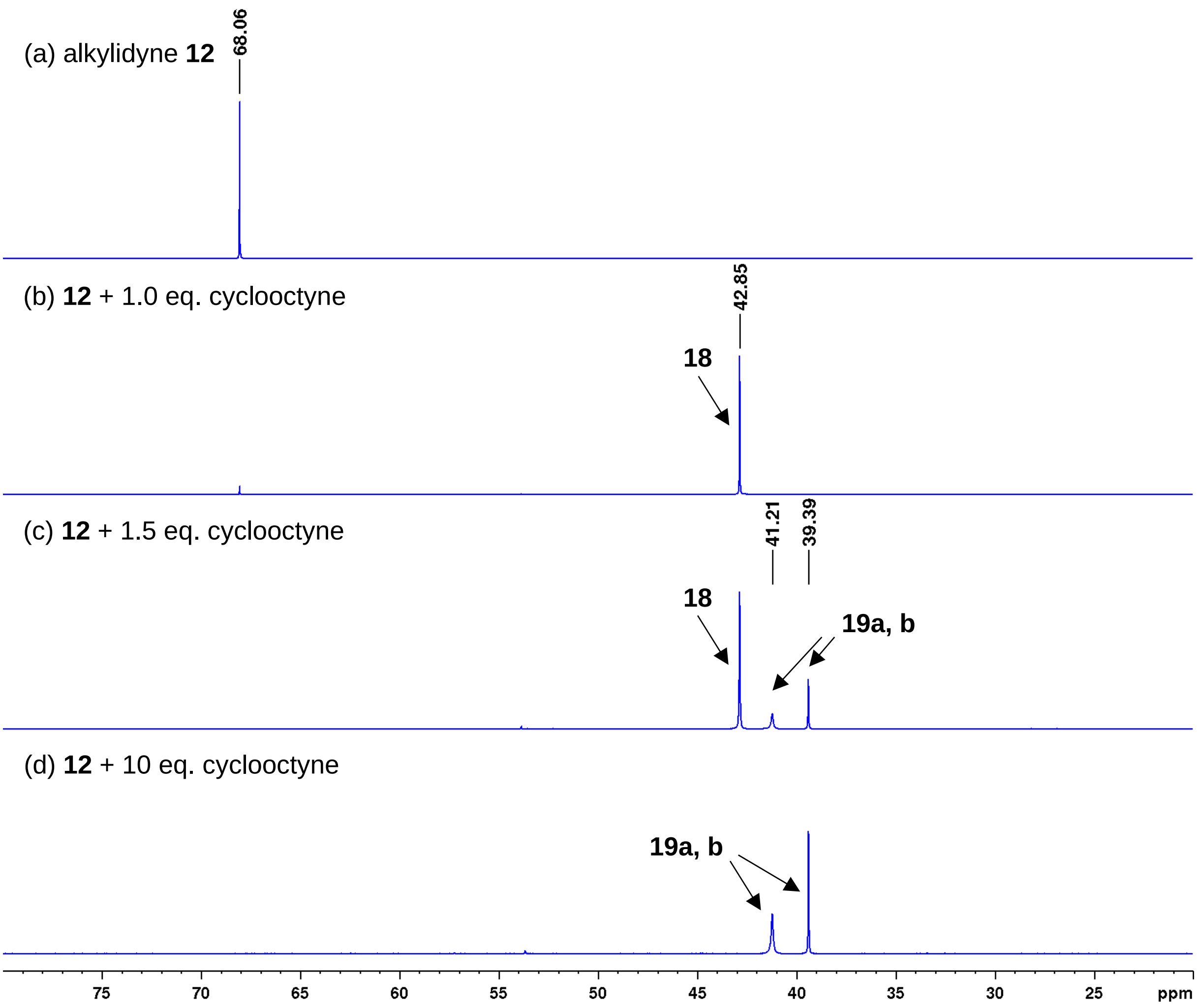

Figure S7. Stacked $^{31}P\{^1H\}$ NMR spectra of the reaction of the Ru alkylidyne complex **12** with varying amounts of cyclooctyne (1.0 (**b**), 1.5 (**c**), and 10 equiv. (**d**)); for reference, the $^{31}P\{^1H\}$ NMR spectrum of the Ru alkylidyne complex **12** is shown in (**a**).

Complexes **19a** and **19b** exhibit similar $^{13}C\{^1H\}$ NMR patterns as the parent MCBD complex **18**, implying that they must have similar structures. Specifically, both show characteristic deshielded resonances at δ_C = 253.9, 227.5, 168.5 ppm and δ_C = 264.1, 223.3, 167.9 ppm, respectively (Figure S8). These deshielded ^{13}C NMR signals have cross peaks with either the protons of the *p*-tolyl group or protons of the aliphatic chain in the ^1H-^{13}C HMBC NMR spectrum (Figure S9), which indicates that **19a** and **19b** are also MCBD complexes with the same substituents as complex **18**.

In addition, complexes **19a** and **19b** display two sets of carbon resonances at δ_C = 90.1/88.6 ppm, and δ_C = 89.1/87.9 ppm, respectively (Figure S8). The chemicals shifts are similar to the shift of the alkyne C-atoms of free cyclooctyne (δ_C = 94.7 ppm, C_6D_6) and are therefore assigned to cyclooctyne ligated to the MCBDs.[9] This assignment is supported by an HRMS analysis of the mixture, which fits to such adducts as it shows a peak corresponding to the composition [**12**+cyclooctyne]$^+$. The fact that **19a** and **19b** each shows two signals for the C-

atoms of the ligated cyclooctyne suggests that the rotation about the Ru–alkyne(midpoint) axis is slow on the NMR timescale. On the other hand, adduct formation is reversible since evaporation of all volatiles regenerated a mixture comprising the bare ruthenacyclobutadiene complex **18**.

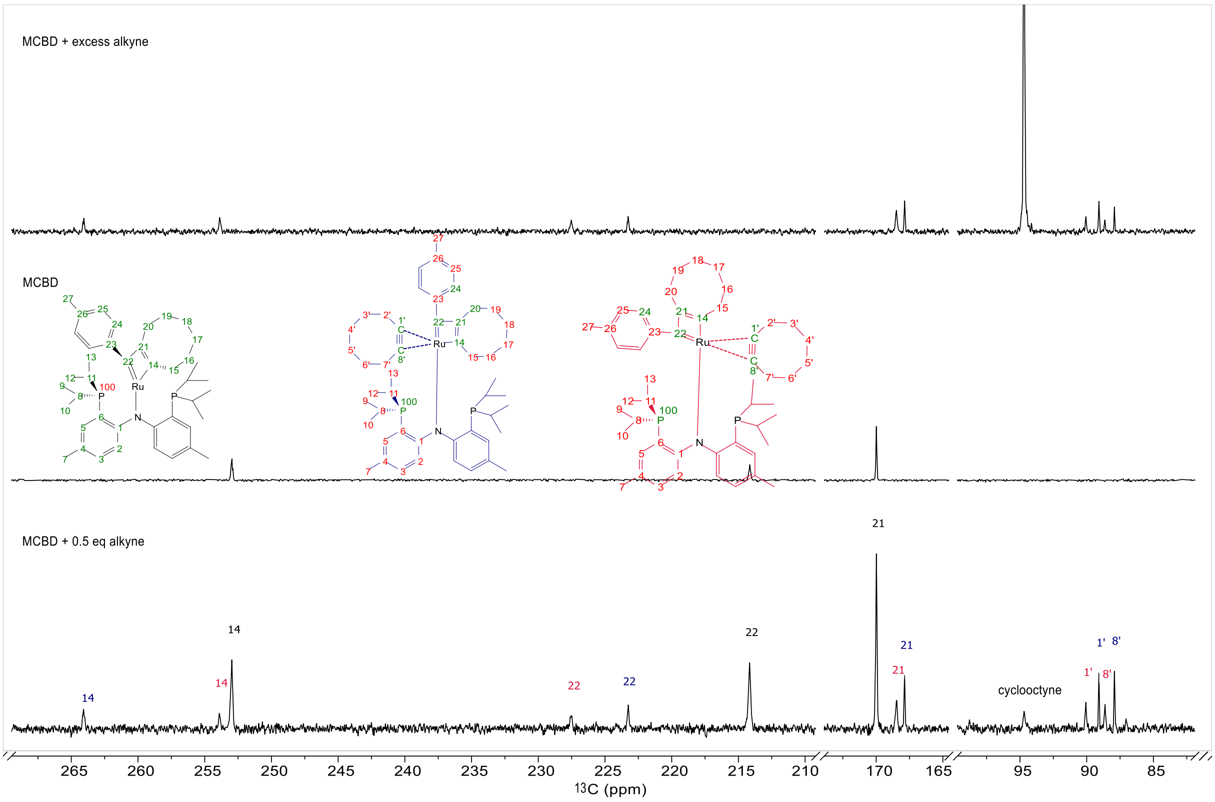

Figure S8. Stacked $^{13}C\{^{1}H\}$ NMR spectra showing regions of interest: top: a mixture of two cyclooctyne-coordinated MCBDs **19a** and **19b** formed by reaction of the Ru alkylidyne complex **12** with 10 equiv. of cyclooctyne; middle: the bare MCBD complex **18** formed on reaction of **12** with 1 equiv. of cyclooctyne; bottom: a mixture of complexes **18**, **19a** and **19b** formed on reaction of **12** with 1.5 equiv. of cyclooctyne.

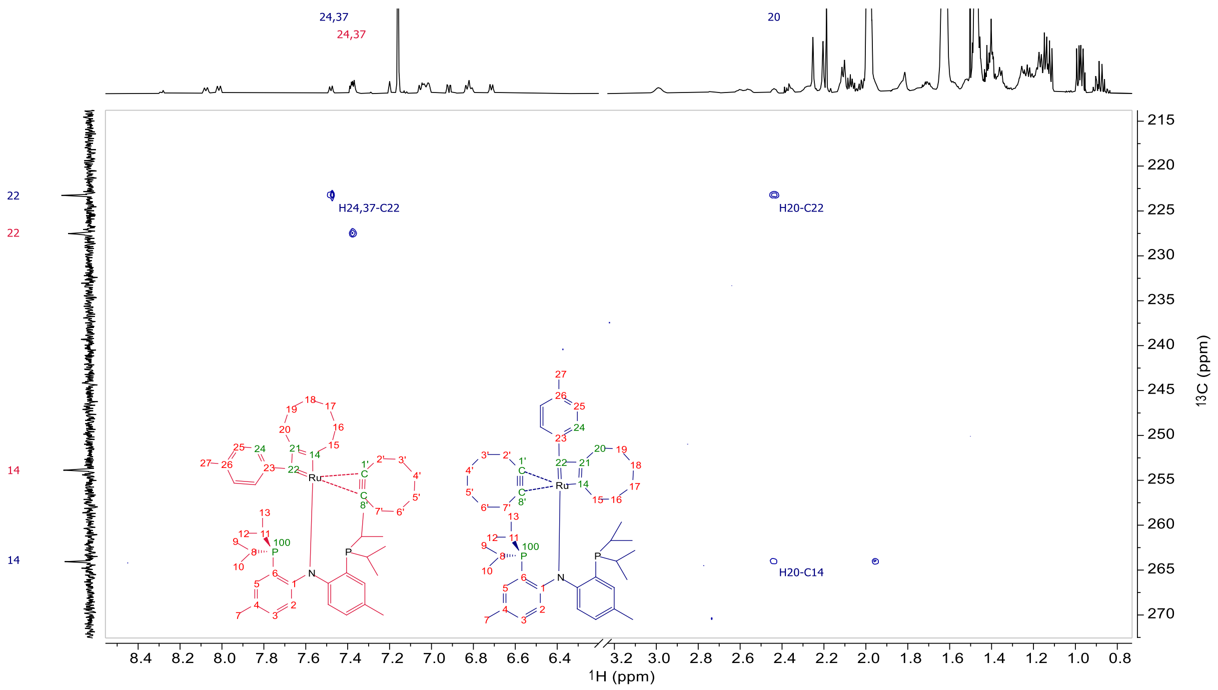

Figure S9. Part of the ^{1}H-^{13}C HMBC NMR spectrum of a mixture of two cyclooctyne-coordinated MCBDs **19a** and **19b** formed on reaction of the Ru alkylidyne complex **18** with 10 equiv. of cyclooctyne.

Sample Preparation. A stock solution (0.1 M) of cyclooctyne in C_6D_6 was prepared by dissolving cyclooctyne (12.6 µL, 0.1 mmol) in C_6D_6 (1 mL).

An NMR tube was then charged with the Ru alkylidyne complex **12** (7.8 mg) and C_6D_6 (0.5 mL) followed by the addition of an aliquot of the cyclooctyne stock solution (0.1 M, 110 µL), which caused an immediate color change from brown to dark red. A $^{31}P\{^1H\}$ NMR spectrum was recorded, which showed a sharp singlet at δ_P = 42.9 ppm. A full NMR characterization of this compound was performed, which was assigned the metallacyclobutadiene structure **18**.

Next, an additional aliquot of the cyclooctyne stock solution (60 µL) was added to the NMR tube, resulting in the formation of three species resonating at δ_P = 42.9, 41.2, and 39.4 ppm with a ratio of 100:30:27. This sample was subject to ^1H–^1H and ^{31}P–^{31}P EXSY NMR measurements (see below) and also used for HRMS measurements.

A seperate NMR tube was charged with the Ru alkylidyne complex **12** (6.3 mg) and C_6D_6 (0.6 mL). Neat cyclooctyne (12.6 µL, 10 equiv.) was added. The $^{31}P\{^1H\}$ NMR spectrum showed two signals at δ_P = 41.2 and 39.4 ppm. Full NMR characterization of this sample was performed.

Characterization data of the bare ruthenacyclobutadiene complex **18**: ^1H NMR (600 MHz, C_6D_6) δ 8.02 (dt, *J* = 8.6, 2.3 Hz, 2H), 7.33 – 7.26 (m, 2H), 7.05 – 7.02 (m, 2H), 6.95 (dd, *J* = 8.6, 2.1 Hz, 2H), 6.78 – 6.74 (m, 2H), 2.89 – 2.84 (m, 2H), 2.47 – 2.34 (m, 4H), 2.28 (s, 6H), 1.79 – 1.68 (m, 4H), 1.65 (s, 3H), 1.68 – 1.61 (m, 2H), 1.62 – 1.56 (m, 2H), 1.49 – 1.43 (m, 2H), 1.15 (q, *J* = 7.3 Hz, 6H), 1.12 – 1.05 (m, 12H), 0.81 (q, *J* = 7.0 Hz, 6H). $^{13}C\{^1H\}$ NMR (151 MHz, C_6D_6) δ 253.0 (t, *J* = 10.6 Hz), 214.2 (t, *J* = 10.5 Hz), 170.0 (t, *J* = 5.5 Hz), 162.3 (t, *J* = 10.0 Hz), 147.9, 134.3, 132.6, 131.5, 130.1, 124.9 (br s), 123.6 (t, *J* = 2.9 Hz), 123.2 (t, *J* = 16.6 Hz), 116.4 (t, *J* = 4.7 Hz), 52.1, 29.2 (t, *J* = 2.6 Hz), 28.0 (t, *J* = 2.0 Hz), 27.6, 25.9, 25.8 (t, *J* = 9.4 Hz), 25.0 (t, *J* = 9.9 Hz), 23.4, 22.0, 20.7, 19.5 (t, *J* = 3.3 Hz), 18.6 (t, *J* = 3.1 Hz), 18.3 (t, *J* = 1.2 Hz), 18.2. $^{31}P\{^1H\}$ NMR (243 MHz, C_6D_6) δ 42.9. HRMS-ESI (*m/z*): calcd. for $C_{42}H_{59}NP_2Ru^+$ [M^+]: 741.31607; found: 741.31578.

Since the aliphatic region of the mixture of the two exchanging cyclooctyne-bound MCBD complexes is very complicated (excess cyclooctyne is present in the mixture) and most peaks are broad, only characteristic signals of the two complexes **19a** and **19b** are given: ^1H NMR (600 MHz, C_6D_6) δ [8.08 (br d, *J* = 8.7 Hz), 8.01 (br d, *J* = 8.7 Hz), 2H], [7.48 (br d, *J* = 7.8 Hz), 7.40 – 7.32 (m), 2H], 7.07 – 6.99 (m, 2H), 6.79 – 6.85 (m, 2H), [7.05 (br d, *J* = 8.1 Hz), 6.71 (br d, *J* = 7.9 Hz), 2H], [2.25 (br s), 2.20 (br s), 3H]. $^{13}C\{^1H\}$ NMR (151 MHz, C_6D_6) δ 264.1 (t, *J* = 10.3 Hz), 253.9 (br), 227.5 (br), 223.3 (t, *J* = 9.8 Hz), 168.5 (br), 167.9 (t, *J* = 5.3 Hz), 90.1 (br), 89.1, 88.6 (br), 87.9. $^{31}P\{^1H\}$ NMR (243 MHz, C_6D_6) δ 41.2 (br), 39.4 (br). HRMS-ESI (*m/z*): calcd. for $C_{50}H_{71}NP_2Ru^+$ [M^+]: 849.40898; found: 849.40998.

Tautomerization of the Ruthenacyclobutadienes

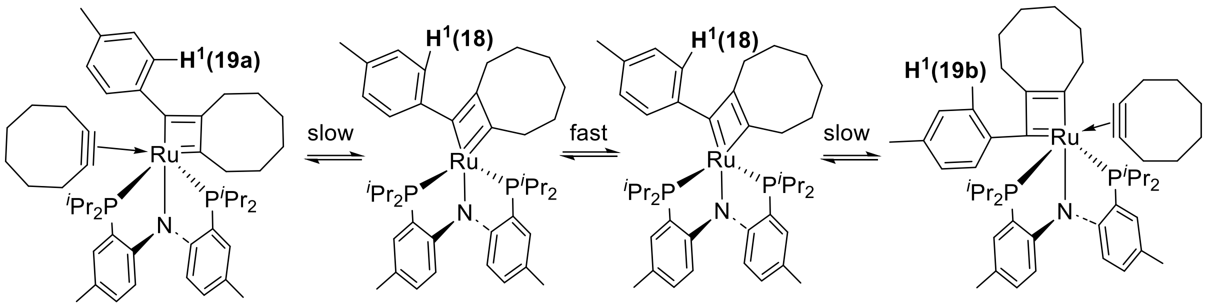

Analysis. The interconversion of the three MCBD complexes **18**, **19a** and **19b** was observed by ^{1}H-^{1}H and ^{31}P-^{31}P EXSY NMR experiments. Figure S10 shows the aromatic region of the ^{1}H-^{1}H EXSY NMR spectrum of the mixture. The *ortho*-protons on the tolyl groups of **18** and **19a** as well as **18** and **19b** are in exchange with each other. Figure S11 shows excerpts of the ^{31}P-^{31}P EXSY NMR spectra of the mixture recorded with different mixing times. At a mixing time of 300 ms, cross peaks of all three species were recorded (Figure S11, right). When the mixing time was reduced to 30 ms, only the exchange between **18/19a** and **18/19b** could be observed (Figure S11, left), indicating that no direct interconversion between the two cyclooctyne-bound MCBD complexes **19a** and **19b** takes place. Their interconversion hence proceeds via cyclooctyne dissociation, tautomerization at the stage of the five-coordinate MCBD intermediate **18** and subsequent re-coordination of a cyclooctyne ligand.

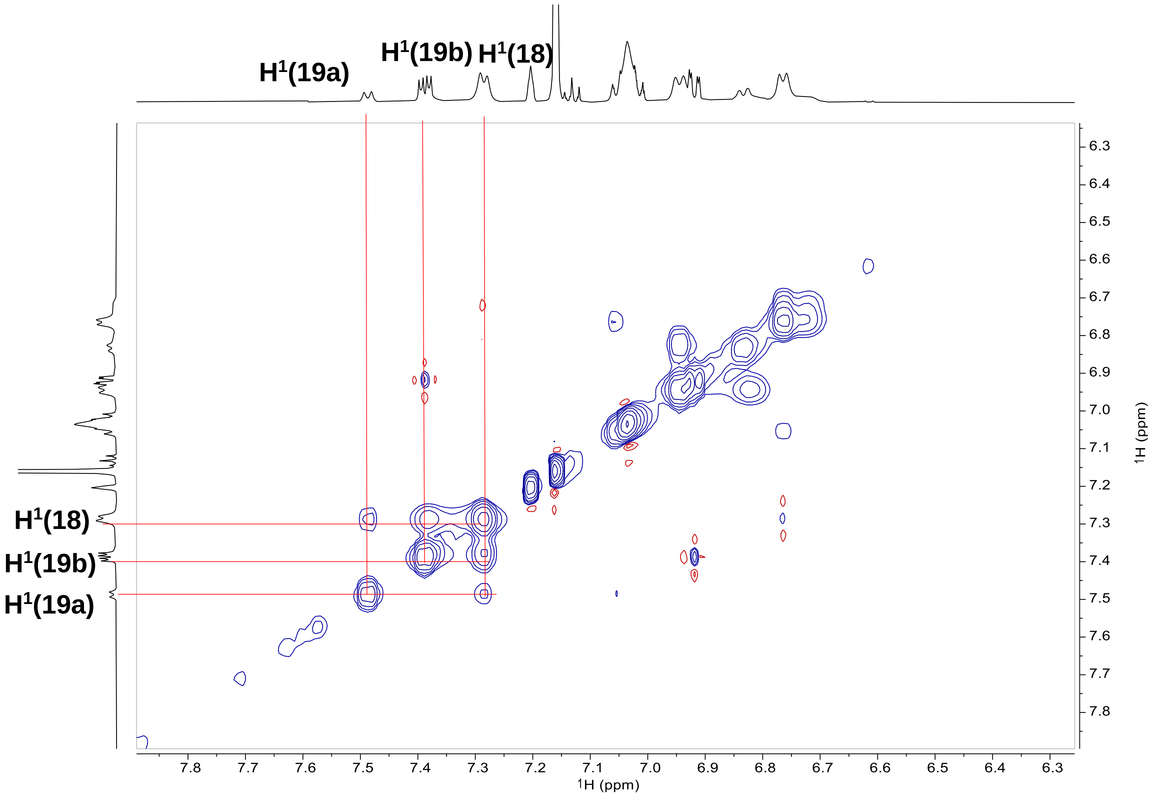

Figure S10. Part of the ^{1}H-^{1}H EXSY spectrum of the mixture of the three MCBDs **18**, **19a** and **19b** formed on reaction of the Ru alkylidyne complex **12** with 1.5 equiv. of cyclooctyne. Mixing time: 30 ms.

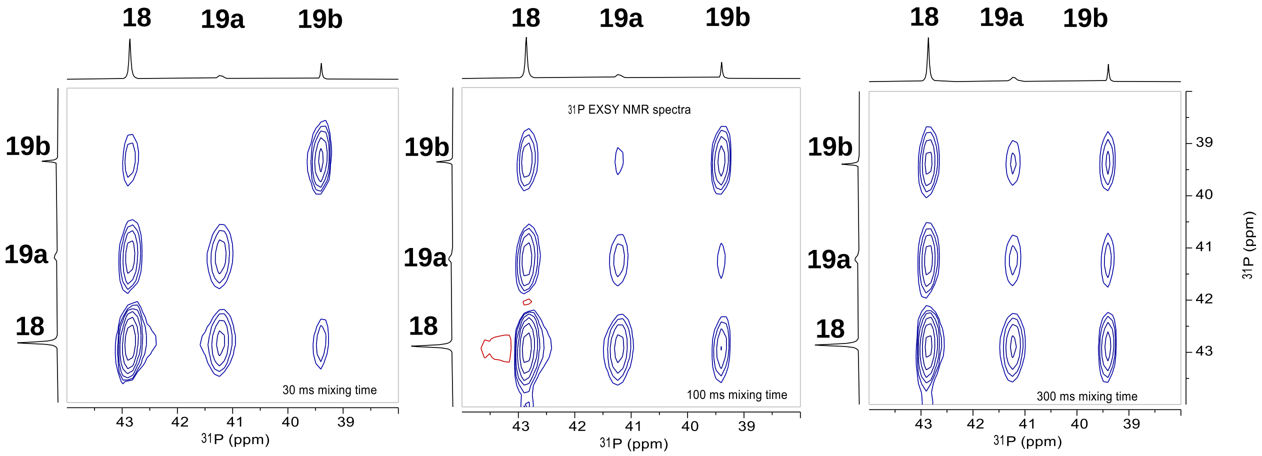

Figure S11. ^{31}P-^{31}P EXSY spectra of the mixture of the three MCBDs **18**, **19a** and **19b** recorded using different mixing times of 30 ms (left), 100 ms (middle), and 300 ms (right). The spectra are plotted with the same intensity threshold.

Spectroscopic Evidence for a Stepwise [2+2] Cycloaddition

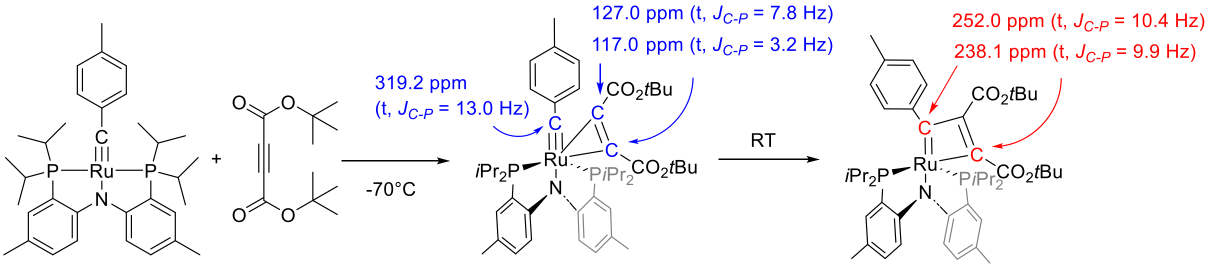

A J-Young NMR tube was charged with the Ru alkylidyne complex **12** (12.6 mg, 0.02 mmol) and di-*tert*-butyl acetylenedicarboxylate (**22**) (6.8 mg, 0.03 mmol) under Ar. Upon addition of 0.6 mL of [D8]-toluene into the NMR tube at room temperature, a dark green solution was formed immediately. The NMR tube was sealed and quickly transferred to a Bruker 500 MHz NMR spectrometer, the magnet of which was pre-cooled to –70 °C (203 K). NMR spectra suggested exclusive formation of one Ru complex. Signals in the ^{13}C NMR spectrum at δ_C = 127.0 (t, *J* = 7.8 Hz), 117.0 (t, *J* = 3.2 Hz) ppm hint to an η^2-coordinated alkyne, whereas a resonance at δC = 319.2 (t, *J* = 13.0 Hz) ppm shows the presence of an alkylidyne C-atom (Figure S12). These results strongly support the formation of the Ru carbyne-alkyne complex **23**. At 203 K, alkyne rotation is frozen, as evidenced by the two magnetically inequivalent alkyne carbons as well as two inequivalent COO-*t*-Bu groups. When the NMR tube was warmed to room temperature, the signals of two COO-*t*-Bu groups are merged, suggesting that the coordinated alkyne is rotate freely on the NMR timescale.

Upon warming the NMR tube to room temperature, the MCBD **24** was gradually formed, as evidenced by the characteristic resonances at δ_C = 252.0 (t, *J* = 10.4 Hz) and 238.1 (t, *J* = 9.9 Hz) ppm (Figure S13). After about 14 h, the conversion of **23** into **24** was complete.

Characterization data of the Ru alkylidyne-alkyne complex **23**: ^{1}H NMR (500 MHz, [D8]-toluene, 203K) δ 8.27 (br s, 2H), 7.83 – 7.72 (m, 2H), 6.90 – 6.76 (m, 4H), 6.68 (br d, *J* = 7.9 Hz, 2H), 2.46 – 2.40 (m, 1H), 2.37 – 2.28 (m, 1H), 2.28 (s, 3H), 2.24 (s, 3H), 1.93 – 1.85 (m, 1H), 1.69 (s, 3H), 1.65 (s, 9H), 1.62 – 1.56 (m, 1H), 1.41 (s, 9H), 1.54 – 1.13 (m, 12H), 1.07 – 0.58 (m, 12H). ^{1}H NMR (600 MHz, [D8]-toluene, 298K) δ 8.19 (app d, *J* = 8.1 Hz, 2H), 7.60 (dt, *J* = 8.5, 2.1 Hz, 2H), 6.87 – 6.82 (m, 2H), 6.80 (dd, *J* = 8.7, 2.1 Hz, 2H), 6.78 – 6.72 (m, 2H), 2.50 – 2.42 (m, 2H), 2.20 (s, 6H), 1.94 – 1.91 (m, 2H), 1.81 (s, 3H), 1.46 (br s, 18H), 1.28 – 1.13 (m, 18H), 0.86 (q, *J* = 6.8 Hz, 6H). ^{13}C{^{1}H} NMR (126 MHz, [D8]-toluene, 203K) δ 319.0 (t, *J* = 13.0 Hz), 167.6, 166.1, 161.8 (d, *J* = 17.9 Hz), 161.2 (d, *J* = 16.6 Hz), 146.3 (d, *J* = 2.7 Hz), 143.6, 132.7, 131.4, 131.3, 131.1, 130.9, 127.0 (t, *J* = 7.8 Hz), 123.7 – 123.3 (m), 122.0, 121.7, 117.0 (t, *J* = 3.2 Hz), 115.2 (d, *J* = 8.9 Hz), 114.4 (d, *J* = 9.1 Hz), 79.4, 77.5, 28.2, 28.1, 21.8 – 21.2 (m), 21.1 – 20.3 (m), 19.7 – 14.8 (m). ^{31}P{^{1}H} NMR (202 MHz, [D8]-toluene, 203 K) δ 64.2 (d, *J* = 303.1 Hz), 50.4 (d, *J* = 302.8 Hz). ^{31}P{^{1}H} NMR (202 MHz, [D8]-toluene, 298 K) δ 57.4 (br).

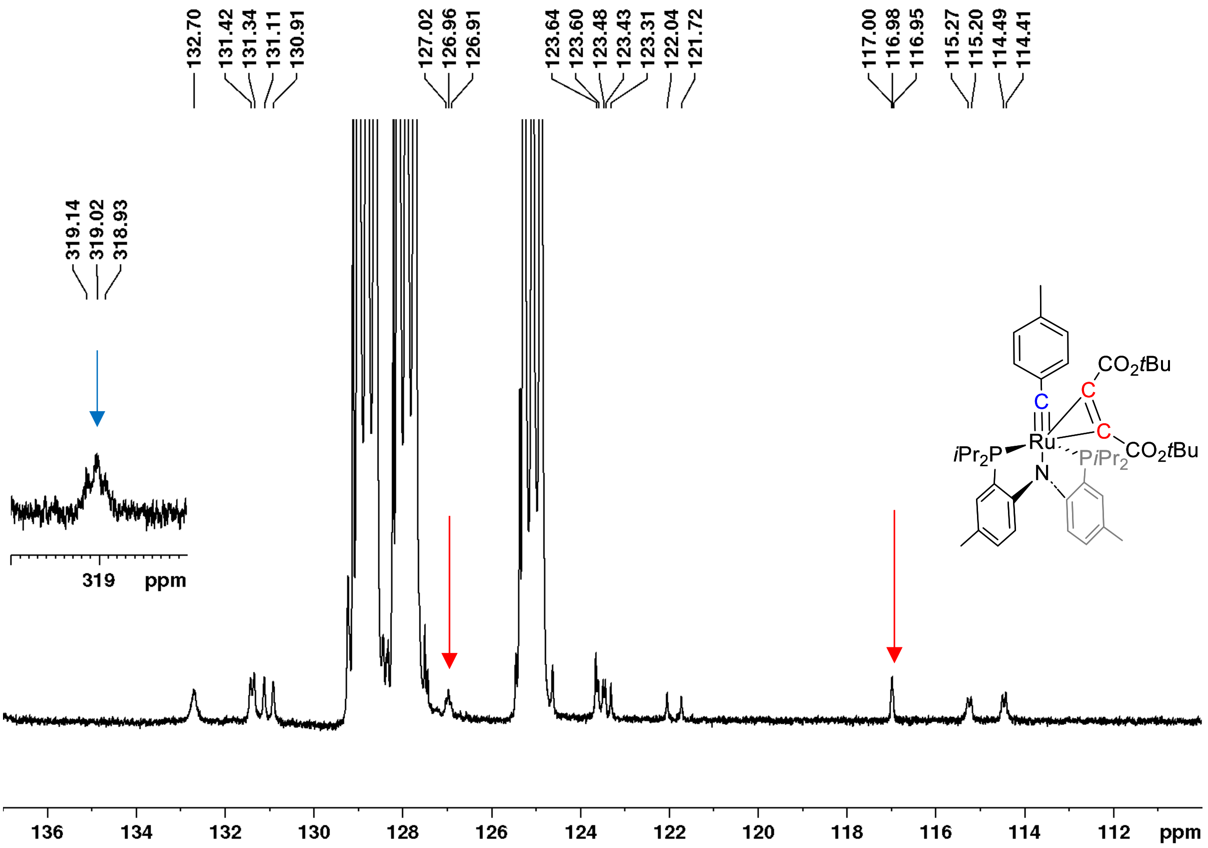

Figure S12. Partial $^{13}C\{^{1}H\}$ NMR spectrum of ruthenium alkyne-alkylidyne complex **23** in [D8]-toluene at 126 MHz at 203 K

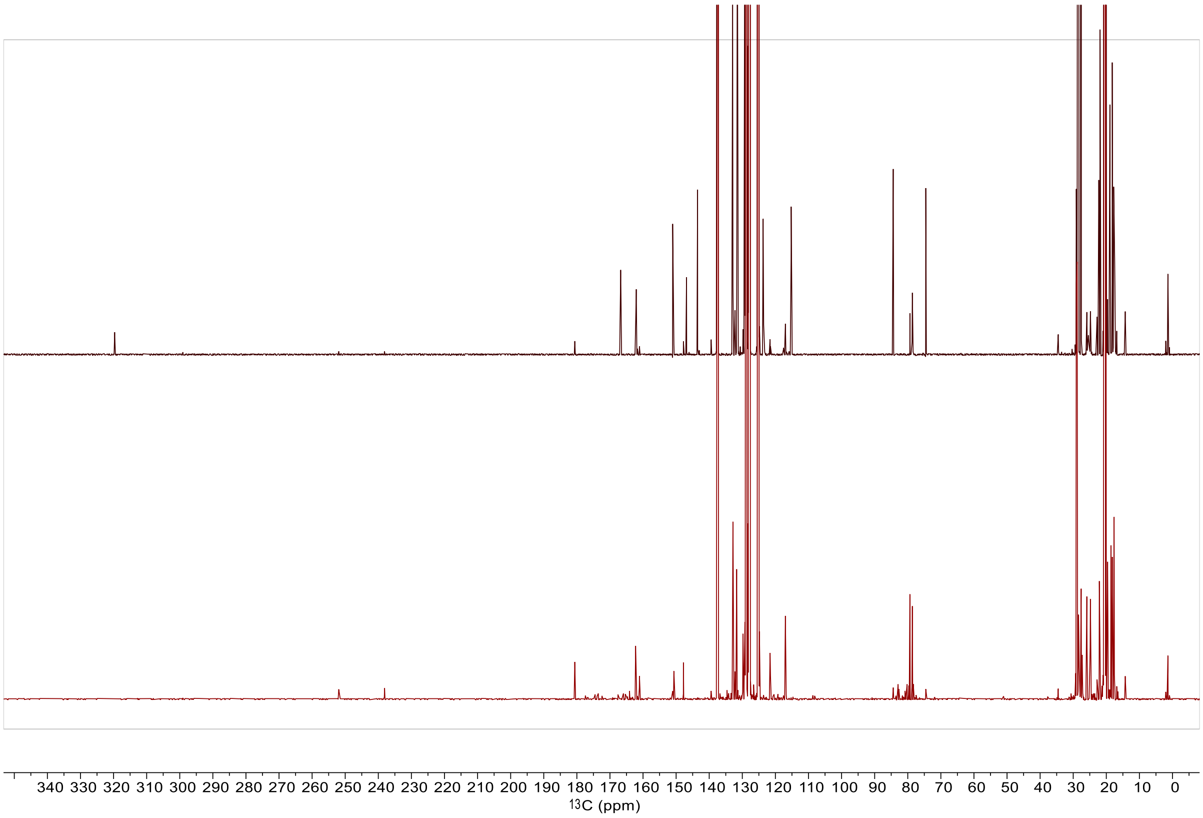

Figure S13. Stacked $^{13}C\{^{1}H\}$ NMR spectra of ruthenium alkylidyne **12** with di-*tert*-butyl acetylenedicarboxylate in [D8]-toluene at 151 MHz at 298 K. Top: RT, 2 h; bottom: RT, 14 h

Computational Study

Computational Details

All electronic structure calculations were performed using the ORCA 6.0 program package.[10] Geometry optimizations were conducted at the B3LYP-D4/def2-tzvp CPCM(toluene) level of theory[11] using tight convergence and large grid criteria. Minima were confirmed via normal mode analysis. Free energy corrections at 298.15K have been computed using the standard harmonic oscillator approximation as implemented in the thermochemistry module of ORCA.[12]

Sample input parameters for geometry optimization and frequency calculation:

```
!B3LYP D4 def2-tzvp CPCM(toluene) TightOpt TightSCF DefGrid3 Freq
```

Sample input parameters for transition state optimization and frequency calculation:

```
!B3LYP D4 def2-tzvp Def2/J CPCM(toluene) OptTS TightSCF DefGrid3 Freq

%geom
inhess Read
InHessName "molecule.hess"
end
```

Molecular Orbital Analysis of the Ru Alkylidyne Complex 12

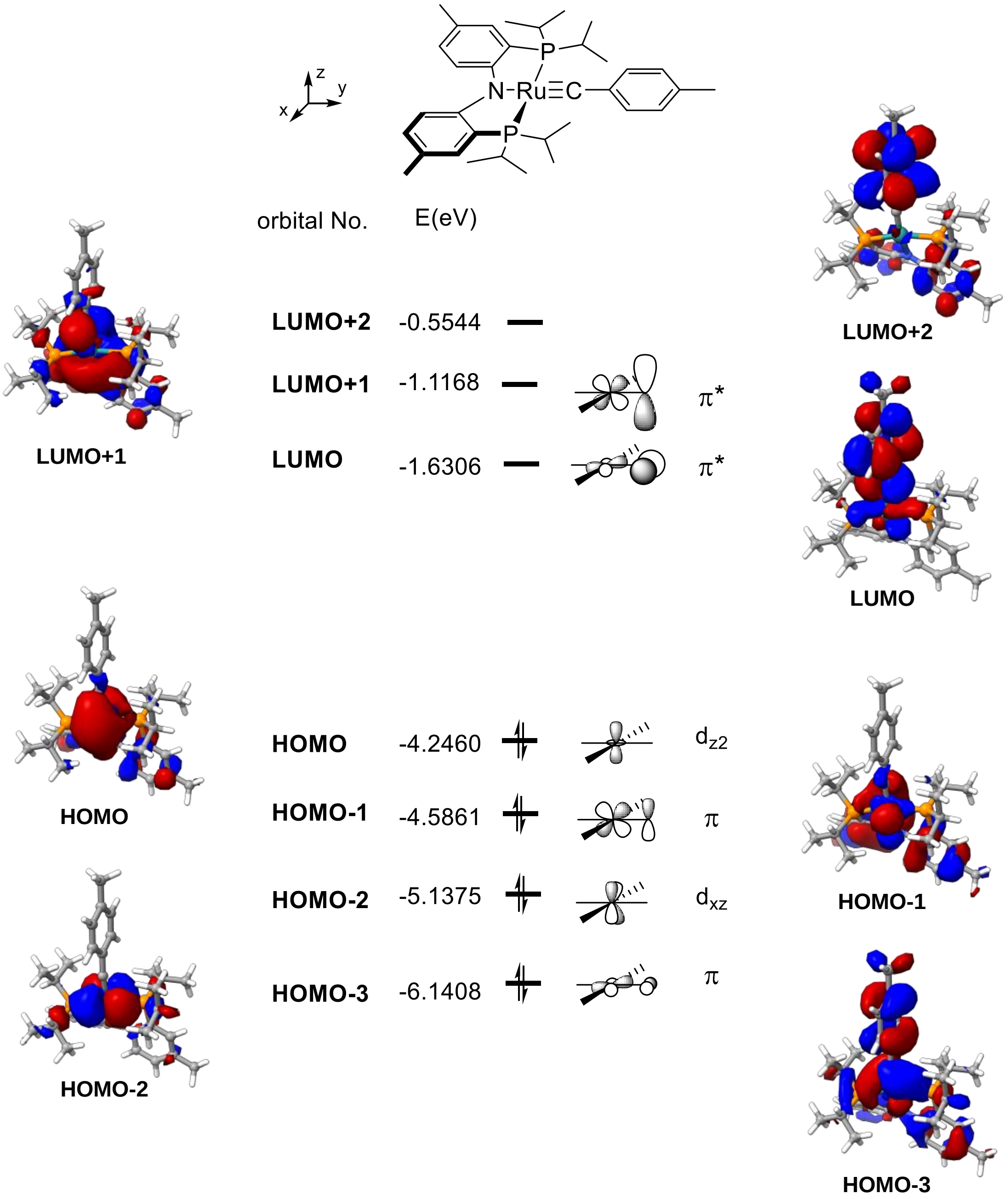

Figure S14. Frontier MO diagram of the Ru alkylidyne complex **12**

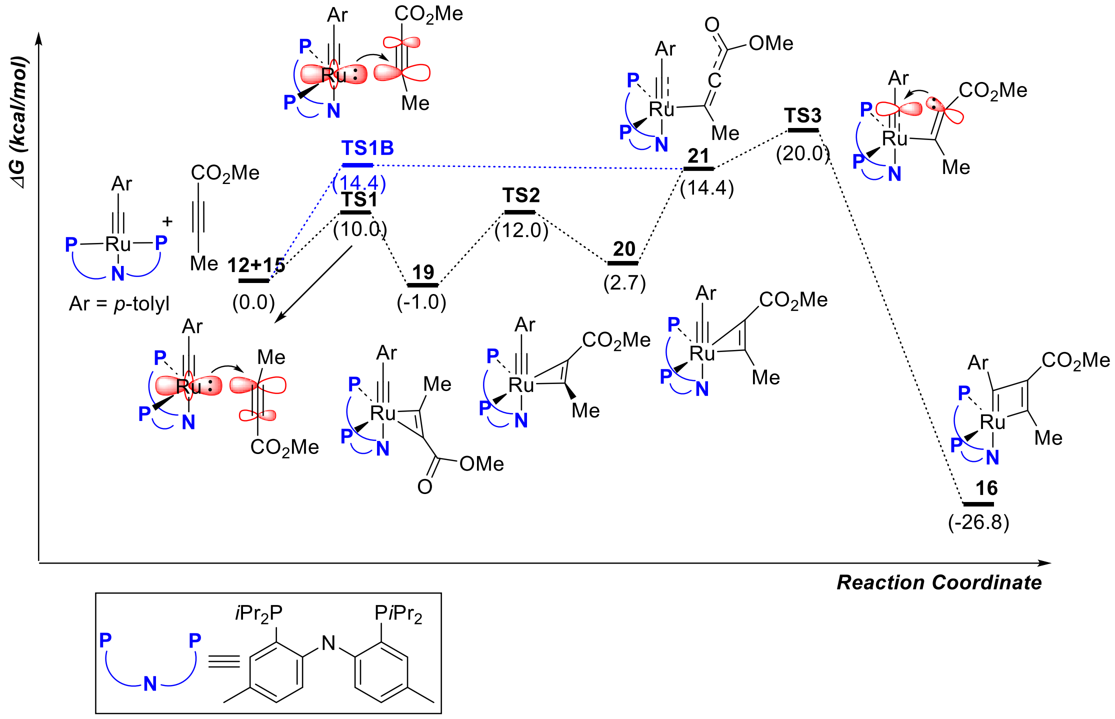

Figure S15. Computed energy profile of the [2+2] cycloaddition of the Ru alkylidyne complex **12** and methyl 2-butynoate **(15)** to form the ruthenacyclobutadiene complex **16**. Values in parentheses refer to the computed free energy differences in kcal/mol. The reaction pathway indicated in black represents the computationally predicted lowest-energy pathway, while the pathway in blue (via **TS1B**) shows the scenario where the alkyne is attacked in the opposite orientation relative to the alkylidyne unit.

In analogy to the formation of ruthenacyclobutadiene **16** carrying an electron-withdrawing group shown in Figure S15, the formation of ruthenacyclobutadiene **14** from the reaction of the Ru alkylidyne **12** and ynamide **13** commences by attack of the lone pair on complex **12** onto the C-atom carrying the oxazolidinone group as the more electrophilic site of **13**, followed by ring closure of the resulting zwitterionic intermediate (**S4**) (Figure S16). This nicely explains the experimentally observed substitution pattern of the resulting metallacyclobutadiene. The hypothesis was tested by a relaxed surface scan of the Ru-C bond distance responsible for the formation of ruthenacyclobutadiene **14**. The low activation barrier corroborates the validity of the proposed mechanism.

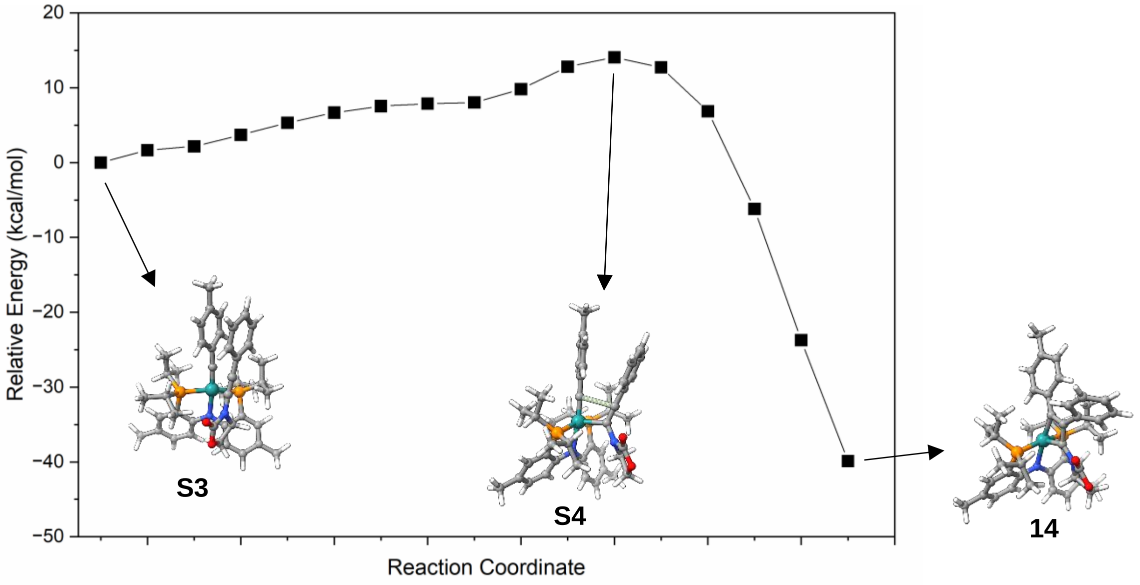

Figure S16. Computed energy diagram for the conversion of Ru alkylidyne **12** and alkyne **13** (**S3**) into ruthenacyclobutadiene **14**, calculated at B3LYP-D4/def2-tzvp/CPCM(toluene) level of theory.

Optimized Geometries

Relative Gibbs free energies were calculated with respect to the combined energy of compounds **12** and **15**. (ΔG = G(**product**) – G(**12**+**15**))

**12**	**15**	**TS1**
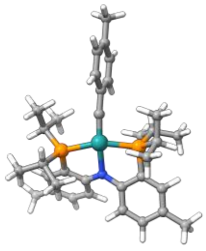	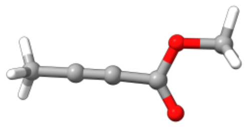	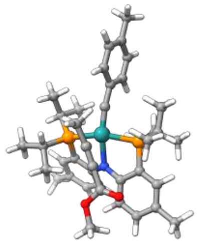
E = -2156.07353952	E = -344.50296034 a.u.	E = -2500.58747058 a.u.
G = -2155.41999293	G = -344.43724920 a.u.	G = -2499.84135996 a.u.
		ΔG = 10.0 kcal/mol
**TS1B**	**19**	**TS2**
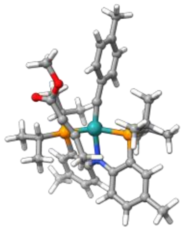	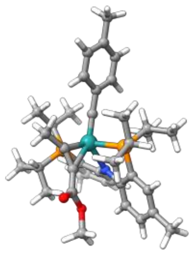	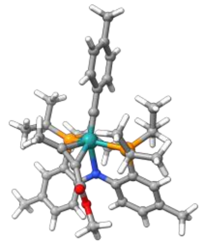
E = -2500.58112537 a.u.	E = -2500.60822528 a.u.	E = -2500.58802034 a.u.
G = -2499.83435249 a.u.	G = -2499.85883673 a.u.	G = -2499.83814061 a.u.
ΔG = 14.4 kcal/mol	ΔG = -1.0 kcal/mol	ΔG = 12.0 kcal/mol
**20**	**21**	**TS3**
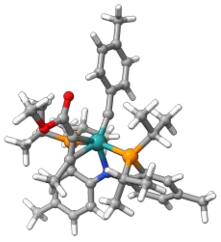	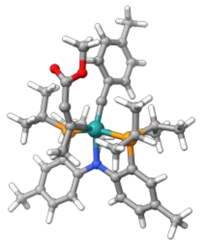	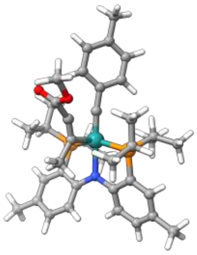
E = -2500.60077514 a.u.	E = -2500.58277792 a.u.	E = -2500.57264634 a.u.
G = -2499.85288723 a.u.	G = -2499.83427360 a.u.	G = -2499.82533874 a.u.
ΔG = 2.7 kcal/mol	ΔG = 14.4 kcal/mol	ΔG = 20.0 kcal/mol
**16**		
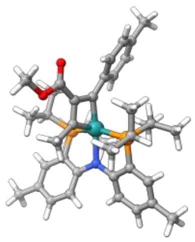		
E = -2500.64968640 a.u.		
G = -2499.89993240 a.u.		
ΔG = -26.8 kcal/mol		

xyz Coordinates of Computed Structures

Structure 12

E = -2156.07353952 G = -2155.41999293

85

Ru	9.09912141113955	6.72882108539214	11.73149811867794
P	10.51454943043611	4.96399746671064	11.13935170426143
P	7.88372851969877	8.20581643930688	13.04179284888260
N	9.59014316123939	5.96263955072393	13.66662338072696
C	8.75007357556693	6.24989198329991	14.73738495517281
C	7.86739602180083	7.34997300779564	14.63954216840727
C	6.99028092415881	7.65806636385630	15.67652447678488
H	6.31998998877488	8.50269680884041	15.57076220496524
C	6.91907060668075	6.89594462431782	16.83719968411131
C	7.75222070530062	5.77627059932744	16.90766153004585
H	7.69461629736317	5.12570379034807	17.77428256413309
C	8.63944377746570	5.45569849047710	15.89566786534937
H	9.23425602108474	4.55881391338345	15.98669046497942
C	5.97959744790958	7.24744471962707	17.95896959407319
H	5.26365538217897	8.00969506344942	17.64773082054559
H	6.52011747471826	7.63756295797411	18.82703996803018
H	5.41632211461000	6.37450051263201	18.29828660074938
C	8.66822020957173	9.86817197078325	13.32538422504544
H	8.67511428597581	10.32481181978549	12.33084239174154
C	7.91023761843424	10.78227916254818	14.28775464015263
H	8.42043437612661	11.74669907159021	14.35864137922676
H	7.87590979451942	10.35408130871095	15.29112807148471
H	6.88684475756863	10.97419049360606	13.96319118738414
C	10.11939840800448	9.67056985753976	13.77083275238604
H	10.66628503370500	9.04218057046973	13.06714500207272
H	10.16438851216287	9.19160333565592	14.75153564057989
H	10.62230214664114	10.63813397757757	13.84574512282793
C	6.10903937924242	8.55434554054295	12.64502401626662
H	5.68574239529174	9.11171714360400	13.48512444632134
C	5.99859064290263	9.40909181558684	11.38029410977820
H	6.44344162578083	8.89541784410471	10.52652344165536
H	6.49146020036923	10.37630673266509	11.48713250075088
H	4.94631471067836	9.59555859741601	11.15162724033605
C	5.34407029184476	7.23807350635021	12.49189729571799
H	4.29338481655850	7.44198027973849	12.26962464506395
H	5.38836877900801	6.63555190978426	13.39965615831517
H	5.76149083754391	6.64760136169568	11.67368076678218
C	10.77897484933132	5.26301223214771	13.83718102604019
C	11.37445040545091	4.65650916027313	12.70657397479862
C	12.57713276785100	3.96563690931847	12.81495917097896
H	13.01265141404082	3.51584360955685	11.93063381968275
C	13.26406240313651	3.85911336578514	14.02048706250033
C	12.70266572649815	4.50536153555831	15.12368099996696
H	13.22852502680724	4.48849926898813	16.07278254310879
C	11.50046611807819	5.18764190377910	15.04386412952291
H	11.13167835831594	5.69758535172052	15.92137312053358
C	14.55558027187139	3.09444045643990	14.12975003224708
H	14.97402088386921	2.88585075034994	13.14380623699698
H	14.41178873661452	2.13433973130108	14.63548757930745
H	15.30238036515152	3.64962859985029	14.70258527589126
C	11.84566406407903	5.24838624016408	9.88467890826913
H	12.51615848479587	4.38565153336077	9.92619598142691

C	12.64337213742273	6.50529470189414	10.23725566140607
H	13.45269422662905	6.64400726774643	9.51567293577996
H	13.08301274714384	6.43895945967467	11.23284269837243
H	12.00318679052112	7.38774020143862	10.20965691053620
C	11.25135757025738	5.33966269601781	8.47776663454292
H	12.04547755619173	5.54130939773264	7.75456435710340
H	10.52485019841780	6.15083773387306	8.41216996711138
H	10.75575124553501	4.41549153730707	8.17785979555315
C	9.71386982172467	3.35167021474997	10.66617586725679
H	9.20416435090610	3.57862614699084	9.72497395692323
C	10.68368452654805	2.19174465087919	10.44234933493561
H	10.12947433713215	1.30411384076305	10.12543633087013
H	11.21245189598340	1.93895035700470	11.36301096683356
H	11.42472011315647	2.40958940087249	9.67211350178366
C	8.65164671730037	2.99653972482979	11.71063831762101
H	7.92702729029123	3.80306600888653	11.83043498836286
H	9.10827496269406	2.80656145017626	12.68448555609752
H	8.11786828159001	2.09234687201623	11.40735530091324
C	9.04352984791685	7.66155577219868	10.29114618452770
C	9.20123427321036	8.62683236092249	9.24669591839101
C	10.06609525084371	9.71930703608916	9.41116234598850
H	10.61799815091651	9.81627683382730	10.33718203698549
C	10.20681262401127	10.66017113943663	8.40525522593216
H	10.87437877929958	11.50181132207490	8.54948349108840
C	9.50154347523934	10.54344429225373	7.20399888315412
C	8.63969724367642	9.45450422603544	7.04381788037842
H	8.07903508452889	9.35163650428327	6.12189832312479
C	8.48723045879959	8.51030794092701	8.04501560116895
H	7.81313092139183	7.67343820258124	7.91322338142584
C	9.68392391040261	11.54811659944975	6.10150153165832
H	10.44425932360932	11.20804101975513	5.39143911466740
H	10.00943397726835	12.51278598083278	6.49262232928343
H	8.75934042449049	11.69453981066734	5.54096425916360

Structure 15

E = -344.50296034 a.u.   G = -344.43724920 a.u.

13

C	-4.83998069810880	-0.88359196086576	0.24962335905857
C	-3.79530819251043	-0.28837600987153	0.29483302853904
C	-2.54862250354982	0.42003725022965	0.42209740974250
C	-6.09864781681252	-1.60227512032481	0.19201729545527
O	-2.01440145948202	0.67467442764345	1.47754829404850
O	-2.05384778555690	0.76374095476955	-0.77677185644744
C	-0.80381428224309	1.48170933697522	-0.75724175621547
H	-6.77726618462408	-1.13493694952969	-0.52461372932136
H	-5.93158073592344	-2.63433767125860	-0.12525589051104
H	-6.58371947843255	-1.61936839402451	1.16997661077464
H	-0.56426744421018	1.66957020697773	-1.79972145998374
H	-0.91516570318261	2.41875621540664	-0.21319246239172
H	-0.02883479236354	0.87762077687266	-0.28723390574776

Structure TS1

E = -2500.58747058 a.u. G = -2499.84135996 a.u.

98

Ru	8.49474559731239	6.10600566828599	11.83980120854874
P	10.14435529861481	4.52137053988991	11.31114650310755
P	7.53966953648388	7.79899659020283	13.12015426048123
N	9.20528420056531	5.58934487585442	13.81332513061131
C	8.44473502521082	5.97368905822856	14.91285771010719
C	7.53654118870868	7.04430977636831	14.76739220446728
C	6.72169377299545	7.44039976084188	15.82156608227123
H	6.02975723288224	8.26227348650894	15.68149411389446
C	6.75163947795793	6.79771437874642	17.05461440876813
C	7.64107748104212	5.73076857291708	17.19263879513040
H	7.67966067203423	5.19204701480026	18.13381191006080
C	8.46208105110481	5.32030556698650	16.15633605829458
H	9.10508121295921	4.46376408114604	16.29858627785322
C	5.82740802499219	7.19688536738602	18.17233523003466
H	5.42379814549737	8.19791295437793	18.01178158000093
H	6.33922712832996	7.18896754515359	19.13735914435262
H	4.97876412404546	6.50985705332432	18.24966909122643
C	8.63771450313824	9.29966157746328	13.28429611826686
H	8.63542493766853	9.73974619102332	12.28291354317370
C	8.13039188951541	10.33732576282807	14.28630373125039
H	8.82345414632264	11.18198019603044	14.32517844262129
H	8.06917969616702	9.91388584427456	15.29070206031979
H	7.14822640002270	10.73009576041036	14.02211954655527
C	10.06953549811651	8.87063828714418	13.61309638906221
H	10.44689093248901	8.15659686437797	12.88157901331772
H	10.12045970543326	8.39718037602061	14.59547534613837
H	10.72397404204029	9.74607319248796	13.62754908831809
C	5.84922899280551	8.50730314000161	12.85125377594764
H	5.63624135355799	9.15230697481660	13.70797375537481
C	5.82119621827375	9.36189430556341	11.58065628569552
H	6.06697794209609	8.76185348527577	10.70328317914007
H	6.51842320380834	10.19905235113631	11.62176991303625
H	4.81811170302871	9.77099197978491	11.43692706659671
C	4.78699027768683	7.41025128858134	12.79185797754850
H	7.00267195145502	1.73539636071950	16.36579706963960
H	6.41508002039332	3.36536640086516	16.80679958627365
H	5.26188961392600	2.12865415400889	16.25108927114339
C	10.49739924229448	5.12065918628403	13.95358859779795
C	11.13144225725944	4.53765774307679	12.82999695710684
C	12.44185111376911	4.07451012274857	12.90616152496998
H	12.90368035947527	3.64122913975889	12.02699405957928
C	13.20154799874941	4.18268326448119	14.06612324689784
C	12.59241137991916	4.80384470680510	15.15924435901352
H	13.16236843841884	4.95063665577558	16.07114401182789
C	11.28722017940701	5.26057300338110	15.11405257341757
H	10.88362917172972	5.76329358914902	15.97991299907007
C	14.61447127842967	3.66947680200632	14.13891544897662
H	15.00324076470992	3.44591370715429	13.14402867368365
H	14.67960064610353	2.75100972555448	14.73103929823243
H	15.28236755620833	4.39845671310454	14.60484089607237
C	11.32734157706408	4.94487232325802	9.94696629471001
H	12.12441721200389	4.19807254160213	9.98363018235463
C	11.95032714368208	6.32321528266577	10.16595993497083
H	12.66163461050940	6.53864058339218	9.36406403334613
H	12.48143573696908	6.37788370719821	11.11632499161397
H	11.18589056242284	7.09879309203554	10.16354084970463

C	10.63505069452959	4.84941448085901	8.58604115486997
H	11.34642267951735	5.08528315123910	7.79048893838543
H	9.80952424289677	5.55801813960177	8.51648318830326
H	10.23947046224844	3.85116099471806	8.39257470206113
C	9.71975809780121	2.72116132056023	11.02726378776176
H	8.97028064279367	2.74581151887520	10.23032394800096
C	10.87954720143894	1.82985329622947	10.57880432276313
H	10.51676082109372	0.80816010707580	10.43545283037664
H	11.66549181911945	1.79235521715691	11.33511580660200
H	11.32458385822783	2.15439366270090	9.63838480518736
C	9.06554310596358	2.15559346919732	12.29040446007515
H	8.25347689692102	2.78259191455968	12.64892804014289
H	8.66140999987408	1.16071431093350	12.08661754066496
H	9.79888980836094	2.06240877740149	13.09358842274824
C	8.36328670677373	6.96755617388653	10.36268601372672
C	8.41005597172883	7.89148222064470	9.27001454942957
C	9.22283866282866	9.03410655939756	9.31998425140015
H	9.83194864888953	9.20787950335116	10.19711262978736
C	9.24140683108200	9.92912298613694	8.26464831756572
H	9.86949531604831	10.81060636804994	8.32250449602328
C	8.46391406318033	9.71553336386805	7.12229684908539
C	7.65638759624337	8.57615375024396	7.07429523026906
H	7.04154490467310	8.39728331806460	6.19987648854216
C	7.62538494697242	7.67757844843779	8.12745463367981
H	6.99069210304013	6.80200561756222	8.08065363141257
C	8.51696838093570	10.67196639542289	5.96464922278823
H	9.31598781397223	10.39277475055323	5.27081105245305
H	8.71814938634841	11.69042026108345	6.29968921470492
H	7.58152981673696	10.66888119433724	5.40375215857152
C	6.13460100834618	4.27901696521106	11.71919538768846
C	6.00384205741152	4.10990479881310	12.90863038392908
C	5.75772632732161	3.88831941606251	14.29963859438732
C	6.03403909477263	4.28518192795053	10.26793283513938
O	4.93302465376203	4.48522315107607	14.95951915472248
O	6.52359208710550	2.89084062709623	14.78100187272636
C	6.27652187450220	2.51313575869750	16.14579883783679
H	6.97767870280424	4.00106701878054	9.80547135290092
H	5.25787420765977	3.57652957873195	9.96139400491980
H	5.77044814617985	5.27716800270762	9.90251145119759
H	4.93417066355855	6.78124274485476	11.91403016652932
H	4.80232509339441	6.76471130870623	13.66863707396370
H	3.79594917513191	7.86641675589680	12.71997639469684

Structure TS1B

E = -2500.58112537 a.u.   G = -2499.83435249 a.u.

98

Ru	-0.22473407586024	-0.12410468262744	-0.03343338966955
P	1.23681465271916	-1.84937645017071	-0.74971832356555
P	-0.87227798227609	1.60612858354570	1.37777344864720
N	0.48155813788538	-0.84361535573653	1.86733664529157
C	-0.18830340554075	-0.43760408775721	3.01903998163399
C	-0.92635828563081	0.76357244049105	2.98157100105785
C	-1.66407318984966	1.18526360843334	4.08351237368702
H	-2.22146316619295	2.11273562711650	4.02899981683257
C	-1.72702167672186	0.43674566781146	5.25464863925196
C	-1.02206267080841	-0.76959794454113	5.27689703810750

H	-1.07476466760203	-1.39834050531720	6.15953716305460
C	-0.27093157459298	-1.20009689180010	4.19652296502707
H	0.23170501059380	-2.15547294229166	4.24836662349955
C	-2.53461979981540	0.89145520022105	6.44044000985290
H	-3.05163533153804	1.82816362889055	6.22785783623297
H	-1.90152571856383	1.05168039203175	7.31770538260755
H	-3.28743661429080	0.14934628011529	6.71998185144893
C	0.44697553813709	2.91478494145279	1.55084178811295
H	0.46808288970611	3.40335056032048	0.57227575143864
C	0.14288790100539	3.95832093687208	2.62623209124966
H	0.95962911005830	4.68305042411075	2.67567084043247
H	0.05509958858940	3.49174710423202	3.60945410392675
H	-0.77557458012679	4.51155033620233	2.42809917574944
C	1.81271931853008	2.26513885113404	1.78646278709237
H	2.05412406619521	1.54784084790185	1.00292745983523
H	1.83929012672651	1.73672434128516	2.74139218224633
H	2.58696960691841	3.03627807795184	1.80842392773918
C	-2.44647044052090	2.57417812421433	1.22264608018032
H	-2.52543536923266	3.19520761627656	2.11907563993804
C	-2.40036990650945	3.49330130151124	-0.00131558627934
H	-2.32316456169402	2.91509186738761	-0.92174105943760
H	-1.56639559375949	4.19467350874321	0.02875696255894
H	-3.32263755315718	4.07715378317462	-0.05067345596302
C	-3.66627152997142	1.65548564837042	1.15826563745108
H	-3.77104290273206	1.05391184257768	2.06086136008594
H	-3.60470321315010	0.98440314120576	0.30011724388664
H	-4.57250900412042	2.25709534098909	1.05269524526412
C	1.69327255009047	-1.51212406274836	1.93396256390193
C	2.21637774393193	-2.09542496187762	0.75611397388133
C	3.44109256609395	-2.75686136965486	0.76995978663242
H	3.82058954561712	-3.18628794274587	-0.14922938334001
C	4.21948387151360	-2.85611120780421	1.91813692373840
C	3.72676467620687	-2.23240547060384	3.06666149187208
H	4.32149554662235	-2.24472546312901	3.97426402195042
C	2.50793472777139	-1.57758760184797	3.08252005615684
H	2.19497774040278	-1.08479213720106	3.99060975111801
C	5.53344488400862	-3.58941421080365	1.92215940918735
H	5.88773753938156	-3.76702562508596	0.90549955165021
H	5.44811185339464	-4.56324835982346	2.41481435157961
H	6.30422810174938	-3.02718200068544	2.45494030820033
C	2.46401537695218	-1.36916787208211	-2.05442310407528
H	3.18470182271330	-2.18873122129738	-2.11647850480455
C	3.21688531819518	-0.09987036951437	-1.65556206208448
H	3.93739209077751	0.16221191169339	-2.43483811885769
H	3.76015188392781	-0.23066424860766	-0.71950078547872
H	2.52885390430706	0.73616406562950	-1.53070922001924
C	1.77732228553870	-1.21541289363843	-3.41277829362291
H	2.52135049704394	-0.97126421868857	-4.17537922554134
H	1.04366152667088	-0.41023215760459	-3.39065317958573
H	1.26145585778156	-2.12333221542453	-3.72507225190626
C	0.62385858075199	-3.53739485506638	-1.25384164639373
H	-0.14330185992873	-3.32159419814021	-2.00055873096790
C	1.67576741625571	-4.45935810782956	-1.87484821779230
H	1.19819138135701	-5.39960163885335	-2.16365269551890
H	2.46737974385866	-4.70179544867387	-1.16337154399815
H	2.13400544280261	-4.03925350506767	-2.76955121705668
C	-0.03992982643198	-4.22451537509206	-0.05890006598445
H	-0.79332880714112	-3.59563282928977	0.40621378854451
H	-0.52979741410891	-5.14293467741352	-0.39111601802147
H	0.69981415995352	-4.48964937463782	0.69901564572718

C	-0.42662268991245	0.79542497096217	-1.46969227585936
C	-0.43644509348502	1.74063211590815	-2.53890835866608
C	0.57700623997900	2.70409156047108	-2.66079728900723
H	1.37579099310504	2.72599103656967	-1.93152951393144
C	0.54969980610023	3.61793150439416	-3.69866598111299
H	1.33686658883735	4.35860900952606	-3.77925014740777
C	-0.47869875979155	3.60376540375554	-4.64692039774055
C	-1.49007055269905	2.64893434699774	-4.51472852822889
H	-2.30057061434744	2.63044147322486	-5.23383420380936
C	-1.47580377154597	1.72919389994622	-3.48011984594773
H	-2.26885301555303	1.00191321928641	-3.37862752943363
C	-0.47968461077140	4.57589692398363	-5.79216487089238
H	-0.07231279997933	5.54286416494941	-5.49284710357568
H	-1.48604368729993	4.72806926199813	-6.18342355026357
H	0.13990847308222	4.20250013852046	-6.61340216701007
C	-2.34171224915914	-1.58049012034755	-0.24746099508561
C	-2.63027684854787	-1.81868587296930	-1.42148162052413
C	-2.56819106339677	-1.86840101149077	-2.82825397909980
C	-2.62887697860801	-1.81888047971163	1.17974364584729
O	-1.80133037014289	-2.55820572788817	-3.48554921002406
O	-3.48758648181454	-1.05093678316813	-3.41565657322211
C	-3.52034444126382	-1.08310372594505	-4.84976725412201
H	-4.31035520762621	-0.39389727977727	-5.13796950056984
H	-3.74526381921975	-2.08816680276151	-5.20653559173663
H	-2.56581249817068	-0.76157367691910	-5.26738245822243
H	-1.76316785794861	-2.20896979015296	1.71111852393642
H	-2.93703380251700	-0.90997167967916	1.69003430092195
H	-3.44224874817135	-2.54592560040149	1.25215787719065

Structure 19

E = -2500.60822528 a.u. G = -2499.85883673 a.u.

98

Ru	8.45656485702609	6.00616107545782	11.85825693536382
P	10.05651314815572	4.32492222348784	11.41268947508019
P	7.34363642572524	7.66316943947370	13.13090486575234
N	9.42022717828832	5.79056338190020	13.85813238216077
C	8.68424286852761	6.14444400483864	14.96631227668278
C	7.59375663985726	7.03430891083648	14.80656273355046
C	6.78193856556510	7.37593856266781	15.88531980674610
H	5.95436022906251	8.05850819647142	15.73206695659002
C	6.98190118494542	6.85054299402057	17.15601487011476
C	8.04221922898554	5.95141003975020	17.30748806527400
H	8.21220678934609	5.49084042132546	18.27548439563641
C	8.87176516029818	5.60862261558293	16.25691055944123
H	9.64667438611333	4.87471107372146	16.42144295679428
C	6.08921540381837	7.21285561820814	18.31231039960643
H	5.31170655006113	7.91259949039835	18.00223280239505
H	6.65321869948264	7.68020323488703	19.12474367748723
H	5.59588371747594	6.33117324277482	18.73174369388278
C	8.21321720235955	9.31678924814255	13.11273238465100
H	8.15359025113379	9.64135541304880	12.07020672036746
C	7.56534753559519	10.37964543604183	14.00145795102588
H	8.15643921132263	11.29835258350017	13.96096227943745
H	7.53204205976662	10.05590995001246	15.04388207423145
H	6.55192117023582	10.62904297597797	13.68741259638083
C	9.69091069593889	9.14478596549390	13.47451189931981
H	10.17816099798551	8.39892715681249	12.84897849918677
H	9.80296045563091	8.83585800428076	14.51532618771401

H	10.21068943799084	10.09813650716781	13.34976514294213
C	5.55830296347717	8.11065582518693	12.92441819862162
H	5.35709231279630	8.88774475434961	13.66597601920200
C	5.35106717642998	8.70576115169018	11.52707988564823
H	5.58533746857906	7.96819064004064	10.75736180025551
H	5.97177799810363	9.58476616733015	11.34953896044564
H	4.30711715910727	9.00340515389132	11.40368390220300
C	4.59004159655330	6.95458798159895	13.17436569507134
H	6.57270957891972	2.96472227260511	16.45976684235646
H	5.00417422130362	3.59403225597304	15.88356643796127
H	5.58403253027077	2.01602088781370	15.31013798071998
C	10.69650982194562	5.28599500797539	13.90616298664825
C	11.18852293994085	4.53790555681261	12.80738039846172
C	12.47991224869479	4.01276966017447	12.81761511286962
H	12.82497300257418	3.43926720630069	11.96491135260830
C	13.35680281458786	4.22030786599186	13.87419998767136
C	12.89087344771608	5.00776814379495	14.93357750967606
H	13.55836788757201	5.23308777760065	15.75917140370045
C	11.61095350755137	5.52625988792491	14.95713858399075
H	11.31455521018287	6.15424912999727	15.78433887297007
C	14.74813909327832	3.64692114388061	13.87633688772815
H	14.97881392412223	3.17345900722044	12.92074266092752
H	14.87026331744763	2.89096224746735	14.65808066406827
H	15.50092664220514	4.41892377800012	14.05808019083961
C	11.07921981672121	4.49419343283831	9.87513862453857
H	11.77679254681270	3.65307340161587	9.88125061711631
C	11.89167814468195	5.78786224637812	9.85833784158757
H	12.48883294162642	5.83090598590992	8.94370214096619
H	12.56713477555494	5.85246963734545	10.71061602376870
H	11.24044787329699	6.66022834273965	9.87742552676331
C	10.18226736955817	4.38184298693643	8.63963228438955
H	10.79087639229848	4.41851908111369	7.73289781580374
H	9.47253151563833	5.20915923475342	8.60534952461233
H	9.61368981205695	3.45067194232571	8.62198882849677
C	9.51330899013743	2.53906533552893	11.44816906079502
H	8.58828127506782	2.53392790673165	10.86444359008858
C	10.49393225529157	1.53855130276965	10.83393625528726
H	10.08376867930217	0.52939171628614	10.92811036400684
H	11.45257360302667	1.54943408584481	11.35695550881218
H	10.67877165007214	1.71961102964866	9.77548260780334
C	9.17546221630807	2.14021323205613	12.88759910160574
H	8.55213346608610	2.87662643992546	13.38764769187990
H	8.64239272838752	1.18612612998813	12.88886960259655
H	10.08804164190388	2.01875883155442	13.47405682555496
C	8.82159827739080	7.13997836079512	10.59963645011163
C	8.84522998485378	8.13384887355364	9.57290931533613
C	9.75414041486941	9.20147122799806	9.62452870781597
H	10.44754660895947	9.26355174766003	10.45278914664849
C	9.75873217938367	10.16646663714021	8.63170293482183
H	10.46031902234859	10.99024303844582	8.68907457241415
C	8.87317489016447	10.09496236930559	7.55302934581134
C	7.97105911174837	9.02646706591552	7.50129441173479
H	7.27373106053808	8.95944855870809	6.67429427951386
C	7.95254466502450	8.06143380035135	8.49111175858285
H	7.24835703370960	7.24127083037735	8.44706923033196
C	8.90278890563158	11.12389907989691	6.45921134487410
H	9.46054331792482	10.74958876179386	5.59531900334547
H	9.38539983704182	12.04358996920323	6.79044510669896
H	7.89573442703303	11.36355093143259	6.11369573583520
C	6.84124088002842	4.84595494670229	11.19975478630028

C	6.75833694999543	4.56372716849917	12.44175905933271
C	5.96157391033675	3.86623233086755	13.40619910309381
C	6.28567599472420	4.55475666606682	9.86406141059227
O	4.80209340500604	3.51323025559931	13.25110311101756
O	6.61968168147135	3.66889909382948	14.57281798722737
C	5.88995644175303	3.01743540679795	15.61559833094199
H	7.06616514907447	4.21891904465825	9.17778601172442
H	5.50210626149732	3.79424719115098	9.91855931139698
H	5.85859022740069	5.46506187414252	9.43380679560173
H	4.66736991176023	6.19446719936032	12.40058982945158
H	4.76228588023200	6.47085365837149	14.13468416035407
H	3.56676248520762	7.33958556017855	13.17073028014469

Structure TS2

E = -2500.58802034 a.u. G = -2499.83814061 a.u.

98

Ru	-0.59921914929056	-0.09700678848824	-0.29829136091083
P	-1.13698984922375	1.81127855442636	0.88298255963829
P	1.17608171511369	-1.73332643096706	-0.64218330819390
C	-1.70914562310770	0.22345561115491	-2.08226027158860
N	1.15483501405502	1.25696476972360	-0.60961807970370
C	-0.56730874436402	0.08818913837263	-2.59455411137080
C	0.26946815025559	0.06154045445828	-3.75315067770602
C	-3.13398387479554	0.43158070543878	-2.41641332353808
C	0.85279488574263	2.59513120051534	-0.75435206620327
C	-0.27086205386536	3.09093964562865	-0.05379952510912
C	-0.65128580817152	4.42528095708870	-0.14734516992859
H	-1.50879402277195	4.77869490744361	0.41272257949421
C	0.03157238805088	5.32399055341928	-0.96140073176326
C	1.10730755988703	4.82112120334550	-1.69892416590236
H	1.63364947437652	5.47996947922714	-2.38205496279512
C	1.51481386837867	3.50162817710789	-1.60412114409669
H	2.32612484336898	3.15419698766736	-2.22489251594693
C	-0.37421313824622	6.77029809615966	-1.05505569991062
H	0.36034055363012	7.42539193503158	-0.57634669949612
H	-1.33506592738862	6.94159098063252	-0.56721971081880
H	-0.46258596282165	7.09517908048824	-2.09498916085702
C	-0.35751945896430	1.84462819758475	2.58790357571594
H	-0.84076939798824	1.01585889617834	3.11358760618744
C	-0.60782064301487	3.14546252826711	3.35620678495487
H	-0.24803290193378	4.00931650215877	2.79271071885867
H	-0.05899831704670	3.11919721632085	4.30107956400870
H	-1.65880878892399	3.30522961749023	3.59426978837472
C	1.14278460844094	1.55676742237938	2.52444819144679
H	1.35761026460873	0.63701065592554	1.99013586299657
H	1.53755054076626	1.47066539066312	3.54016573538875
H	1.67587939539902	2.36736036750076	2.02510373919469
C	-2.84008144926233	2.45499599922656	1.24189398600539
H	-2.67563506141753	3.40125148038401	1.76206852462218
C	-3.67597737435111	2.74207944924714	-0.00277019466961
H	-4.05882396747889	1.81830414464468	-0.42779024208387
H	-3.11577691212487	3.26999863124154	-0.77377226067637
H	-4.53467649743866	3.35943309724335	0.27314477959111
C	-3.59110601765033	1.50657164836776	2.18093670000857
H	-3.73466009734561	0.53174115984330	1.71244550325476
H	-4.57818511206314	1.91837483905392	2.40541010460820
H	-3.07360695332483	1.35169744720985	3.12742970136074
C	2.41622452088056	0.75196457205629	-0.71265773872526

C	2.62116224285094	-0.65269071886693	-0.78477227026789
C	3.91584899310373	-1.17684104222657	-0.85551397382876
H	4.04852413639713	-2.25048177009646	-0.88829483488144
C	5.05242742160016	-0.38432073212289	-0.85157543744104
C	4.85116255203777	0.99583443632466	-0.72296573125585
H	5.71120860914752	1.65499626104493	-0.65820318607226
C	3.59032803010234	1.54609403365438	-0.64973648884082
H	3.49965619282886	2.61128222192915	-0.50419250565784
C	6.43386972004877	-0.97189987104822	-0.95339437727588
H	6.91080921141232	-0.71912268844661	-1.90570281439993
H	6.40368262830906	-2.06031713507650	-0.87999751241936
H	7.08880770852422	-0.60028437000949	-0.16041935027271
C	1.08920313253500	-2.77679607809181	-2.19069895271977
H	1.26435531342644	-2.04173626315232	-2.97563349760202
C	2.15985962157105	-3.86033889582137	-2.34137701504685
H	3.16766494844998	-3.44878615867814	-2.32487318284109
H	2.02720408448141	-4.35929545452917	-3.30514069029046
H	2.08919022821907	-4.62533157655674	-1.56709798353211
C	-0.31837915103297	-3.34547378338859	-2.40225868145950
H	-0.56185910678837	-4.11395446095735	-1.66779335161556
H	-0.37975827834760	-3.80066327402789	-3.39377690955759
H	-1.07336193691562	-2.56424787740611	-2.34155562660097
C	1.65963813435249	-2.91186258189507	0.72114431830895
H	2.61568194996529	-3.34394985853656	0.41176387013477
C	0.65821437387527	-4.05111791287450	0.91980797168836
H	-0.33933827431151	-3.67399508467825	1.13854940010711
H	0.97219531859821	-4.66639366321064	1.76732685599500
H	0.58950467230845	-4.70203300741582	0.05028245798280
C	1.88314817954342	-2.14941142415061	2.02628482839382
H	2.63923576864052	-1.37228117277227	1.91679442885598
H	2.21578094452180	-2.84031767114511	2.80534807332191
H	0.95877138701977	-1.67767134194323	2.36232774572356
C	-1.52847313057628	-1.13457411571857	0.71608357639185
C	-2.29961857500371	-2.01700454545932	1.52797526355679
C	-3.26152203798072	-2.85692921312391	0.94143197025148
H	-3.40102505733977	-2.82497981053037	-0.13137619965759
C	-4.00785622484550	-3.71813776355375	1.72357100206665
H	-4.74099822940878	-4.36590708080708	1.25694542260045
C	-3.83404921560417	-3.76624103115758	3.11170374737461
C	-2.88120136399340	-2.92567251163279	3.69223484336924
H	-2.73225998271169	-2.95321229707762	4.76511939180044
C	-2.12013083577299	-2.06655285133494	2.91760859531978
H	-1.37653543902450	-1.42783321324293	3.37550272135503
C	-4.66278663096383	-4.69653335443309	3.95075871164043
H	-5.71493378086482	-4.39864348335905	3.93593221482831
H	-4.32853937315894	-4.70131706400705	4.98802160316839
H	-4.61392810757257	-5.71852549454531	3.56808942629822
O	0.06029118912126	-0.61759220767633	-4.74672552964181
H	3.10343454785755	1.47785850967954	-4.41547271682386
O	1.35799558127879	0.84904886787758	-3.62903991524843
H	2.67357835400943	-0.19802732495726	-4.85792214563461
C	2.29513968945666	0.81386176462541	-4.71100122132880
H	1.83505618620391	1.16254402653290	-5.63583027628980
H	-3.34113641283729	-0.00703493143227	-3.39581983334449
H	-3.79479178082822	-0.02494572200016	-1.68065958190147
H	-3.37075125949398	1.49457166864410	-2.46590156649897

Structure 20

E = -2500.60077514 a.u. G = -2499.85288723 a.u.

98

Ru	7.92981088448769	12.27603858863646	12.00743945088078
P	6.86291494400026	14.18418324018205	12.87954298523559
P	9.69255075668845	10.91498522968936	11.22459105778468
C	7.39451205122801	12.94580740552397	9.99646189995316
N	9.60235233454296	13.60932516523875	12.24800314321891
C	6.68339177939665	11.94151327722481	10.37558399107033
C	5.59347241363652	11.12424528292177	9.91333916492743
C	7.56635424931341	13.89883242498079	8.88324692345810
C	9.37071160619258	14.96587802404581	12.15934615320351
C	8.06237264760920	15.45198683152183	12.40481468156634
C	7.76620894388433	16.80704780836690	12.28542051061559
H	6.75710920946347	17.14872197357671	12.48446498657696
C	8.72107832566893	17.74011482547574	11.89697777863675
C	10.00406468769660	17.25525410220432	11.62414581319779
H	10.77061609767321	17.94562066330553	11.28719175722367
C	10.32834496042664	15.91667852507637	11.75127823607394
H	11.32731943710994	15.59228423479398	11.49848036857785
C	8.38883392268022	19.20128677938022	11.75558857164911
H	8.99812839944079	19.81816727152575	12.42234624627351
H	7.34098156087636	19.38909500392171	11.99420165231368
H	8.56833107248414	19.55764787191562	10.73715616851126
C	6.82621856013215	14.25397994712442	14.74672426037931
H	6.21050749901632	13.39804992896265	15.03644208339837
C	6.20637038464542	15.52988471279379	15.31934132328068
H	6.74471853318724	16.41752346551274	14.98023864799526
H	6.26858558424351	15.50526079523563	16.41038385894733
H	5.15533974480697	15.64414208635210	15.05480633846767
C	8.23287342729374	14.04570212001261	15.31525038059669
H	8.69790928978994	13.14051975718152	14.92999268669216
H	8.17723213386670	13.97165498701759	16.40414294984230
H	8.87948280562298	14.88982614470308	15.06858426832820
C	5.16803585189619	14.74821808482025	12.38685512488122
H	4.97761167488741	15.66261336798082	12.95398328418826
C	5.02082416476443	15.08077787402518	10.90118052555332
H	5.07672070393100	14.18367035856345	10.28932858713419
H	5.78535755250557	15.77599338601833	10.55688384708580
H	4.04484568598990	15.54442193058060	10.73483217500316
C	4.15091370959254	13.68428472020036	12.81638055805052
H	4.33296991808941	12.74335963299744	12.29525151016325
H	3.14008611133402	14.01851474356432	12.57055731931506
H	4.18409095624255	13.48533748444083	13.88829022412011
C	10.84986024184404	13.05839461942911	12.45368896369101
C	11.08885318531453	11.72577179022658	12.04030814949676
C	12.32958832501072	11.12533487060123	12.23775823456695
H	12.48480609171972	10.10744344736973	11.89790689687877
C	13.37454794611635	11.78136283209813	12.87765277909795
C	13.12216523493979	13.08145472713867	13.32872275750532
H	13.89913007755679	13.61589264314609	13.86582483804705
C	11.90568469898849	13.70716696129593	13.12778966449981
H	11.75488070706432	14.70254080261667	13.51995789010066
C	14.70723877304298	11.11899474639895	13.10230066343901
H	15.51924570277764	11.68078783525427	12.63195577477856
H	14.71722836801068	10.10979142265965	12.68802777267236
H	14.94506424939112	11.04435069843309	14.16743657789254
C	10.10128322524473	10.96210294531273	9.40166585316852

H	9.12691171790726	10.89084212856926	8.91017638825971
C	10.72316501389300	12.32080676807754	9.05738354689338
H	10.17493942638343	13.15112284118424	9.49761485088757
H	10.73836095625857	12.45563507950924	7.97317999824270
H	11.75094477367088	12.37239412783266	9.41940398783528
C	10.99863832750704	9.82987658706824	8.89958102537474
H	11.96123327708817	9.82755943527505	9.41611619175964
H	11.20055754815298	9.97313262788237	7.83452639195929
H	10.54427735038219	8.84672806118995	9.01785789243008
C	9.72357220742286	9.12444283480043	11.69283339668614
H	10.63028096195242	8.70217394931849	11.25209378234039
C	8.50149078986064	8.42925845984462	11.08271740304170
H	7.58044933318307	8.81934844964669	11.51697762812966
H	8.54633953093337	7.35581686195968	11.28172378417198
H	8.44148738091200	8.56673090854786	10.00182251016705
C	9.78410345900429	8.91429511966004	13.20421784247448
H	10.66842964876494	9.37267673514257	13.64582420323914
H	9.81047308989647	7.84296190950011	13.42028698801345
H	8.90684026030322	9.33239186086971	13.69438741607090
C	7.32003614767991	11.28092372590752	13.30917825520940
C	6.50781271473485	10.55371436546659	14.22923079824409
C	5.33588480510983	9.90867072940732	13.79836080269709
H	5.06851878142758	9.96244088031663	12.75077032244778
C	4.55512003195710	9.21481980608638	14.70531669150522
H	3.65592426889817	8.71527454517019	14.36328653953594
C	4.90522435084528	9.14259236001795	16.05786753592755
C	6.07524756018506	9.78129195692686	16.48000582932108
H	6.36452066636457	9.72635801322165	17.52291685834870
C	6.87000433034283	10.47403906301471	15.58380101329782
H	7.77825704400681	10.95953603089154	15.91612172068709
C	4.03126152586977	8.41199012275380	17.03688858863230
H	3.21338414876469	9.05539830810845	17.37607709928080
H	4.59312985171150	8.10237319366544	17.91859891349284
H	3.58011774039487	7.52846171796095	16.58259674359983
O	5.01380831155106	10.24943841055214	10.53400705615932
H	3.23797623018423	10.84937991946278	8.67428373827252
O	5.23749797416098	11.44356673009768	8.63627209129051
H	4.00484957589349	11.08626946893015	7.07766627896301
C	4.14323988536249	10.70052130468067	8.08486717345251
H	4.37566367213366	9.63567167596141	8.05337000311423
H	6.64912268974229	14.00688684122186	8.29673449476694
H	7.88220208559806	14.88157110686993	9.23931273737800
H	8.34662215024312	13.54403257785071	8.20467017619013

Structure 21

E = -2500.58277792 a.u. G = -2499.83427360 a.u.

98

Ru	-0.22247064071473	-0.21110666260397	-0.00770473372670
P	1.55064460805448	-1.56012713088048	-0.74596155724417
P	-1.39632219517484	1.34011219671587	1.33330199736088
N	0.71722780991055	-0.62491947506833	1.86664220085966
C	0.49245875186168	0.20621122943761	2.95614051883925
C	-0.61067542852892	1.09300167285458	2.93953064294946
C	-0.93186883287847	1.85514798606525	4.06259798240087
H	-1.81234612179313	2.48521660395046	4.03734560671837
C	-0.14915232820063	1.85300474285126	5.20927320613103

C	1.01967970032601	1.08628998169286	5.16805450849092
H	1.70273047544317	1.11208679088523	6.01068016608017
C	1.33707907851658	0.29135173887565	4.08306492587212
H	2.26087993771752	-0.26655692965309	4.10142470514909
C	-0.52137970180890	2.65547944952374	6.42585107174502
H	-1.31930245103246	3.36442810984710	6.20008437349657
H	0.33288813857827	3.21828422575731	6.81016088932074
H	-0.87266855038341	2.01113531936763	7.23764948012851
C	-0.93895269264311	3.08925064126306	0.85219383450853
H	-1.21278680409593	3.13695782757052	-0.20560449869903
C	-1.67605537608316	4.20499418803989	1.59420639665604
H	-1.35855912286692	5.17084059912648	1.19204292037800
H	-1.43793607295126	4.20190966998372	2.65832201633123
H	-2.75788603324517	4.14418267697650	1.48268812974472
C	0.57416365510733	3.28112373937071	0.97194591439207
H	1.12343477025935	2.47806210406661	0.48251867917321
H	0.88223847115106	3.30402323804931	2.01832688051993
H	0.86670303964712	4.22767930961798	0.51127823418950
C	-3.22603083326166	1.39384569936820	1.59372639468018
H	-3.39920040684378	2.19912358156135	2.31356650098622
C	-3.93837833750149	1.73723102820113	0.28246311048465
H	-3.78393611607679	0.95892685217562	-0.46354442541351
H	-3.60055829450130	2.68564154279327	-0.13784224874764
H	-5.01341511438258	1.81936919597732	0.46172595471098
C	-3.75645517173403	0.09171561850327	2.19405192483843
H	-3.22249698359707	-0.18415700207505	3.10431681207173
H	-3.67615530404588	-0.72587149604988	1.47962546910420
H	-4.81243575623488	0.21444845887947	2.44740176478140
C	1.51184618650951	-1.76577382952887	1.97431087073088
C	2.06381296258334	-2.33289748886416	0.80318397071183
C	2.84814615272569	-3.48303622526698	0.86021787538692
H	3.25493606145693	-3.89187991508982	-0.05684841584779
C	3.09507936938868	-4.15028458054775	2.05313142175859
C	2.48132096349409	-3.63595263846292	3.19814528492480
H	2.59263967734720	-4.16053562164450	4.14126122424145
C	1.71355991413067	-2.48586077598576	3.16790376750415
H	1.23106266480999	-2.16153476868858	4.07688923540542
C	3.95906658670689	-5.38034513494658	2.10925448393837
H	4.15645837443998	-5.76839025863695	1.10896468594236
H	3.48633709790788	-6.17399132694737	2.69285197635529
H	4.92494072857647	-5.16849489063015	2.57804761464819
C	2.99626272558596	-0.55116936902340	-1.34932696245736
H	2.59431510755350	0.00956424685166	-2.19808195165088
C	4.19220724784813	-1.37840063512124	-1.82188511741803
H	3.94638937478946	-2.02738327905091	-2.66203162996027
H	4.58964737623414	-1.99659509346567	-1.01432794889555
H	4.99230602841776	-0.70804185180763	-2.14641544390998
C	3.41670951635559	0.44400197850765	-0.26625201528503
H	3.83100509769407	-0.07590565681263	0.59964492797355
H	2.57630261687390	1.04378482447924	0.07872386050901
H	4.18405529862122	1.11647512187038	-0.65667459578540
C	1.36004907633560	-2.91123627097728	-1.99497376384261
H	2.34771717606241	-3.36975871042596	-2.09417492239661
C	0.37024347922625	-3.98916249145043	-1.54957388120920
H	0.36140561456479	-4.79270532914740	-2.29023076869771
H	-0.63854470592086	-3.58627148256012	-1.47830455980401
H	0.64323656059745	-4.42269544923946	-0.58795081631485
C	0.95370765040300	-2.31681084090202	-3.34720499230664
H	-0.04897360743890	-1.89263328556816	-3.29846013309989
H	1.64383700062514	-1.54416597346039	-3.68977328866667

H	0.94096699286817	-3.10760162838794	-4.10110944926333
C	-0.40143879604171	0.57923826903183	-1.52281018028747
C	-0.23162701148312	1.37176523329886	-2.69260354457636
C	0.62095076583560	2.48865771820974	-2.68371121715404
H	1.15159554756808	2.74522631821591	-1.77769778250494
C	0.77935220367115	3.25208695722841	-3.82567644288342
H	1.43516772685965	4.11451557251594	-3.80509843693549
C	0.10852268199507	2.92711959867267	-5.00910123989708
C	-0.73183313898726	1.80987585112098	-5.01266840273090
H	-1.25702794908331	1.54245212372070	-5.92209449493963
C	-0.90753303183731	1.03854361328954	-3.87781755002952
H	-1.54832078049030	0.16688659540935	-3.89840980295700
C	0.31080500430985	3.74011273418627	-6.25532931918381
H	1.10806533505835	3.30837436723350	-6.86833206938331
H	0.59743100248805	4.76542308764017	-6.01930873087562
H	-0.59270128676644	3.76124721106667	-6.86619094666699
C	-1.84188202099636	-1.72542018030604	-0.16510733725366
C	-2.67806435151284	-1.81826613311298	-1.10944789628034
C	-3.10916496398900	-1.40636449190268	-2.37175034043744
C	-1.62821963298207	-2.54397161109565	1.08534899674967
O	-2.64863846719689	-1.76211839052535	-3.45949816477196
O	-4.17852331927277	-0.54277436643446	-2.31416474640757
C	-4.73360994364892	-0.14217966353864	-3.56816368804128
H	-5.58184226986659	0.49419976624751	-3.32393511988428
H	-5.06736228418294	-1.00745056371139	-4.14244729600461
H	-4.00992777964299	0.41764934487120	-4.16237008808339
H	-0.67559302274135	-3.07228172363833	1.07031389768253
H	-1.64715002033173	-1.93969427697981	1.99084435822365
H	-2.42618433009857	-3.28549865280003	1.14675129303263

Structure TS3

E = -2500.57264634 a.u. G = -2499.82533874 a.u.

98

Ru	0.15448483184856	0.06543464610521	-0.07925564668031
P	1.94152206812246	-1.38711612511792	-0.59041924335904
P	-1.01715426837337	1.60461315316416	1.24921023269312
N	0.75666387729559	-0.64237165666812	1.90859138263573
C	0.48552982976548	0.13641844150190	3.01207042266076
C	-0.45292320165213	1.19515777749453	2.90959952791833
C	-0.83156322814736	1.92883230680492	4.03645877228175
H	-1.59533529923572	2.69017125342728	3.93827815123646
C	-0.25494429352458	1.72859650977912	5.28140350224953
C	0.77118772051429	0.77783338458040	5.35082611751835
H	1.30172045962317	0.63703707072191	6.28700351775225
C	1.13434301285961	0.00961011324453	4.26365929178069
H	1.94367665564443	-0.69609810168896	4.37400987019238
C	-0.68515885844820	2.50992587956945	6.49289157179078
H	-1.18321445504655	1.86970217339083	7.22743281987857
H	-1.38269983575390	3.30352636138163	6.22082586403210
H	0.16859254524886	2.97052369583620	6.99725664706680
C	-0.41738270539388	3.33270194508982	0.88359096255852
H	-0.81026054946366	3.51623713110691	-0.11989403785427
C	-0.93124243241016	4.43795248272234	1.80597887645029
H	-0.62977278703956	5.40966659664446	1.40598517200312
H	-0.50780028788916	4.34918706230009	2.80671607581826
H	-2.01811264067668	4.44174757012134	1.89223525136096
C	1.11033694400134	3.34492106020350	0.81049589366877
H	1.48022549249853	2.65793566636264	0.04707859129611

H	1.55232256884492	3.05847862664370	1.76693790972540
H	1.46668050905185	4.34786537716535	0.56222852687412
C	-2.86021183510733	1.76117990799001	1.29554139044458
H	-3.07304709882152	2.55586603707517	2.01694298784618
C	-3.39804371157559	2.17498917793488	-0.07701413090454
H	-3.15846036469745	1.42331093435204	-0.82928259538125
H	-2.99922802214183	3.13529518652911	-0.40632905892629
H	-4.48594892973053	2.26617781410187	-0.03166467477956
C	-3.52072385088372	0.47458029088760	1.79255999975811
H	-3.09504542787484	0.13960460495504	2.73903969954173
H	-3.41415194282325	-0.32355133453548	1.06080361515184
H	-4.58820462245074	0.65115155789366	1.94552753672108
C	1.35674548771073	-1.88726370032513	2.01758017365234
C	2.02111993031644	-2.41828432701152	0.88790606368668
C	2.60839253608617	-3.68034029654059	0.92028429610957
H	3.10701444407462	-4.05660128338468	0.03496824560002
C	2.54864602172842	-4.49178145893164	2.04676423866344
C	1.84174847923546	-3.99237231279093	3.14446937348105
H	1.72296509292936	-4.61450101237117	4.02551609027216
C	1.26404935995729	-2.73585562801077	3.13935226787095
H	0.69303687805525	-2.42367241902244	4.00053873362209
C	3.19710523983955	-5.84884830308121	2.08098616706990
H	3.48815515126874	-6.17090284516184	1.08002169674026
H	2.52376174793533	-6.60298856156980	2.49568457852703
H	4.09843312381293	-5.84705115694166	2.70195876758099
C	3.57906405772834	-0.50064448135560	-0.68356542191732
H	3.47331829730711	0.18190828432228	-1.53211940939712
C	4.77255792279819	-1.42487042240094	-0.93039341572090
H	4.69726087516259	-1.96572789738665	-1.87362332217081
H	4.87733698745743	-2.15390453947364	-0.12441972838437
H	5.69131135917330	-0.83346956171377	-0.96212991820512
C	3.79230628797010	0.33203300161072	0.58382416265932
H	3.89280240749850	-0.31145271721808	1.45981517144509
H	2.96577943445343	1.01617876565430	0.76875259453316
H	4.70941776251430	0.91818726441338	0.48880003346048
C	1.92618636379995	-2.52159224181754	-2.05090693290154
H	2.82939440120463	-3.13316066363356	-1.97559307223571
C	0.70831700309218	-3.44698310144157	-2.04118930400049
H	0.78341561515345	-4.15087131552724	-2.87373580405120
H	-0.21498809837942	-2.88226856267126	-2.15749375183549
H	0.64240259645498	-4.02451947799177	-1.11932422162680
C	1.99401453852595	-1.70995023458364	-3.34691432553905
H	1.13133839465744	-1.05016169541610	-3.43826337605137
H	2.89665767830204	-1.10119328942194	-3.41240481051980
H	1.98666235321092	-2.38801439650925	-4.20337029622399
C	-0.35486376773092	0.65473879742780	-1.65324534012188
C	-0.61550625763814	1.30438020582368	-2.89582397705356
C	-0.31919904419259	2.67625494848498	-2.98791820697692
H	0.06517887674229	3.19385343831713	-2.12014526296685
C	-0.51062505252491	3.36038903579787	-4.17603037321464
H	-0.28600326675359	4.41951074973811	-4.22247237060677
C	-0.97508575526173	2.70519872471804	-5.31805805213141
C	-1.24162893556226	1.33474130016584	-5.22968896533684
H	-1.59541734555521	0.80388800412267	-6.10588128088228
C	-1.07889156810197	0.64199973121538	-4.04481827492274
H	-1.31120241522705	-0.41141135023285	-3.99721410482452
C	-1.20443605105270	3.45115975574475	-6.60124894043031
H	-2.26525741772855	3.69074858497611	-6.72319708734454
H	-0.90764398405877	2.85361294502419	-7.46490687249674
H	-0.64973297764177	4.38973462675623	-6.62042104717032

C	-1.39477295931471	-1.28440286480097	-0.34186761423463
C	-2.02384858639845	-1.07273173205688	-1.43895609009326
C	-3.02079179376365	-1.39985326908738	-2.37106347332460
C	-1.58345012375688	-2.33992401905010	0.71173228770881
O	-2.88279159651105	-2.09914641326067	-3.37613590925772
O	-4.20543680555061	-0.74387212911763	-2.12892923679477
C	-5.22917972339615	-0.92366467340887	-3.11146311661780
H	-6.07178735167411	-0.32544591350169	-2.77092833407943
H	-5.52069484586577	-1.97212081038983	-3.18756085321173
H	-4.89510759280621	-0.57900671042491	-4.09104682058090
H	-0.74275461373573	-3.03432614388850	0.72148679194146
H	-1.64844423393395	-1.90828173781989	1.70978605953201
H	-2.49476440819790	-2.90549307263844	0.50658012827801

Structure 16

E = -2500.64968640 a.u. G = -2499.89993240 a.u.

98

Ru	8.10268904125783	4.18310226036819	11.48445167933451
P	8.72741075600288	2.51754168622324	13.00450491533664
P	8.20751816886382	5.57748868394669	9.60709867848520
O	3.85246396753574	5.72114807268516	13.26742388325689
O	3.45578530219071	4.04100589568364	11.81885137577706
N	9.17155437666707	2.89550077068225	10.11057710470333
C	6.88551429546307	5.20120763105721	12.69524929937550
C	5.68493009682335	4.58912843982352	12.23035202895411
C	6.26202776662059	3.71539902334940	11.27574399191851
C	5.63025631235987	2.74996434130371	10.34621874497816
H	4.98595807114878	3.28435014258508	9.64300647072916
H	4.97416871563515	2.06423977057674	10.88743485687938
H	6.37255435311468	2.18551293762980	9.78572268835811
C	4.28921212648521	4.86289246685144	12.51928161196182
C	2.05375892966272	4.25841845192201	12.01028447982645
H	1.55433837386402	3.52855082185576	11.37754690760496
H	1.77424019784885	5.26966368818120	11.71357754545128
H	1.77626676710671	4.10594023862663	13.05368689374712
C	7.10148516739344	6.13452183804459	13.78029914358620
C	6.33515382917592	6.13831983472021	14.95601877263047
H	5.48897497137292	5.47370987539706	15.03907077000851
C	6.65986614590116	6.97341608452864	16.01252336223448
H	6.06469664492860	6.94033014696246	16.91816949956007
C	7.72946360976142	7.86831327910989	15.92969901844745
C	8.48590249925583	7.87865132617061	14.75513521521121
H	9.32407040133121	8.56059567977733	14.66658601613825
C	8.19116305882585	7.01731156288424	13.71280143631017
H	8.79766101072588	7.02149735407749	12.81649797094734
C	8.03954876877288	8.81078961831244	17.05856382283222
H	7.47190292453648	9.74071499991047	16.95329743983542
H	9.09787661357734	9.07553691523745	17.07628163221498
H	7.77443324381208	8.37624765582373	18.02355737028459
C	9.93320551798461	1.86467253964575	10.61135945280513
C	9.77865075659049	1.47047799909717	11.96945207153987
C	10.48167825221000	0.37567978329005	12.47779184285259
H	10.30750246484126	0.06911713639292	13.50221872903851
C	11.40879565790999	-0.33143276557136	11.72645242985822
C	11.64643079110845	0.13189507063901	10.42613101398353
H	12.41006298862106	-0.34860400076184	9.82282466855597
C	10.94254170917322	1.18779114434337	9.88441793987946
H	11.18336498491535	1.51588037891564	8.88423764738524

C	12.14293214026285	-1.52141562662047	12.28213881339302
H	13.22315007540066	-1.42962122049250	12.13981202946401
H	11.83387466921584	-2.44957906932058	11.79132297725823
H	11.95410985196099	-1.63557101592635	13.35074431325837
C	9.84971357931605	3.34014425976153	14.24154443575760
H	9.15826540562066	3.94584234742272	14.83461639148946
C	10.63408277250890	2.42540543657361	15.17892455251067
H	9.98753121549166	1.73755752649077	15.72555595772305
H	11.16987565015773	3.03011265847819	15.91582207116295
H	11.37301174589857	1.83781996568736	14.63280424041268
C	10.77491704380346	4.29484632108923	13.48439117992851
H	11.43443718911607	3.75262469144868	12.80408650593661
H	11.39776039904935	4.85941182636022	14.18254229235579
H	10.19847407886272	5.02333880043425	12.90619359612092
C	7.65889200031085	1.38621190592628	14.00619511882916
H	8.33434306715169	0.71421915985636	14.54386351801034
C	6.76777317883413	0.54267008351237	13.09388053564105
H	6.01696979957059	1.16196759315102	12.60504187617817
H	6.24402485089753	-0.21285500130037	13.68461709986553
H	7.34332712721060	0.03113097480039	12.32169095360795
C	6.82915333440106	2.17864159357543	15.02013732227947
H	7.45112650626898	2.73295436192010	15.72338693415014
H	6.20182730919700	1.49593719289453	15.59871112592563
H	6.17515122678199	2.88913418440312	14.51293700561874
C	8.92535234043077	3.07052270088415	8.76315191761553
C	8.46275724839823	4.33658041121909	8.31247562190764
C	8.17159716917644	4.55737717883162	6.96819618830519
H	7.82663246643413	5.53627536364191	6.65621667802351
C	8.27903497629818	3.55692792800741	6.00964722733654
C	8.67332144825888	2.29440645350876	6.46223008286720
H	8.72118549646956	1.46974963078651	5.75826087451342
C	8.98837102295299	2.05214902929474	7.78660094757013
H	9.24949331641368	1.04668664829065	8.08033265057096
C	7.96888985757608	3.81052391251159	4.55913771224439
H	7.52033745830417	4.79540985675654	4.41990780090188
H	7.27414089085570	3.06602272216555	4.16120868486648
H	8.87128991108298	3.76812814336431	3.94140617309929
C	9.74465206766219	6.64279442314268	9.58190373940935
H	9.61675696359019	7.33356005253975	10.42067482935199
C	10.98346943197269	5.78361101837576	9.84948607086965
H	10.90738412392338	5.22266344009752	10.77870855520915
H	11.86820714984857	6.42206728310322	9.91146143525641
H	11.14164370802061	5.06543468225069	9.04301551723639
C	9.92389931768650	7.45114841278193	8.29650401832297
H	9.99963725600341	6.79399250758046	7.42767478768527
H	10.85004632619085	8.02914332034795	8.35471231248372
H	9.10890207140974	8.15384651995332	8.12406677036019
C	6.86647808829111	6.71888918503552	9.02815636374808
H	7.20013719696696	7.14746315297843	8.07989957718858
C	5.54565488518018	5.98665212179530	8.78419250935104
H	5.66868997185948	5.12383289607829	8.13057551931597
H	4.83439372916853	6.66869470181527	8.31145253719505
H	5.10677056094359	5.64922511288533	9.72168189494981
C	6.68140367080078	7.85431133626433	10.04045891960923
H	6.38775544328624	7.46379699248892	11.01566442357113
H	5.89234200811607	8.52824214546981	9.69807143482215
H	7.58756857775929	8.44685298532701	10.17250495454357

Structure S3

E = -2785.757019996800 a.u.
108

Ru	0.29633057624623	0.09070830826865	0.17291398881708
P	2.11505506285404	-1.28431074182541	-0.31143609792287
P	-0.78770517073250	1.66168136356508	1.53472507475186
N	0.68798640250421	-0.67957318690675	2.15993269238842
C	-0.22517530397545	-0.42016277685271	3.18070580516935
C	-1.04312333515544	0.72453935358084	3.07112075851005
C	-2.01302011903846	1.00331491673032	4.02793796802017
H	-2.62560561845961	1.89062190121245	3.92215039261465
C	-2.24192437626699	0.16017045452441	5.11234983941025
C	-1.46395236700001	-0.99629420855589	5.19214474616854
H	-1.63957915533109	-1.69903502194543	5.99986415527261
C	-0.47962606963920	-1.28329166480585	4.26014977002131
H	0.07847625030318	-2.20411097029606	4.34995840682820
C	-3.28018766632780	0.47963125570474	6.15378017213762
H	-3.99081544065771	1.22274270939778	5.78872989471619
H	-2.82221726061121	0.88228700914835	7.06272882966349
H	-3.84127833659058	-0.41123146946271	6.44483687402150
C	0.31502442597532	3.08856624755835	2.01895191026671
H	0.49023019303885	3.61665893986944	1.07689299537152
C	-0.31375567629822	4.05204845595507	3.02635479793011
H	0.39608507007337	4.84903472274375	3.26295306588466
H	-0.55753417790927	3.54086688490256	3.96000684217473
H	-1.22164723048997	4.52281755013543	2.64850246992908
C	1.65875259949329	2.56291905600774	2.52956854354760
H	2.12954019271886	1.90318135392347	1.80275803033908
H	1.53204514963342	2.00366323607260	3.45874488346968
H	2.32934647375870	3.40195873774808	2.73127338071931
C	-2.41966732675282	2.45586560531120	1.15485864790458
H	-2.71300627381489	3.02637316999537	2.03984216974622
C	-2.27725576336335	3.42352716146725	-0.02094796523324
H	-1.92555374813965	2.90343290677135	-0.91197651586482
H	-1.58138097257053	4.23625353006412	0.19012981171918
H	-3.24790102565425	3.86853967814233	-0.25305416999774
C	-3.49265567734491	1.40427008869527	0.87690515404656
H	-4.44540075777439	1.89602443715435	0.66442568766158
H	-3.64018809987284	0.74223534048725	1.72946271926444
H	-3.22789357241419	0.79258069402532	0.01490429896499
C	1.91105951456252	-1.27435072405139	2.43084769614393
C	2.72469737336169	-1.68855533030443	1.35066346284886
C	3.96705521899907	-2.27584328960062	1.57895790622766
H	4.57577627248255	-2.57378458941624	0.73447209431872
C	4.48279339262432	-2.45846375972669	2.85704074807206
C	3.70785491108211	-1.98633348830617	3.91825799275075
H	4.09262667069923	-2.05727466858786	4.93044791815792
C	2.46628024582590	-1.40940539786509	3.72036244976104
H	1.93180992410510	-1.02930768410635	4.57735176105164
C	5.81686822292879	-3.11541156864884	3.08689390239254
H	6.40080343398076	-3.15511401513682	2.16586045285745
H	5.70117607213540	-4.14298194635516	3.44620076016596
H	6.40376172290522	-2.57818224323904	3.83579578319845
C	3.58632576528762	-0.50010532191089	-1.12827132235876
H	4.39902111022143	-1.22480808076322	-1.02893139089069

C	3.98953633892103	0.78149611253007	-0.39930569295634
H	4.88613398500715	1.20509945896217	-0.86006920208746
H	4.20365994333874	0.59825887954831	0.65389091987027
H	3.18892695122041	1.52043947704004	-0.45747817605134
C	3.34877950932825	-0.23437293449674	-2.61527597190006
H	4.28408568312486	0.08049419743489	-3.08540400226323
H	2.62140419764968	0.56284399873520	-2.75733567355142
H	2.98924619313704	-1.11433866988523	-3.14975580371415
C	1.88199591502163	-2.91270751136955	-1.19353574145876
H	1.33835411022643	-2.63532630697261	-2.10152868821832
C	3.14773731165766	-3.67321307421729	-1.59013212609917
H	2.86822914300294	-4.57229835712538	-2.14616857030995
H	3.70700611137341	-3.99804681193080	-0.71170304757241
H	3.81452829709620	-3.08933521619852	-2.22421899968207
C	0.96914540126513	-3.78907196815592	-0.33606125104184
H	0.10408046875310	-3.22432600065008	0.00227009559119
H	1.49947687258744	-4.15023984827340	0.54681448357655
H	0.61741177085556	-4.65151656415950	-0.90450228822047
C	0.13753938120535	0.78375239446554	-1.38861956054021
C	0.08447777516912	1.36421602777541	-2.68901915689736
C	0.10399463184202	2.75467621294994	-2.86284809778038
H	0.18605471647358	3.39540472447094	-1.99510699292595
C	0.01501197845598	3.30252400605994	-4.13187762538020
H	0.01891244612452	4.37992328842059	-4.24983127449416
C	-0.07720754004969	2.49009118563316	-5.26327515498605
C	-0.06120007816558	1.10389287222001	-5.08681378597154
H	-0.12853219068208	0.45652433402033	-5.95260661759052
C	0.00902533159077	0.54523669653967	-3.82630135860171
H	-0.00149536686752	-0.52854057358470	-3.69994456234126
C	-0.22613541046507	3.08280370920526	-6.63475030482914
H	0.06879831887724	4.13243342666495	-6.65108344597938
H	-1.26819459426440	3.02277321765283	-6.96393031544506
H	0.37422034624489	2.54229790837074	-7.36902729681595
C	-2.75100462521092	-0.93156996795668	-2.15977021391503
C	-2.47718337963804	-1.56701267960752	-1.17229293142675
N	-2.34005656220409	-2.40874280567883	-0.14357280942110
C	-2.49000391570375	-2.03585479678132	1.26213688120860
C	-2.07785266825916	-3.33830788440125	1.93789286799371
O	-2.27710432215284	-4.35601115622037	0.92379842588822
C	-2.34227138962588	-3.78798586431841	-0.30080635945097
O	-2.38945346486681	-4.40590541946062	-1.32977118007978
C	-3.05109339642623	-0.18621183538867	-3.32823463902164
C	-3.14272258028748	-0.83457107943200	-4.56945225164587
C	-3.25554600347245	1.19940770750309	-3.27355812411427
C	-3.41860819927818	-0.11085110426250	-5.71924443901601
C	-3.52840154980328	1.91433859787976	-4.42843219444320
C	-3.61048139824180	1.26585926819701	-5.65660215908238
H	-2.98921562798165	-1.90468425497446	-4.61864317225410
H	-3.18390269579723	1.70791222281952	-2.32378703735992
H	-3.48276635953816	-0.62463797390162	-6.67049222747937
H	-3.67091590820772	2.98611738325704	-4.37018492769727
H	-3.82225413350772	1.82828515445779	-6.55703529457044
H	-1.83295378187152	-1.21038976286708	1.50715957636999
H	-3.52496634126678	-1.75898744549755	1.47284829582161
H	-2.69034531930888	-3.59392698949369	2.79747021838991
H	-1.02463605202112	-3.33670552604523	2.21600173884379

Structure S4

E = -2785.734586328982 a.u.
108

Ru	0.25202290806780	0.08821903017348	0.06277620977313
P	1.95331307751021	-1.48553313384097	-0.36390570632817
P	-0.85035033255866	1.65858601726657	1.45530653950190
N	0.61280987721376	-0.72334206954516	2.10047778421038
C	-0.24034343627135	-0.38891803723817	3.14136368475452
C	-1.04746144152501	0.75880728924292	3.01487807464157
C	-1.95730467697151	1.10771458921422	4.01169010767416
H	-2.56501503555150	1.99626411210216	3.89318643630968
C	-2.12814816884711	0.33682331931365	5.15496457919170
C	-1.35655486908582	-0.82540064222155	5.25913457187113
H	-1.49169981694964	-1.47636121631711	6.11651832983550
C	-0.43816011731785	-1.18157939385645	4.28947531160190
H	0.11108908541735	-2.10628548085435	4.39520806906808
C	-3.10280978735995	0.72629627162136	6.23294410408609
H	-3.75363723070987	1.53702343971345	5.90236145886662
H	-2.58503586007778	1.06522061444692	7.13545424213011
H	-3.73504625826065	-0.11620148899512	6.52415887467213
C	0.25214694525795	3.11021300137660	1.84493787209241
H	0.33729395053454	3.64263513893265	0.89271968854405
C	-0.30434999061355	4.06421052914256	2.90155691507172
H	0.39705150933053	4.88791395670344	3.05752322919094
H	-0.43556563623480	3.55663243859138	3.85901192795395
H	-1.26142948536339	4.49803567500194	2.61056972270251
C	1.64390223914361	2.60316287835092	2.23430063991544
H	2.06387669248593	1.94913648044260	1.46870735210339
H	1.61080957109899	2.04124903804785	3.16975701939339
H	2.31976763414364	3.45026460109121	2.37403992607328
C	-2.51568689016837	2.40370314220231	1.12446729378623
H	-2.75555223710878	3.00296238932082	2.00686514172735
C	-2.48082698437975	3.33434707523298	-0.08672831398930
H	-2.21610278731809	2.79067811896682	-0.99211022220322
H	-1.77201638396670	4.15381643182917	0.03884021389661
H	-3.46982750754970	3.77345676265895	-0.23852126204105
C	-3.59412987605083	1.33133466677764	0.96225943229006
H	-4.56437583749509	1.81314915460335	0.81676946308950
H	-3.66680066130389	0.69174382840525	1.84084449687214
H	-3.39327013417328	0.70257869089607	0.09647610286996
C	1.85494625121825	-1.26312755229895	2.36476591337787
C	2.63746561691025	-1.73899939277224	1.28786312136008
C	3.89157179906989	-2.30810687446123	1.50419935855795
H	4.46190703997701	-2.67214640191265	0.65777592005836
C	4.45873650677927	-2.39680784818253	2.76814800160926
C	3.72152163666939	-1.85033485942758	3.82386473922420
H	4.14827297682498	-1.84466519210434	4.82169891580646
C	2.46848753566765	-1.29888479641701	3.63816430039435
H	1.96548409992045	-0.86537047529539	4.48889032486882
C	5.80570718917920	-3.02816051472339	2.99362687237309
H	6.31806456320009	-3.21099882489421	2.04768972078184
H	5.71758342369234	-3.98865875464619	3.51100615453473
H	6.44929059299157	-2.39243495995546	3.60730927914073
C	3.39544288165078	-0.87246125830182	-1.35771840659230
H	4.15062951242433	-1.65995136398663	-1.29533896284444

C	3.97386118968308	0.40748182390741	-0.75170443100677
H	4.83795462186790	0.73315632644245	-1.33675926203607
H	4.29580818317939	0.26313350417108	0.27911490838583
H	3.23328350835183	1.20954027034824	-0.76956773780271
C	3.02989235813939	-0.65505058560012	-2.82585164972634
H	3.93885819807235	-0.51663469941486	-3.41667348883036
H	2.42472756684810	0.24132979798035	-2.93728775384949
H	2.47603003281427	-1.48918134243034	-3.25741288900950
C	1.55186451482947	-3.19390895306110	-1.01075509277307
H	0.76978816863534	-3.02906281340880	-1.75531126466971
C	2.71236903336330	-3.95002756730616	-1.66138247793252
H	2.35906270323076	-4.93260909525448	-1.98528398564807
H	3.52654834759331	-4.11423679512313	-0.95284035897776
H	3.11561530293340	-3.44202133852429	-2.53583078779261
C	0.96627065183880	-4.02948668111597	0.13433640977115
H	0.26265563124115	-3.46320772760225	0.74062820685032
H	1.76113925778844	-4.37784089614907	0.79488882219175
H	0.44748687509049	-4.89989988640618	-0.27084069096090
C	0.16742533289397	0.94049965745889	-1.45487044507879
C	0.09473542416283	1.68333328446225	-2.65757959105542
C	-0.05689275122938	3.08358410317101	-2.61750043058386
H	-0.09799758710781	3.58367385373869	-1.66102549925453
C	-0.15113991195712	3.81333503060739	-3.78545223175644
H	-0.27696286476979	4.88866003449990	-3.72996474878027
C	-0.08518791120434	3.19156518029657	-5.03825633168222
C	0.09477400119454	1.80930395360984	-5.07575567541477
H	0.14868896454525	1.30513264295172	-6.03271515293479
C	0.18032040222896	1.06177890630267	-3.91586952901182
H	0.30731691920790	-0.00714831298889	-3.97230189194696
C	-0.20163707721400	3.99837981007968	-6.29980723899851
H	0.58917030729244	4.75102905398549	-6.36060583223765
H	-1.15517365021442	4.53176167975175	-6.33635771710976
H	-0.13486466347765	3.36346748589863	-7.18315702196674
C	-1.92938032403676	-0.61598278425318	-1.79430090093011
C	-1.44769067534868	-1.00605363318894	-0.68385235121608
N	-1.83873266724307	-2.15477285463378	0.04104781081907
C	-2.46611162781310	-2.13425999965875	1.35586108764538
C	-2.61418177120190	-3.62693357406994	1.62149789571866
O	-2.60671822275964	-4.22769803133825	0.30840380393789
C	-2.08069624663405	-3.34712184342101	-0.58975879442410
O	-1.86758206794834	-3.64921922496683	-1.73845086888341
C	-2.44615868480391	-0.27232856932824	-3.02751565767951
C	-2.13113371092235	-1.03542128265039	-4.18456877912414
C	-3.25980774613989	0.87806504300250	-3.20879437621084
C	-2.60218796032378	-0.66473436286384	-5.42987835340122
C	-3.70541404012428	1.24164712512971	-4.46412588872608
C	-3.38274654476892	0.47983806902487	-5.58917511915134
H	-1.53019436047141	-1.92677000336167	-4.06535500405302
H	-3.51489506816870	1.47996308860612	-2.34779548660688
H	-2.35216194061905	-1.27130475983255	-6.29249397211336
H	-4.31377241832434	2.13189052810879	-4.57301138651103
H	-3.73915360913739	0.77032975600548	-6.56885702660331
H	-1.84506039758123	-1.64430492313736	2.09371884433015
H	-3.43347473830341	-1.62618921123403	1.30722923820546
H	-3.54624491319421	-3.89252654770021	2.11406499170282
H	-1.76998501715066	-4.02739485893757	2.18563159105348

Structure 14

E = -2785.820586931000 a.u.
108

Ru	0.28065542212457	0.10352041029940	0.07022952945104
P	1.87413391553393	-1.56189478229368	-0.38356359893684
P	-0.96207658524325	1.70709499638790	1.20490398588066
N	0.79624416718001	-0.42004090323649	2.10951063528851
C	-0.06580784814972	-0.04267023810132	3.12098589306406
C	-0.99462164138809	1.00456563808980	2.87759820327956
C	-1.90662704870920	1.39506055017617	3.85599933608659
H	-2.59441172780448	2.20447805138855	3.64144940402572
C	-1.98883390504351	0.76628302648504	5.09250479311073
C	-1.11989547834089	-0.30761112672347	5.30806035941468
H	-1.18330188306192	-0.86241498855487	6.23871501934686
C	-0.18832745718426	-0.70082400293913	4.36499790786087
H	0.42991004538161	-1.56063497267359	4.57501792600197
C	-2.96921324444538	1.20828675807714	6.14501940922373
H	-3.69966668561471	1.90697244200603	5.73390229152701
H	-2.46613000233634	1.70994551939652	6.97764894189187
H	-3.51523629660924	0.36030224964472	6.56658700755043
C	0.03824505144553	3.27612907112364	1.34327317121639
H	0.01979664935536	3.69817031008016	0.33441721795947
C	-0.51835256234032	4.30476784260786	2.32545378380858
H	0.12750484924579	5.18697283622617	2.34423321492211
H	-0.55716272972254	3.89945944048137	3.33840391990545
H	-1.52076968336485	4.63776299146893	2.05348639857904
C	1.48738471087577	2.91292614358449	1.68186336929123
H	1.92254351282972	2.23283714530099	0.94584014665764
H	1.55607338481627	2.42813279180236	2.65696336238259
H	2.10451712191327	3.81450103303011	1.70543111986064
C	-2.68615039380718	2.28488320845233	0.83818474505100
H	-2.96458478230277	2.99444381343324	1.62186031585337
C	-2.72893106202327	3.00596326342361	-0.51220827980459
H	-2.49074012289428	2.32156132540805	-1.32542666803787
H	-2.03270738753088	3.84336135410059	-0.56410290256494
H	-3.73388718141302	3.39821547105612	-0.68662960254476
C	-3.68273779780606	1.12384788333449	0.86069409004781
H	-4.68892218978541	1.49882347662525	0.65707784435898
H	-3.70333242781246	0.62236836175241	1.82800161217734
H	-3.44007708693793	0.38912233533270	0.09357218382101
C	1.96342624566737	-1.12256533585157	2.34017498295661
C	2.58693349718884	-1.81038521935341	1.26624044904234
C	3.75913836856324	-2.53822257366027	1.46897840450721
H	4.20721125179003	-3.05720631620358	0.62923761705591
C	4.40093245727450	-2.59632759170777	2.69863252818469
C	3.83431797149654	-1.84677578408673	3.73532159817263
H	4.33358685957606	-1.81036720362538	4.69832405236440
C	2.66377612263832	-1.13246886655233	3.56926481814124
H	2.29659822740472	-0.54216380232817	4.39536592450794
C	5.65717397356212	-3.39848907306246	2.90431398984928
H	6.05322703403100	-3.75903294771638	1.95364597617606
H	5.47729458350150	-4.27187764747828	3.53898535739153
H	6.43691534137412	-2.80597152311336	3.39029893300286
C	3.32669698123801	-1.03979616383222	-1.42028175690575
H	4.02983931781675	-1.87669694120482	-1.42205976830601
C	4.02763476639503	0.17188075743755	-0.80314208419924
H	4.86487820430373	0.48060175599318	-1.43460717590478
H	4.41353454273859	-0.04561210104477	0.19226035120298

H	3.34769208860131	1.02212006305302	-0.72186150335713
C	2.89085824764368	-0.75194324146644	-2.85929181941686
H	3.77011042300323	-0.59175639710796	-3.48893022395489
H	2.27688432850453	0.14571957288343	-2.90579899234800
H	2.31384158792250	-1.56765792733234	-3.29570757571915
C	1.38306180758289	-3.24536495868612	-1.03989258188061
H	0.58831244162894	-3.01732097552694	-1.75577133753816
C	2.47172396840301	-4.04539469508661	-1.75988749305444
H	2.04830674647234	-4.99361867632070	-2.10200195275411
H	3.30227711407347	-4.28233421668299	-1.09230300593340
H	2.87092901967770	-3.53271420398500	-2.63291861597976
C	0.78975730873491	-4.09380428175287	0.08878662391326
H	0.12104853164073	-3.52912408288545	0.73223709073306
H	1.58217633697408	-4.49204356354814	0.72417965222606
H	0.23217174736411	-4.93346736223767	-0.33082153209415
C	-0.24485702245984	0.60683970810839	-1.77495926966018
C	0.00746734921278	1.75101565578393	-2.62980777876209
C	1.11220099387157	2.57441173012547	-2.35035549205473
H	1.77138189712977	2.29203765459148	-1.54123485946979
C	1.36729247342846	3.71936396840787	-3.07946256270185
H	2.23449274352575	4.32552917741202	-2.84245077879056
C	0.51632800400734	4.11193336584635	-4.11856721026607
C	-0.59068385442670	3.30911577870923	-4.39519343471220
H	-1.26886205950215	3.59625816329144	-5.19054222244080
C	-0.84021413731040	2.15093349910659	-3.67457748869508
H	-1.71245702831401	1.56228184073238	-3.91254506827781
C	0.78232947371378	5.37062063418415	-4.89455279013852
H	1.82136273490483	5.41631570240784	-5.22836821746517
H	0.60578725347928	6.25441083824458	-4.27434954447267
H	0.13640842707932	5.44224897461716	-5.76975417422694
C	-1.30678763326985	-0.46628986941552	-1.96952722993319
C	-1.19303017141635	-0.96720431552066	-0.68269349637233
N	-1.89531290059631	-1.99329577851399	-0.09165313714037
C	-2.02998784850742	-2.11543057556491	1.35890350725862
C	-3.04502972101371	-3.24803568578710	1.45968755860550
O	-2.92020275559651	-3.95426702289143	0.20568187467853
C	-2.30952716144828	-3.16409287060843	-0.71296982701051
O	-2.14108338263305	-3.50119777520490	-1.85328583359105
C	-2.19767598240006	-0.78834046568360	-3.08730031737883
C	-1.68475340261579	-0.98114090271490	-4.37475431655381
C	-3.57919669538950	-0.90618441920172	-2.90315740038574
C	-2.52212647027312	-1.27880596435788	-5.44051711875256
C	-4.41877889865265	-1.20808300492829	-3.96705099544750
C	-3.89518955856196	-1.39465819934651	-5.24239593243150
H	-0.61624457003579	-0.90072183936874	-4.53159109575611
H	-3.99785997835679	-0.75643813569642	-1.91589633838000
H	-2.10266213072644	-1.42948519004177	-6.42797613451988
H	-5.48583388390222	-1.29560634053903	-3.80122683540678
H	-4.54909714921895	-1.63107514968228	-6.07243126103638
H	-1.07373441305485	-2.35969387618395	1.82013622086186
H	-2.38600279915394	-1.18970089139473	1.79726735855362
H	-4.06888162993637	-2.88043779096065	1.54390934302866
H	-2.83574917539761	-3.94762282994150	2.26462828215440

NMR and IR Spectra

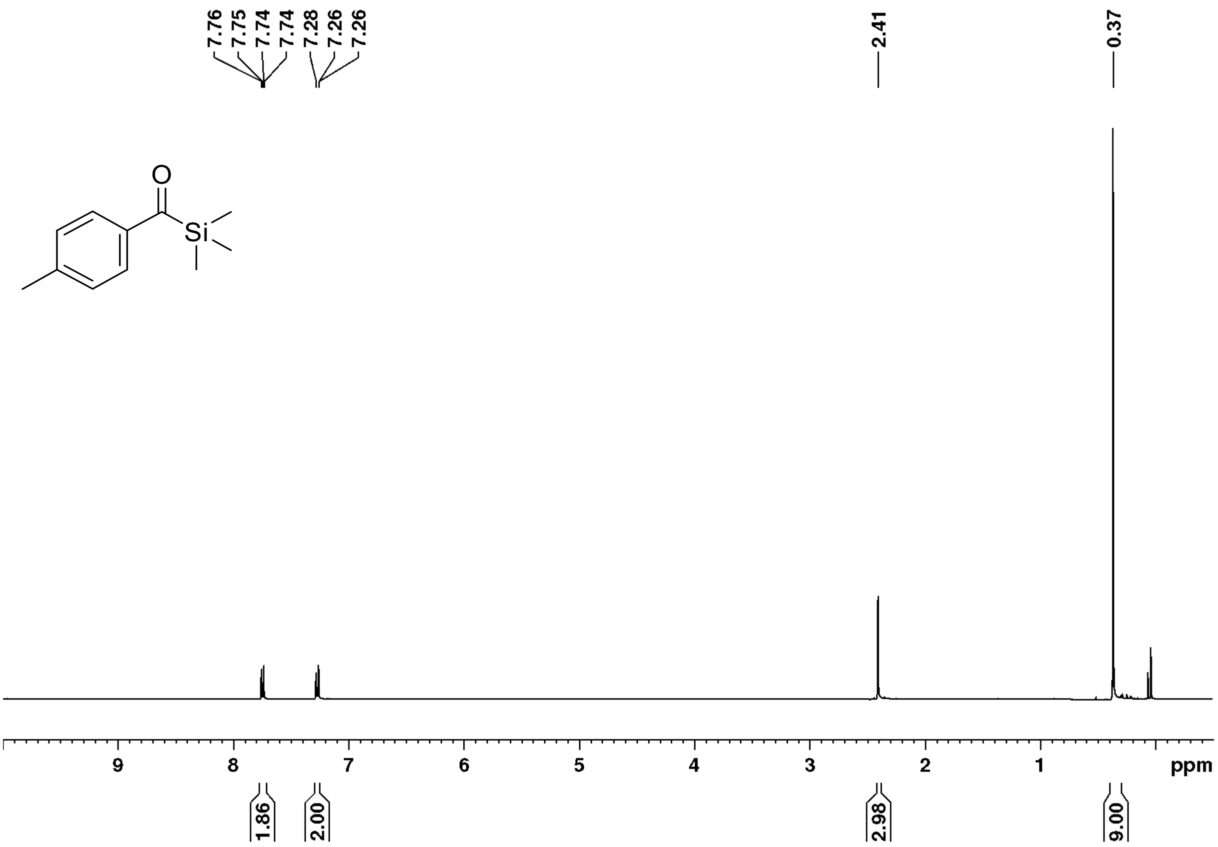

^{1}H NMR spectrum of *p*-tolyl(trimethylsilyl)methanone (**S1**) in $CDCl_3$ at 400 MHz

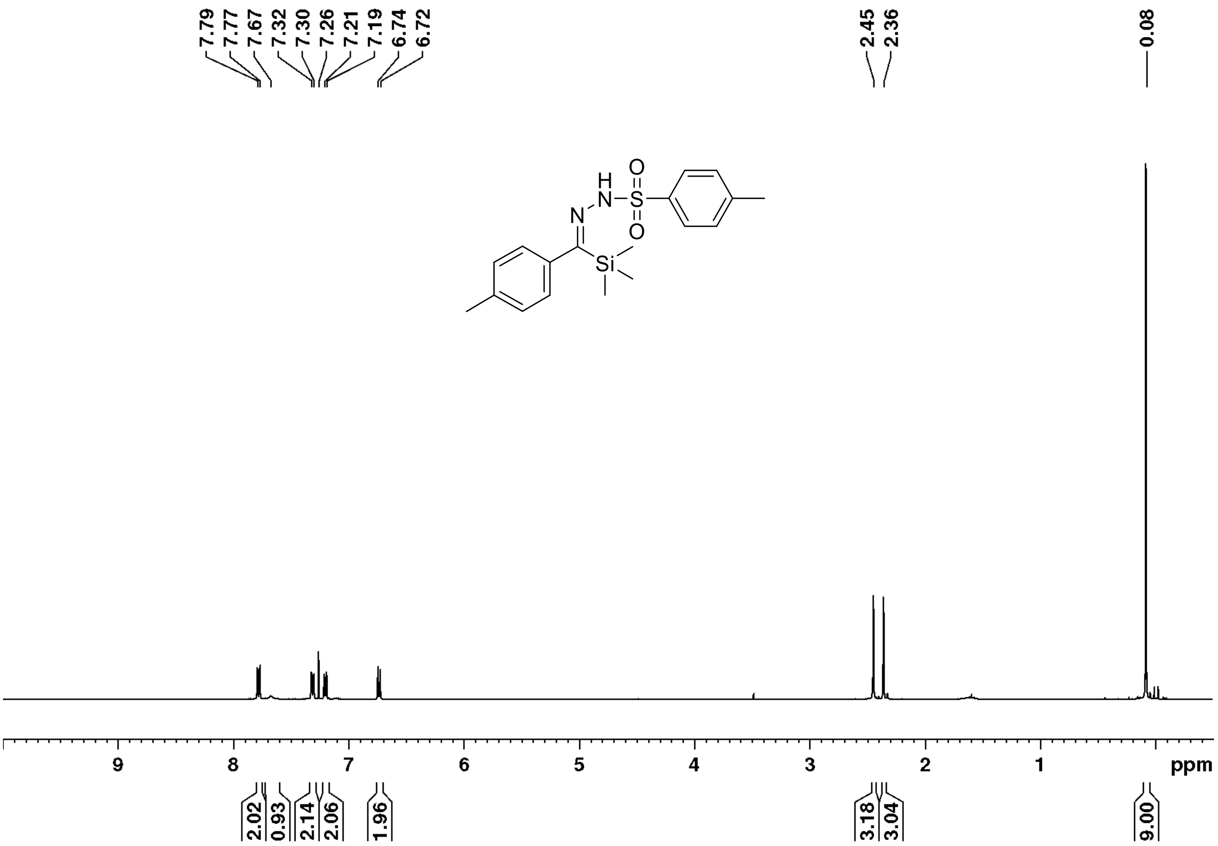

^{1}H NMR spectrum of **S2** in $CDCl_3$ at 400 MHz

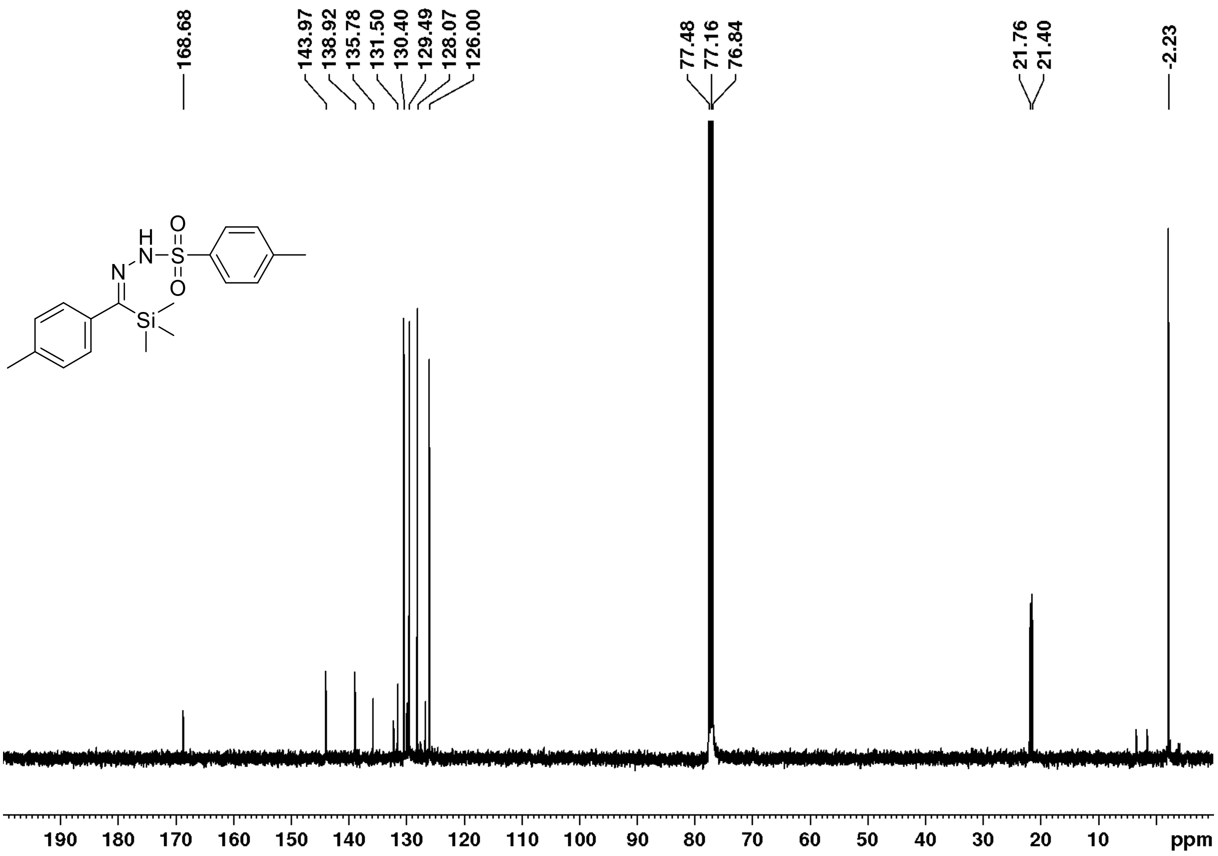

^{13}C{^{1}H} NMR spectrum of **S2** in $CDCl_3$ at 101 MHz

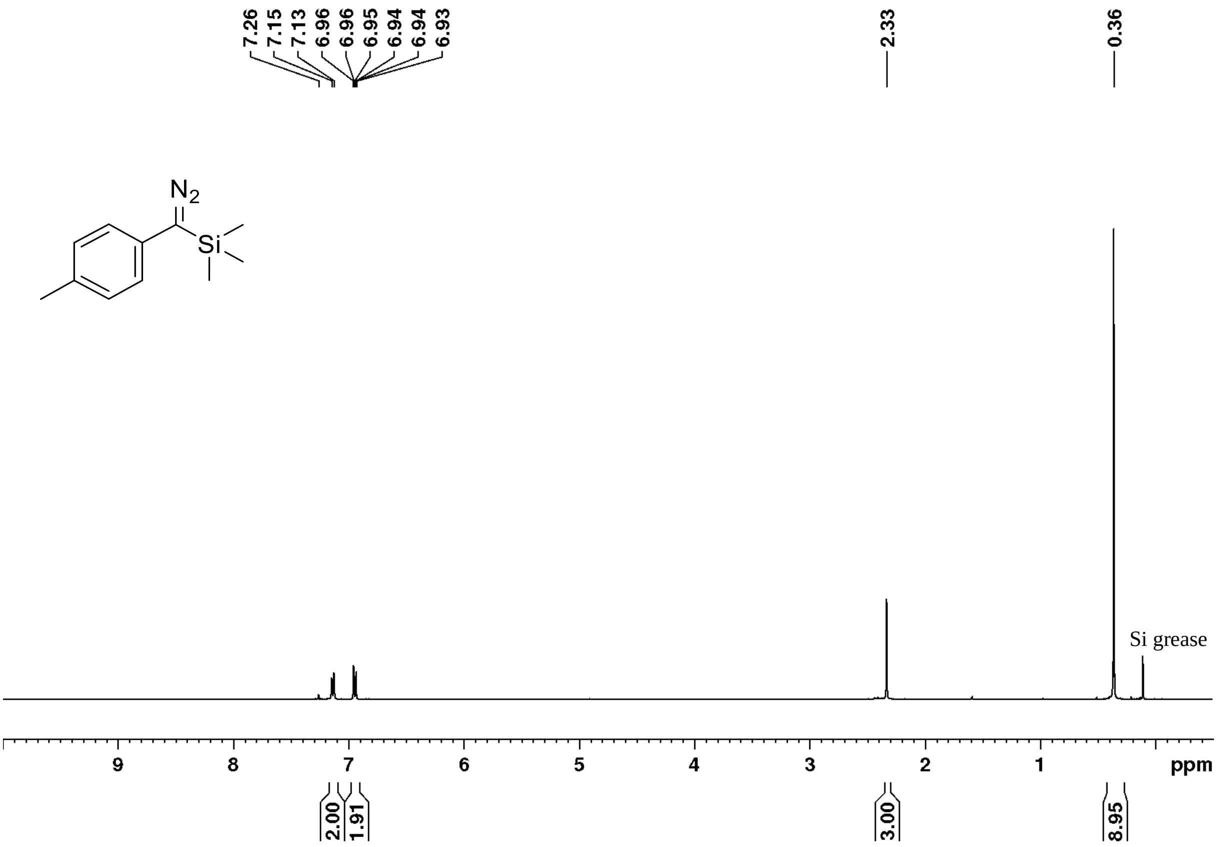

^{1}H NMR spectrum of **8** in $CDCl_3$ at 400 MHz

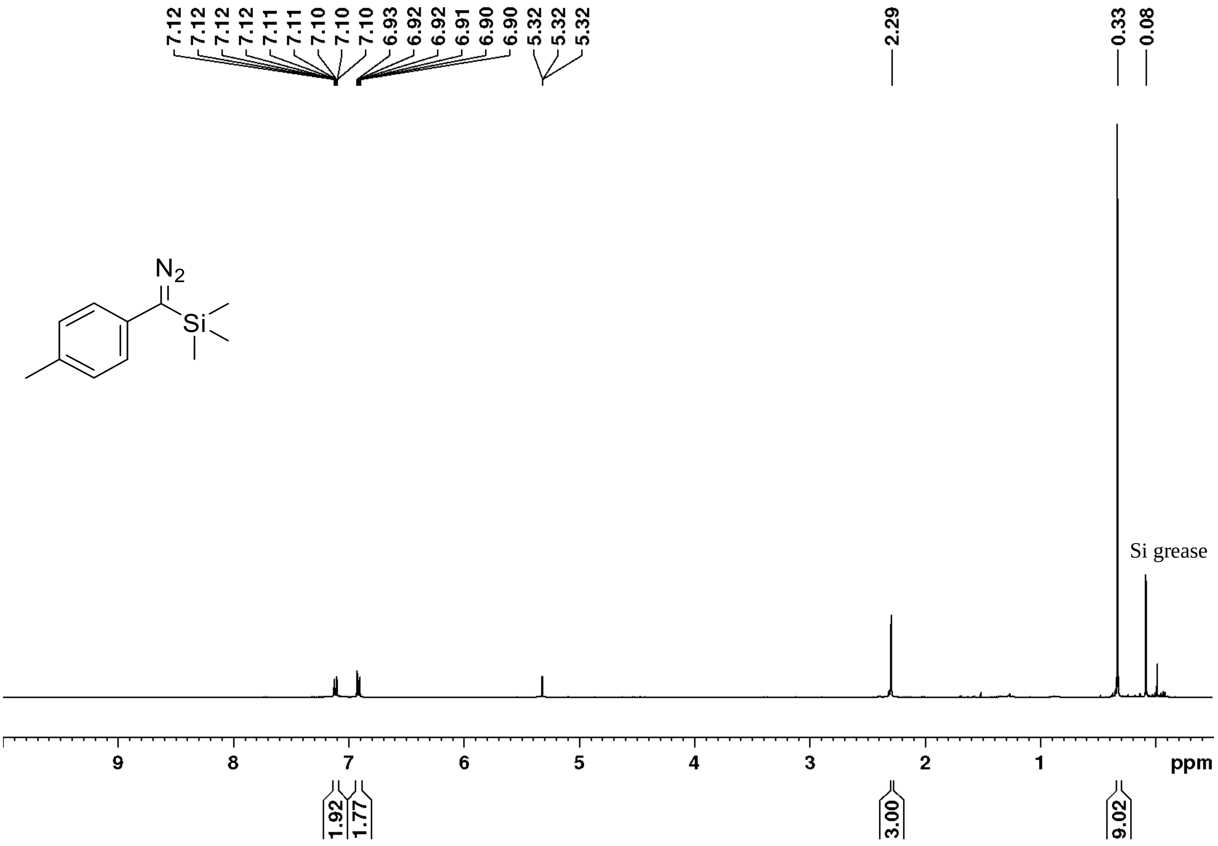

^{1}H NMR spectrum of **8** in CD_2Cl_2 at 400 MHz

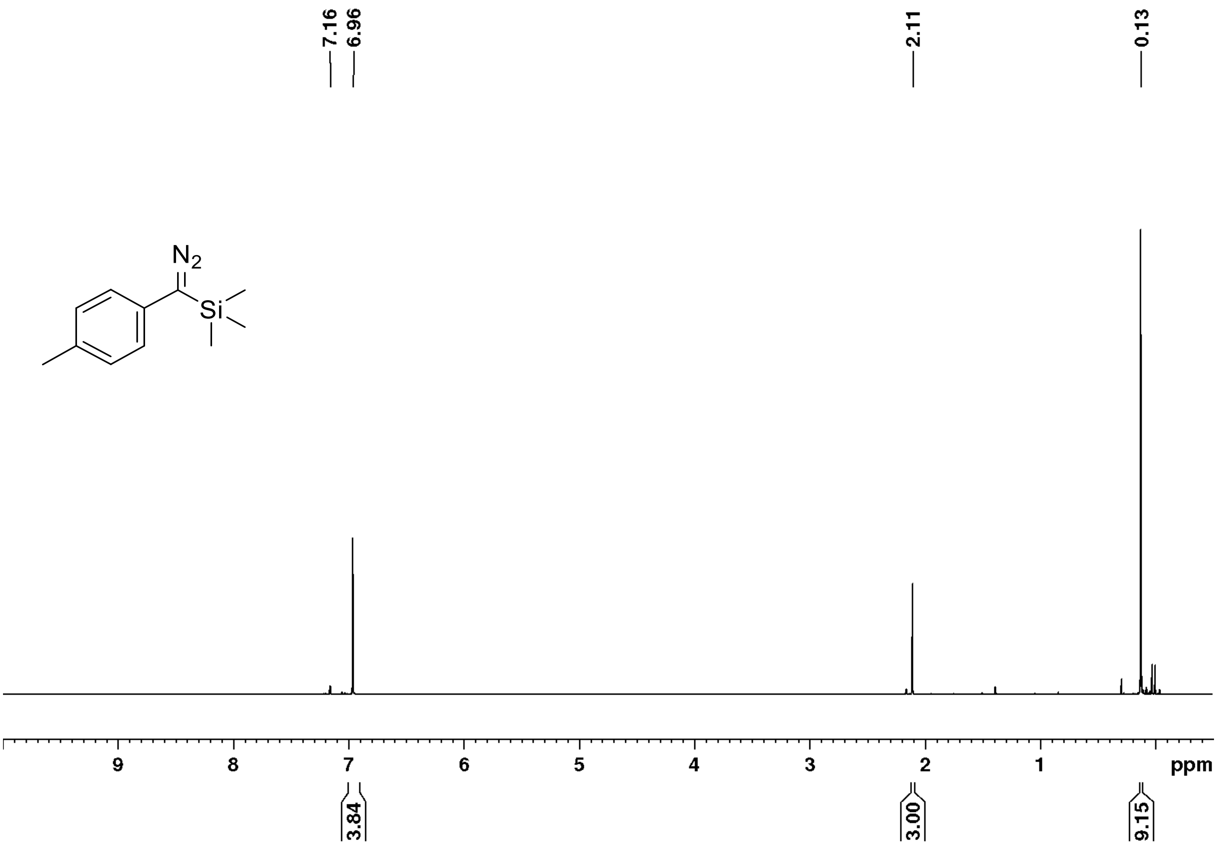

^{1}H NMR spectrum of **8** in C_6D_6 at 400 MHz

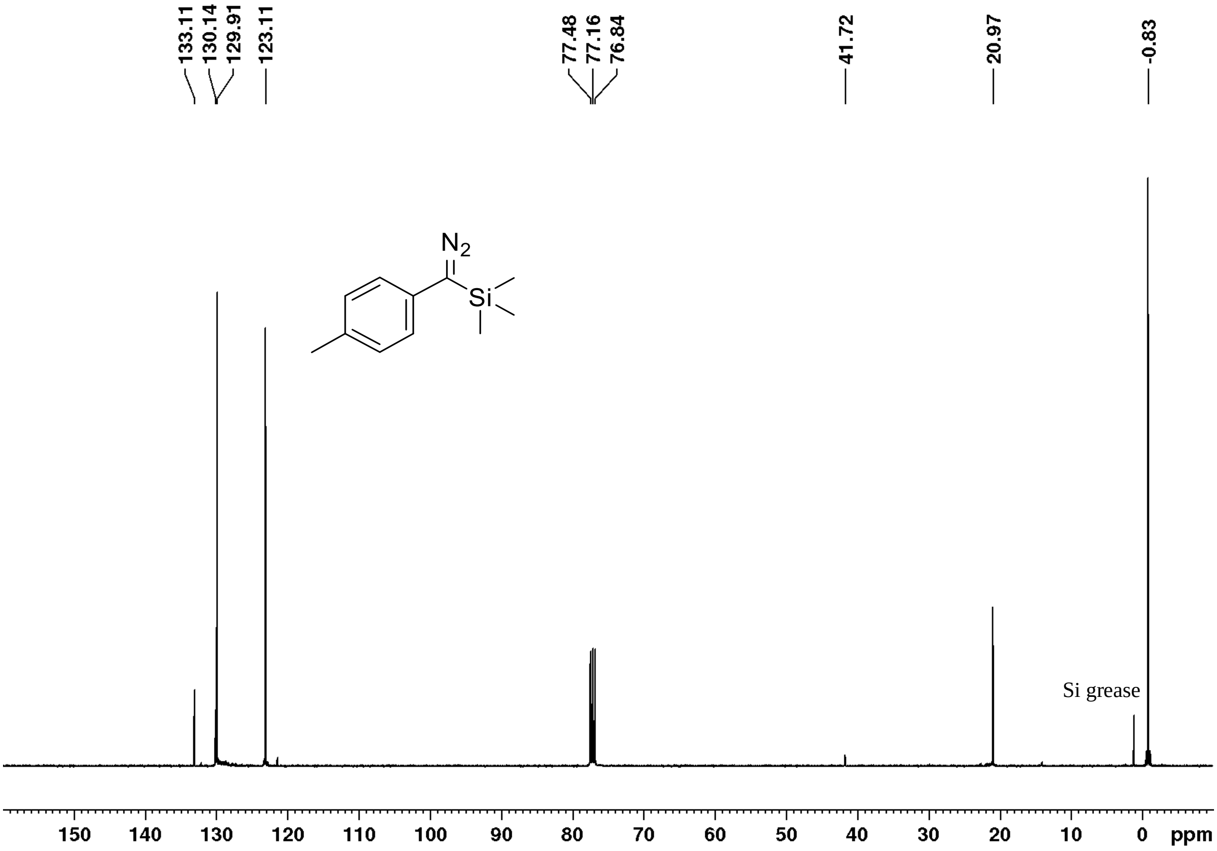

^{13}C{^{1}H} NMR spectrum of **8** in $CDCl_3$ at 101 MHz

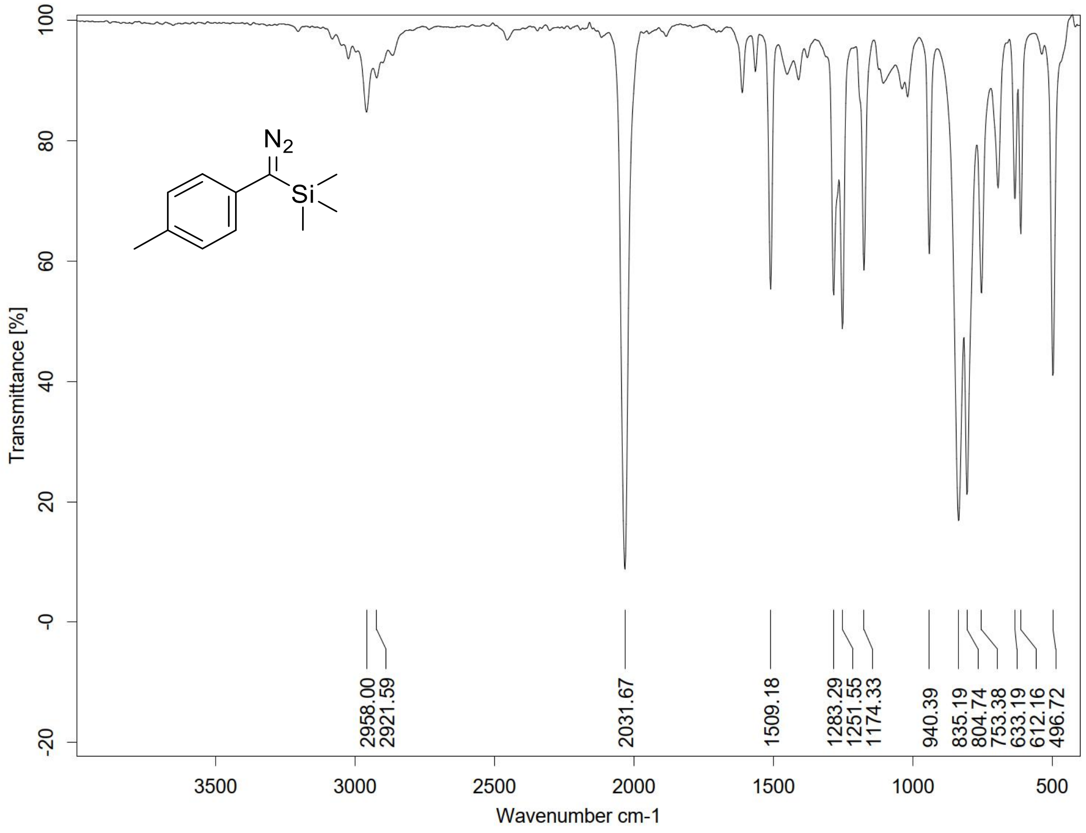

FT-IR spectrum of **8**

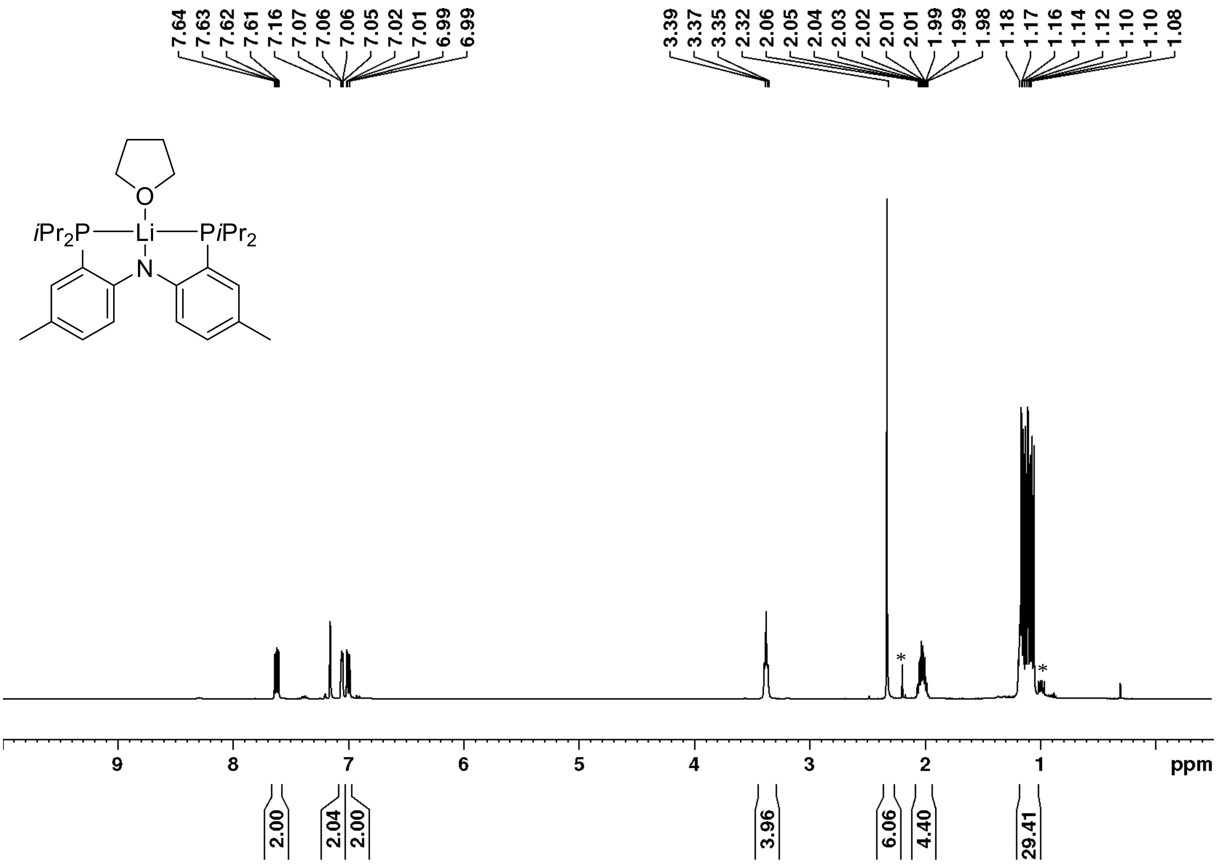

^{1}H NMR spectrum of **10** in C_6D_6 at 400 MHz. *^{iPr}**PNPH**

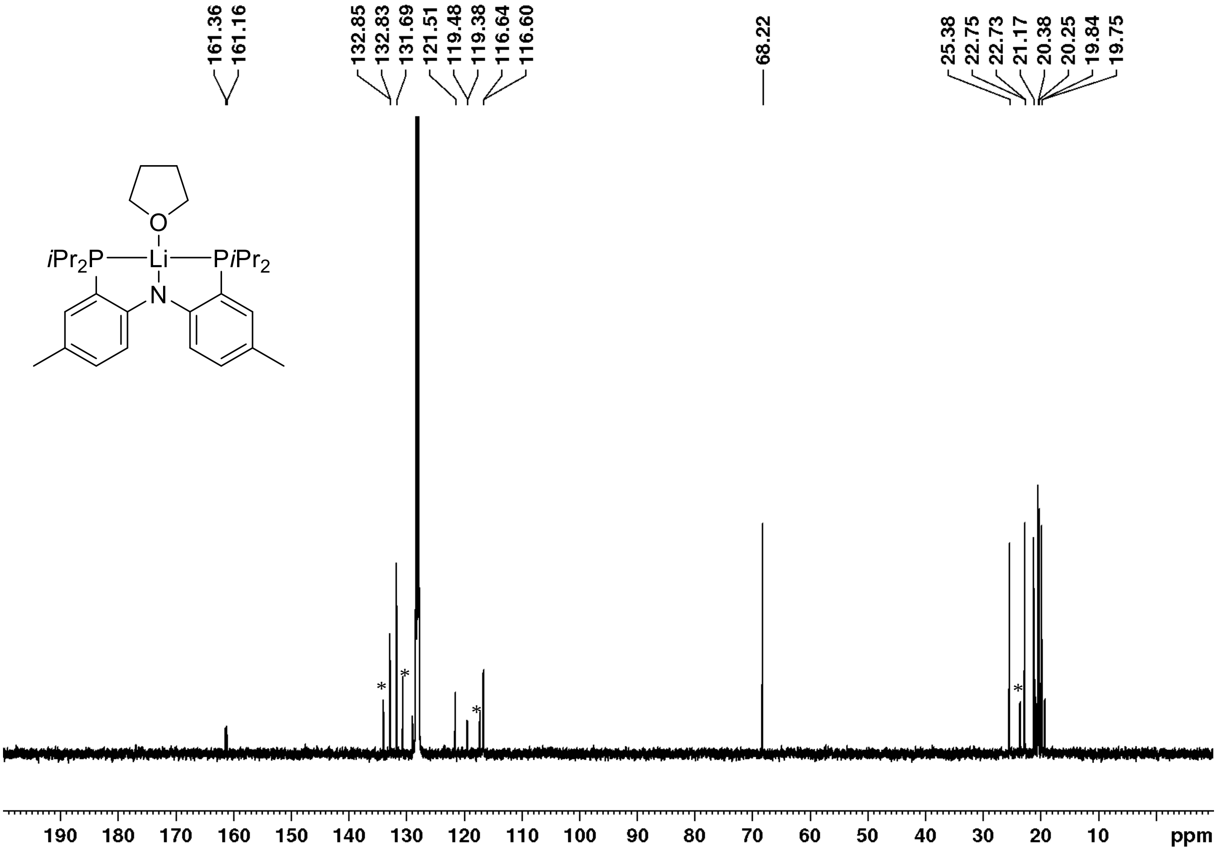

$^{13}C\{^{1}H\}$ NMR spectrum of **10** in C_6D_6 at 101 MHz. *^{iPr}**PNPH**

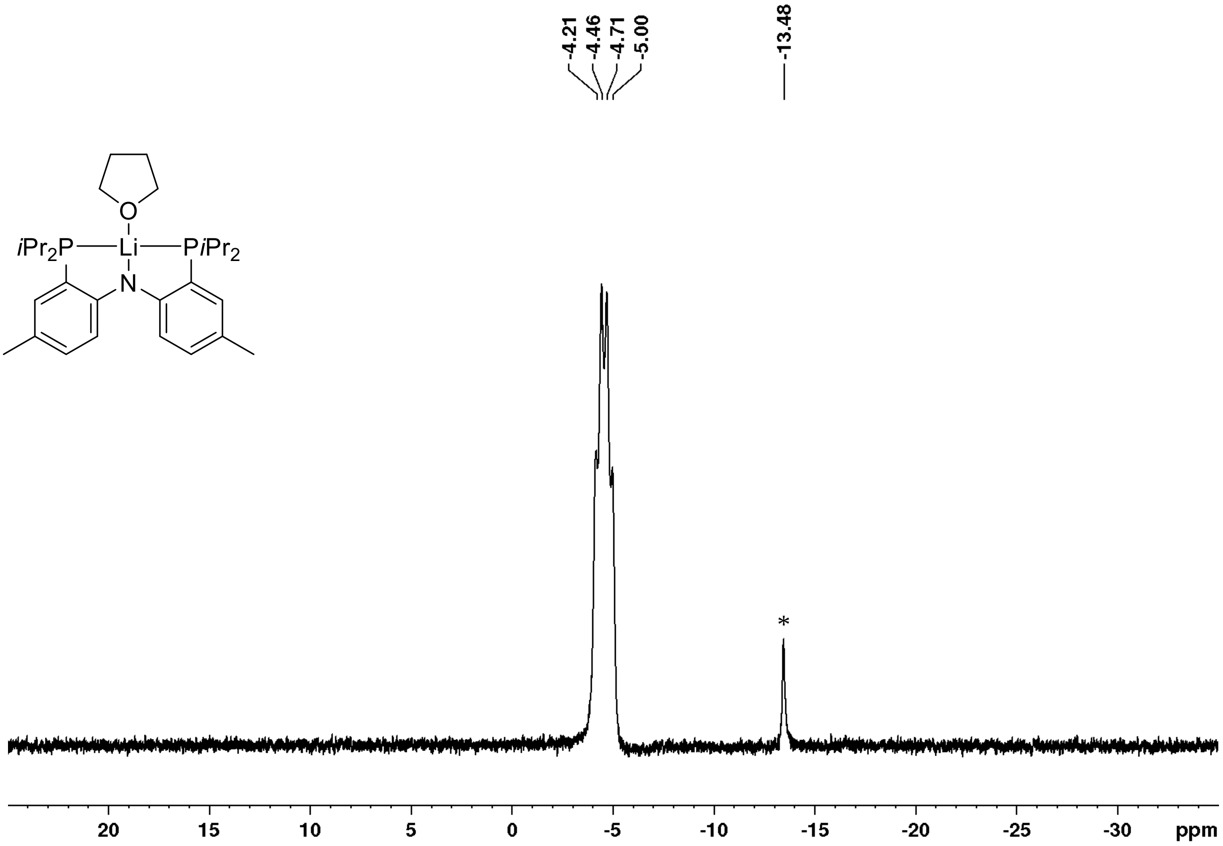

$^{31}P\{^1H\}$ NMR spectrum of **10** in C_6D_6 at 162 MHz. ****^{iPr}PNPH***

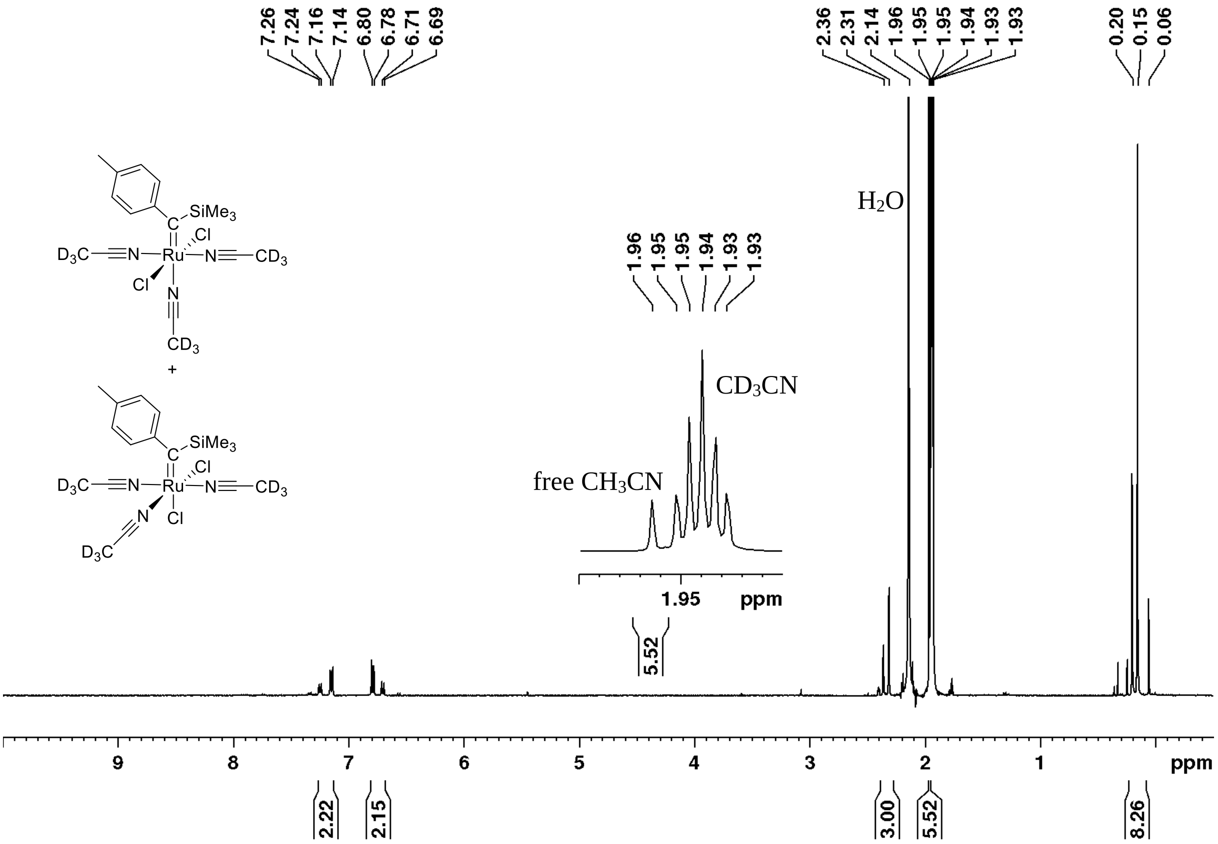

^{1}H NMR spectrum of **9** dissolved in CD_3CN at 400 MHz, showing a mixture of two isomeric complexes ligated to three CD_3CN ligands

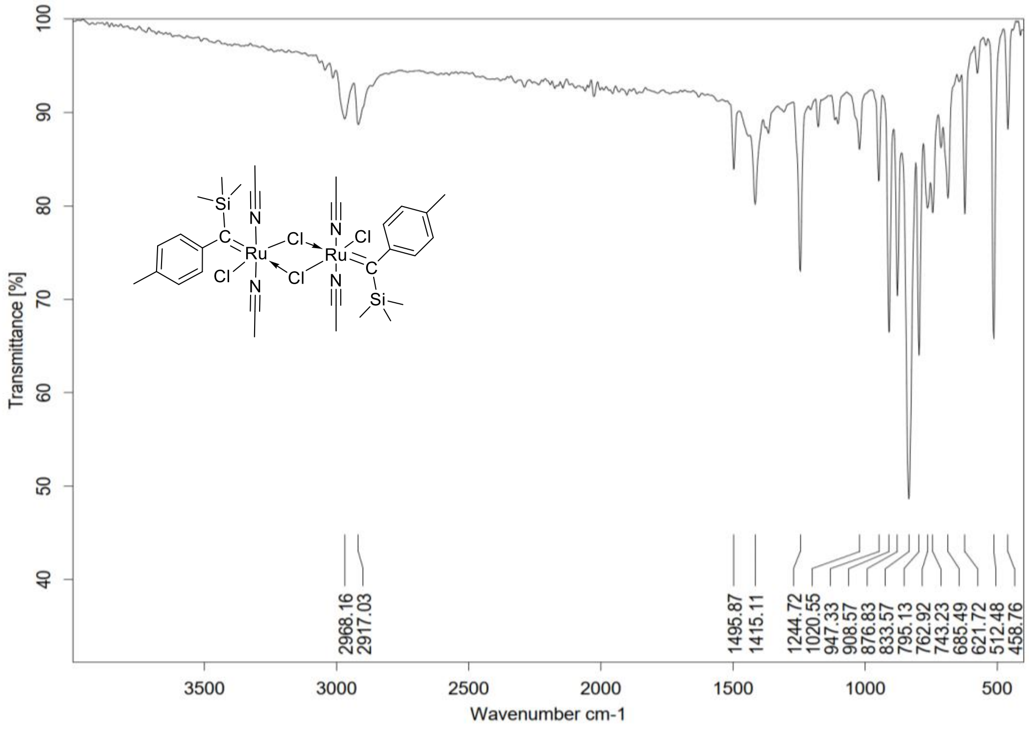

FT-IR spectrum of **9**

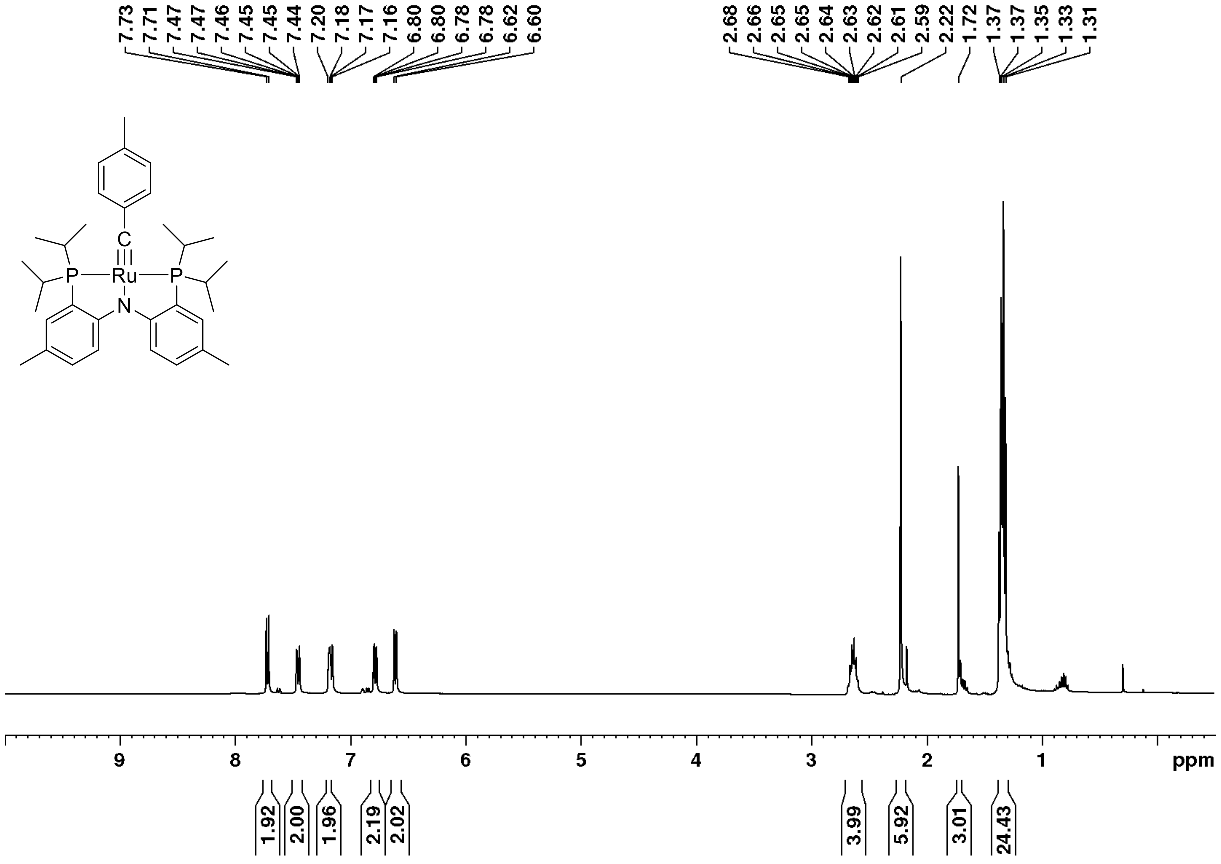

^{1}H NMR spectrum of ruthenium alkylidyne **12** in C_6D_6 at 400 MHz

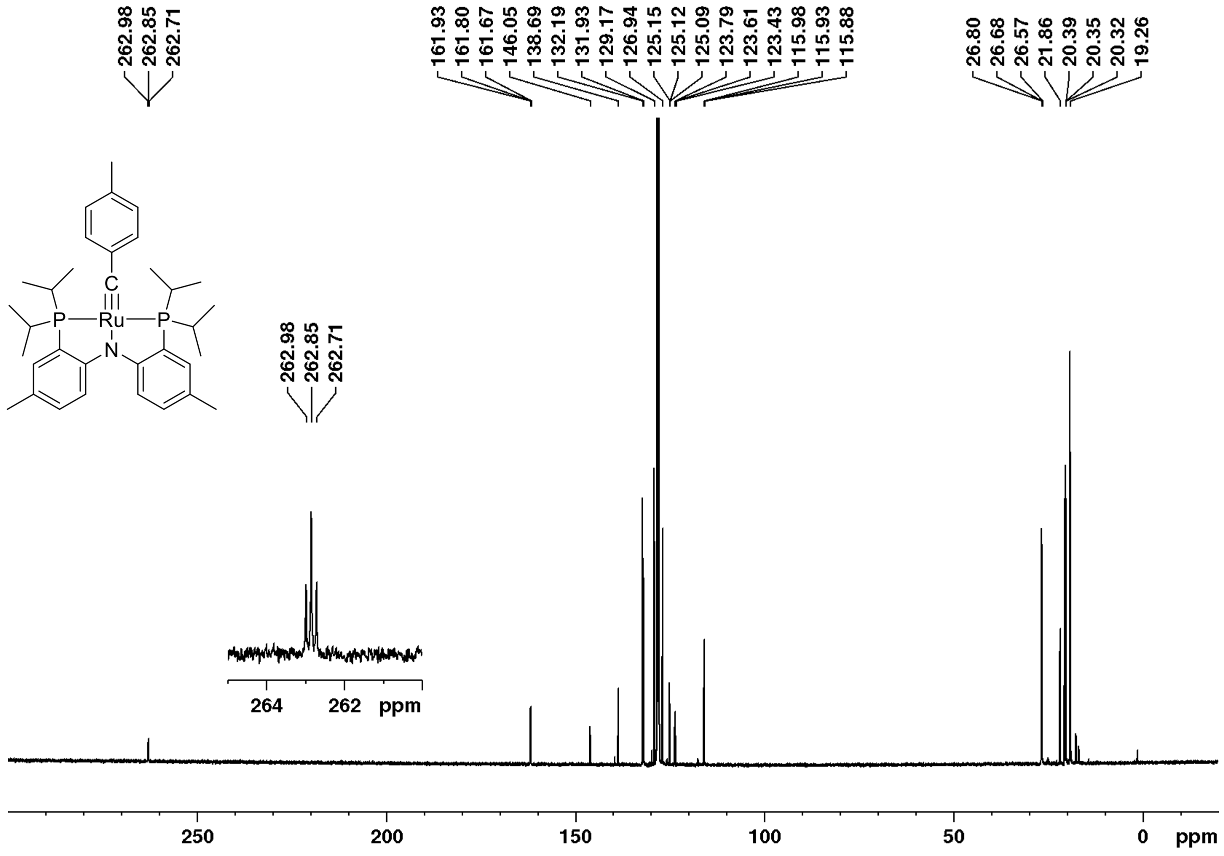

^{13}C{^{1}H} NMR spectrum of ruthenium alkylidyne **12** in C_6D_6 at 101 MHz

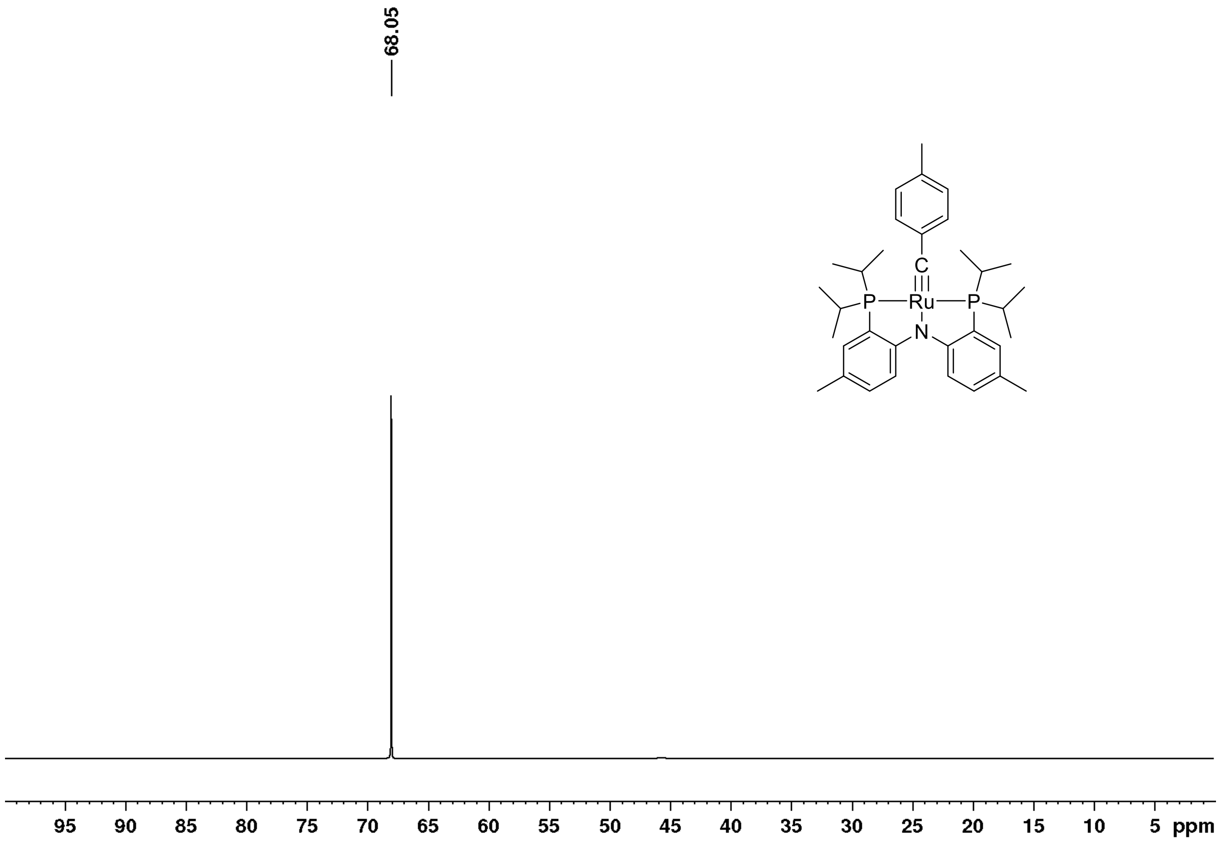

$^{31}P\{^{1}H\}$ NMR spectrum of ruthenium alkylidyne **12** in C_6D_6 at 162 MHz

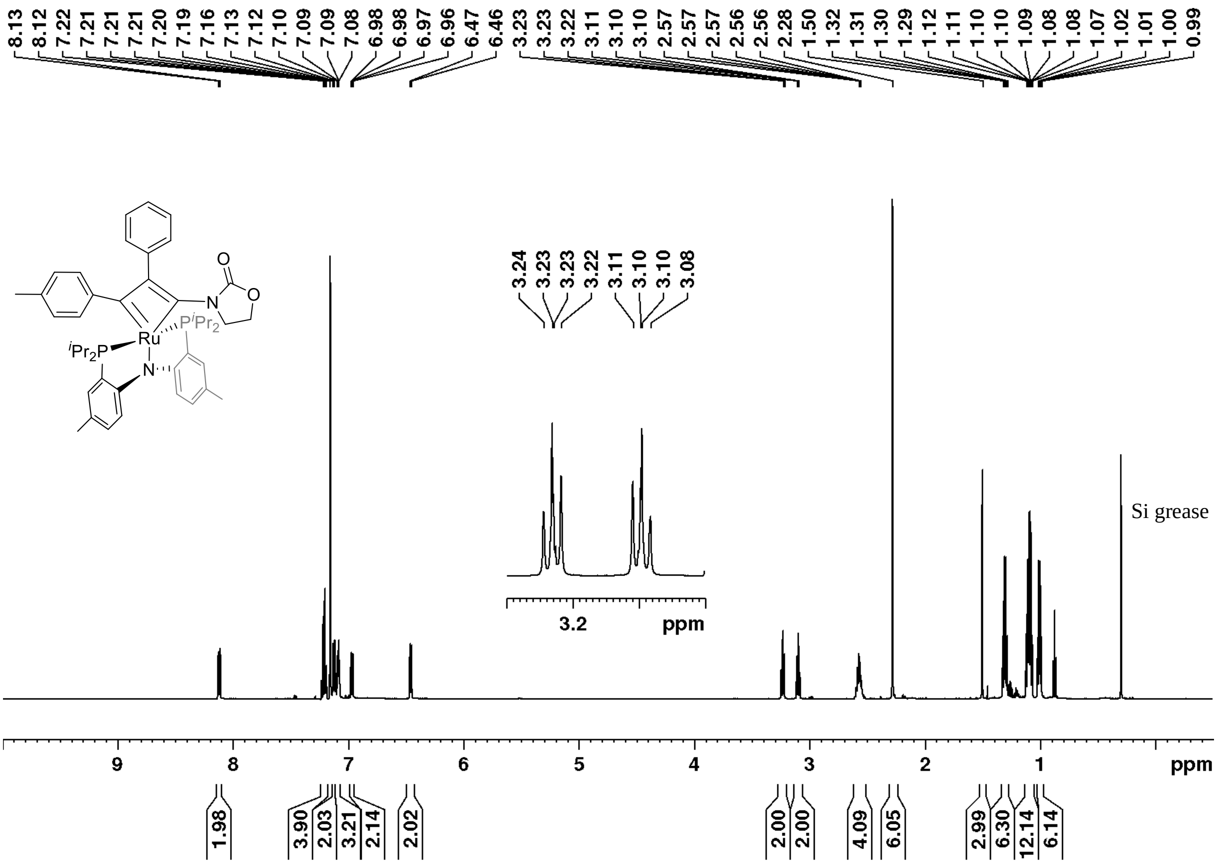

^{1}H NMR spectrum of metallacyclobutadiene **14** in C_6D_6 at 600 MHz

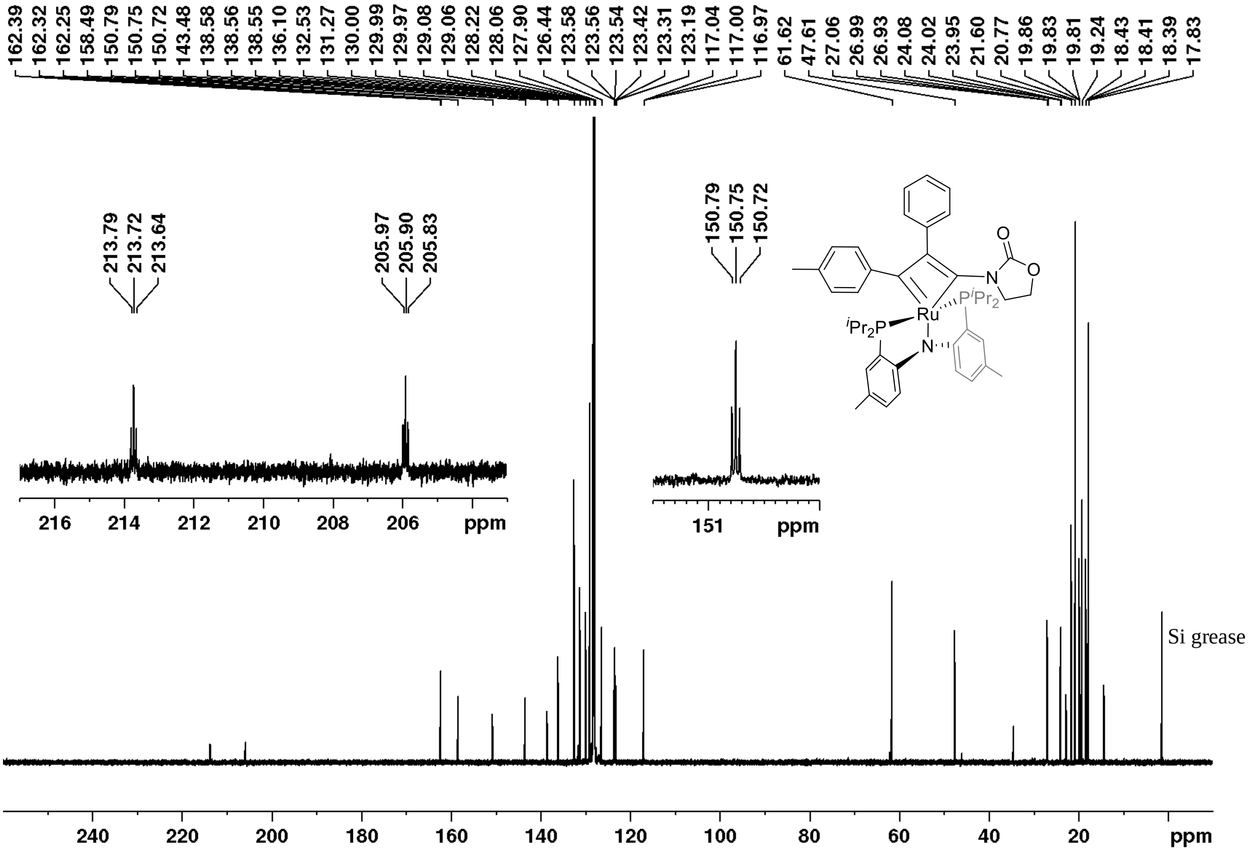

$^{13}C\{^{1}H\}$ NMR spectrum of metallacyclobutadiene **14** in C_6D_6 at 151 MHz

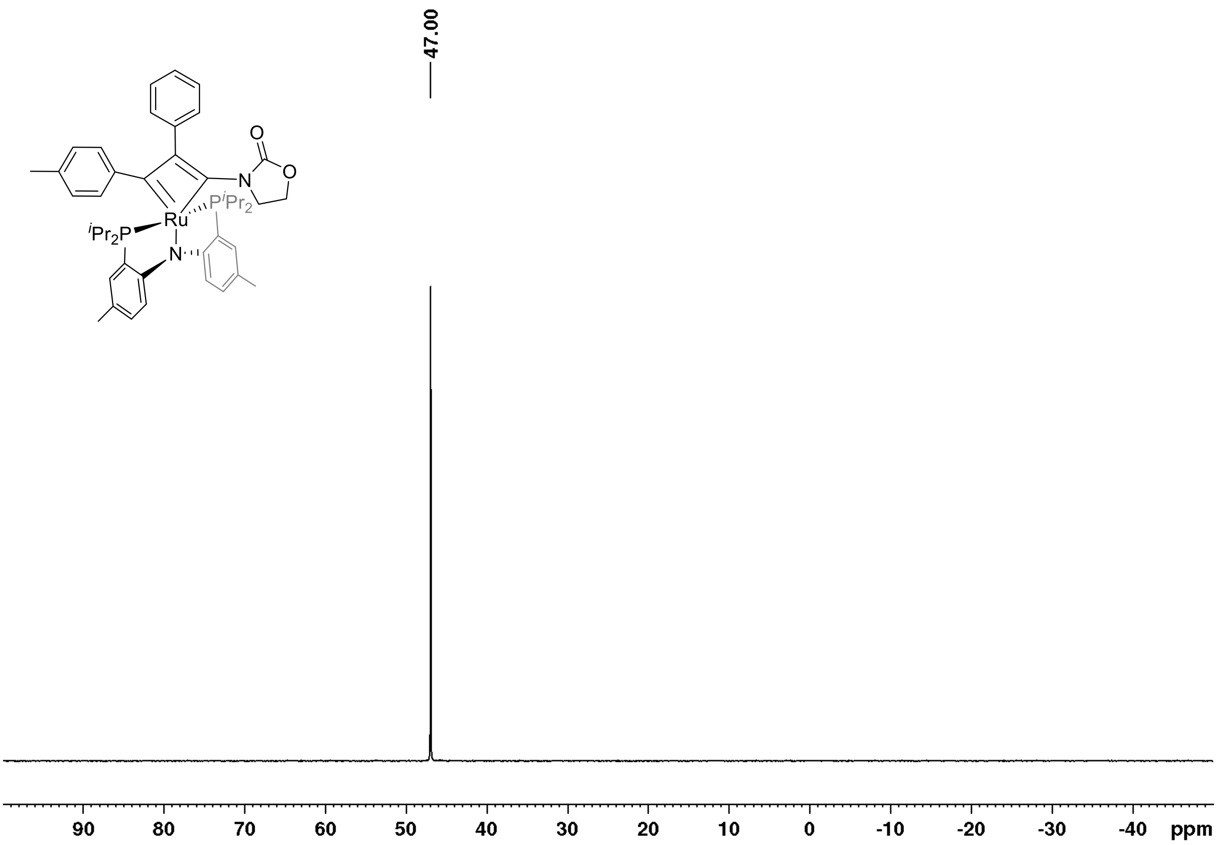

$^{31}P\{^{1}H\}$ NMR spectrum of metallacyclobutadiene **14** in C_6D_6 at 243 MHz

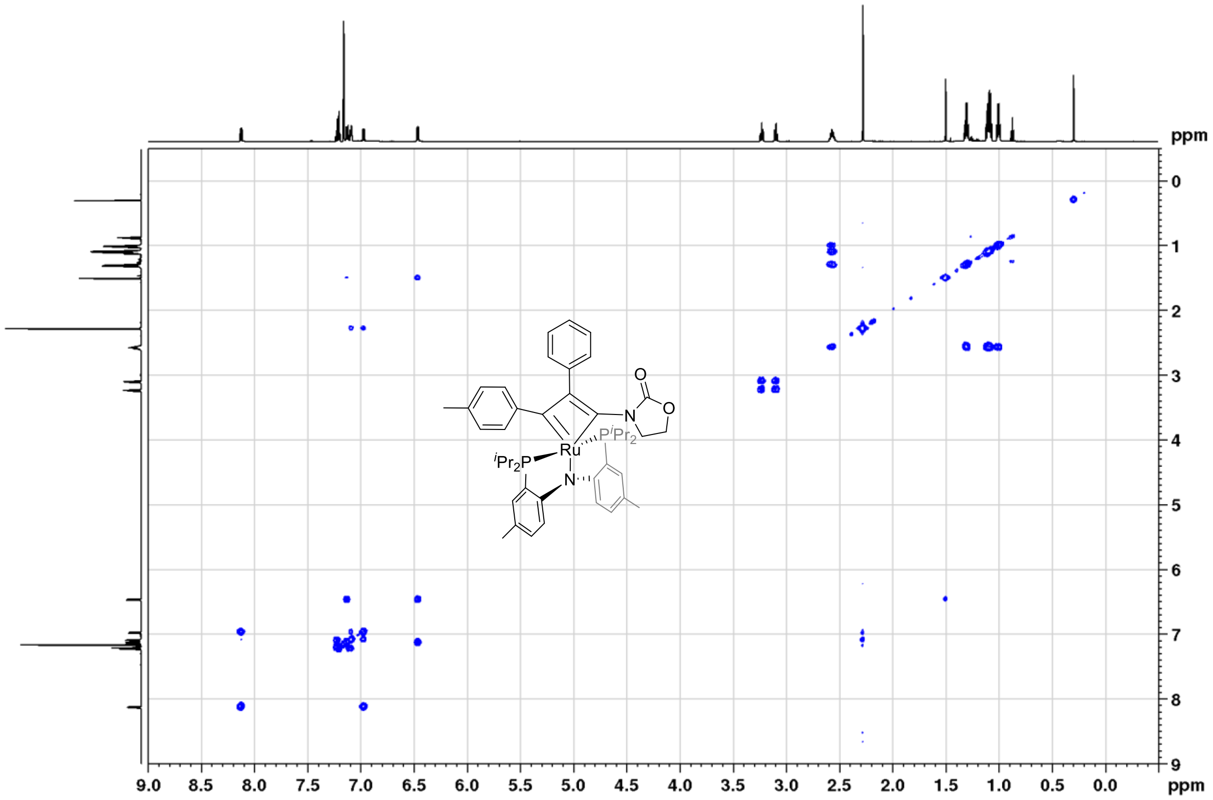

^{1}H-^{1}H COSY NMR spectrum of metallacyclobutadiene **14** in C_6D_6

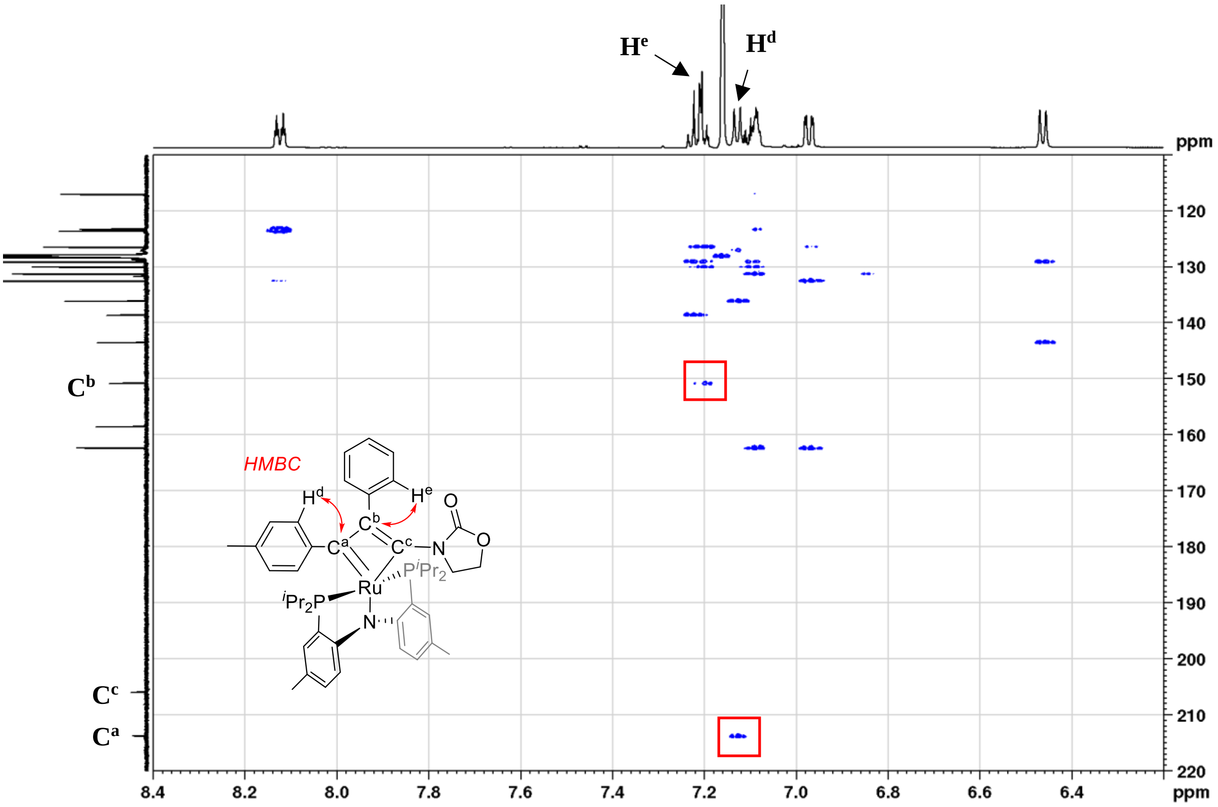

Partial ^{1}H-^{13}C HMBC NMR spectrum of metallacyclobutadiene **14** in C_6D_6

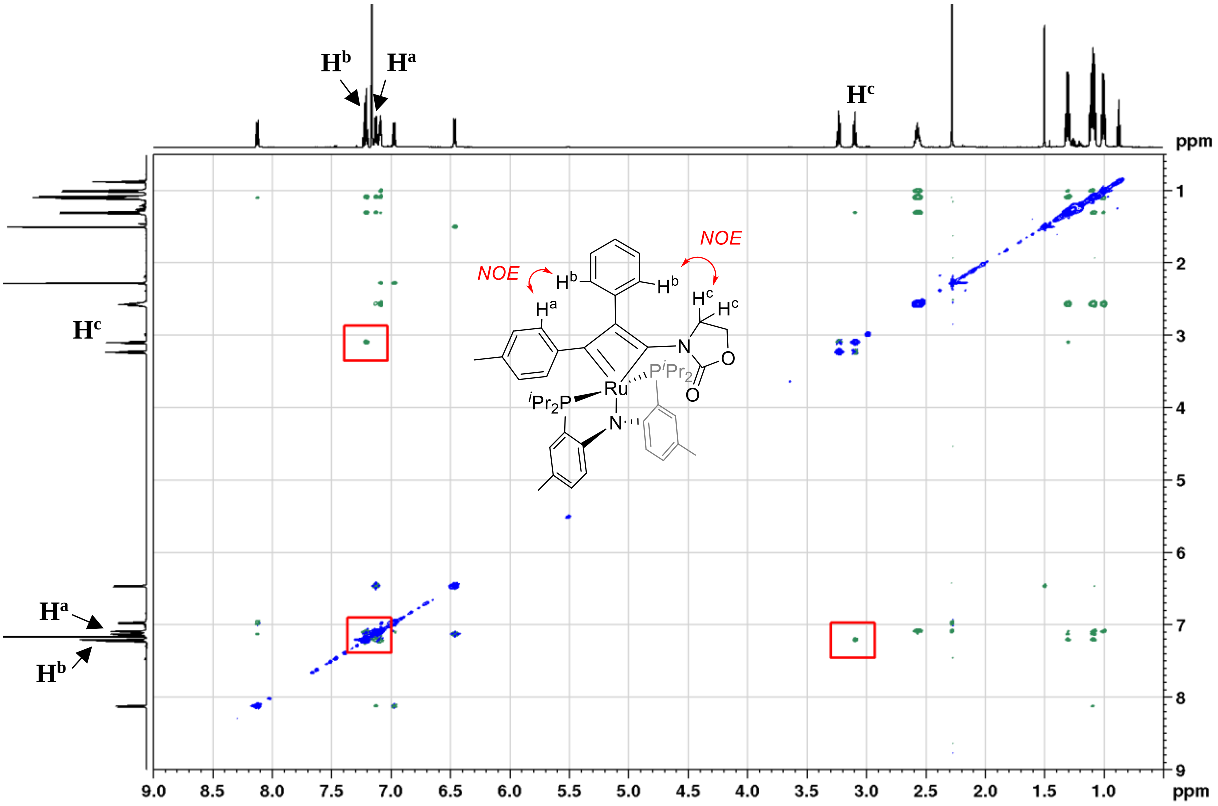

^{1}H-^{1}H NOESY NMR spectrum of metallacyclobutadiene **14** in C_6D_6

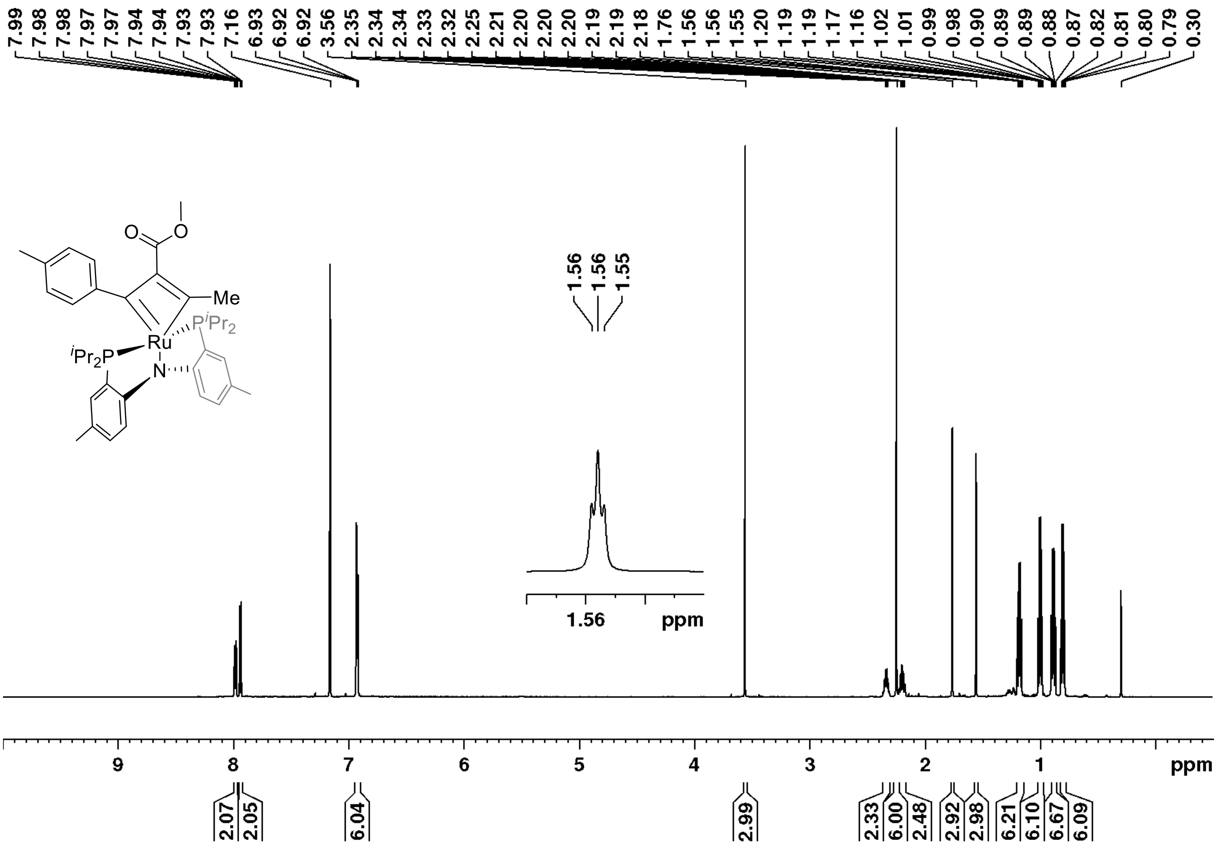

^{1}H NMR spectrum of metallacyclobutadiene **16** in C_6D_6 at 600 MHz

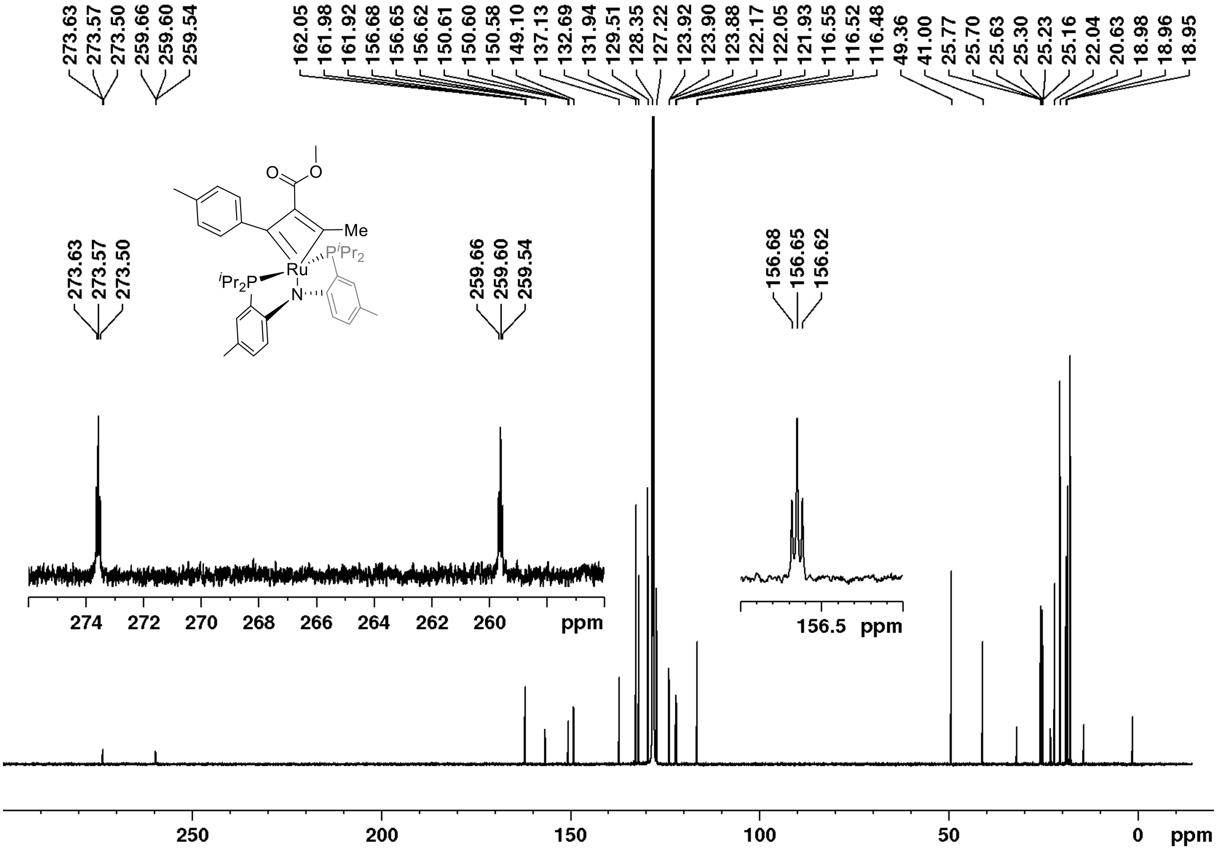

^{13}C{^{1}H} NMR spectrum of metallacyclobutadiene **16** in C_6D_6 at 151 MHz

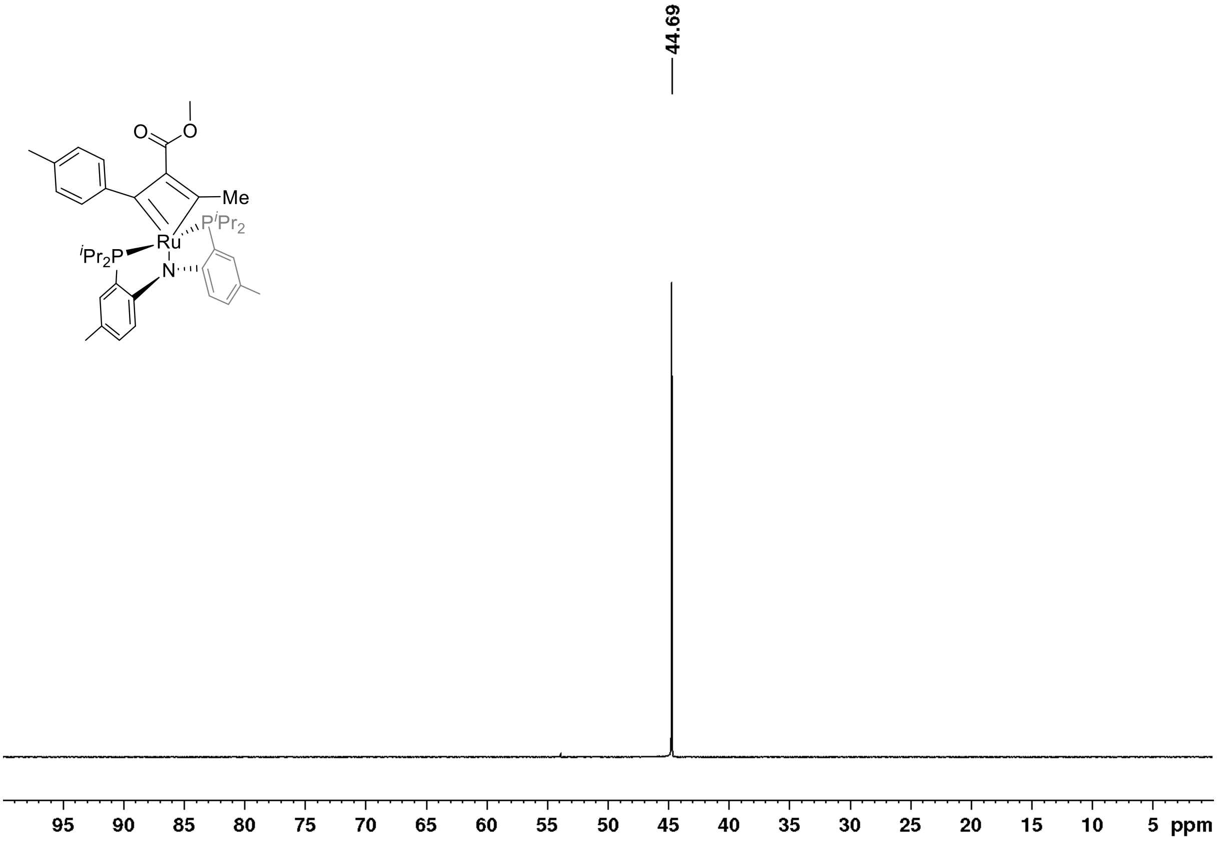

$^{31}P\{^{1}H\}$ NMR spectrum of metallacyclobutadiene **16** in C_6D_6 at 162 MHz

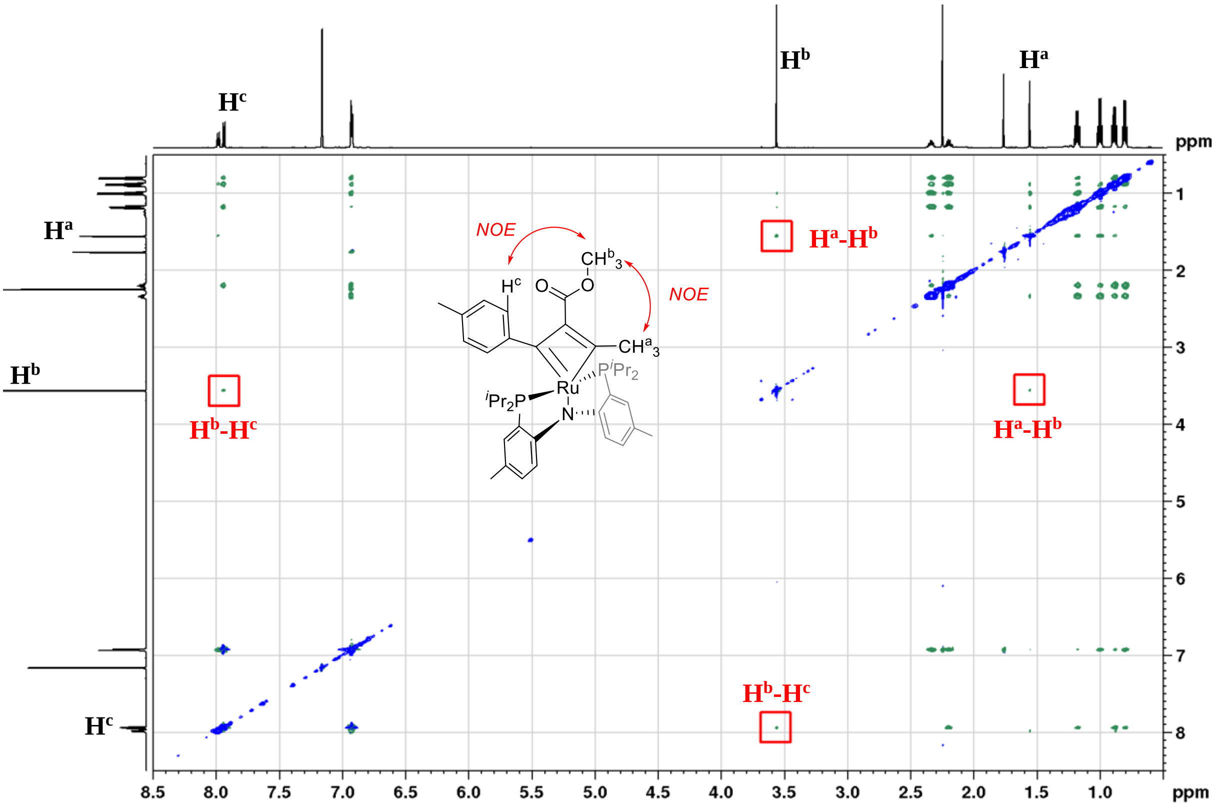

^{1}H-^{1}H NOESY NMR spectrum of metallacyclobutadiene **16**

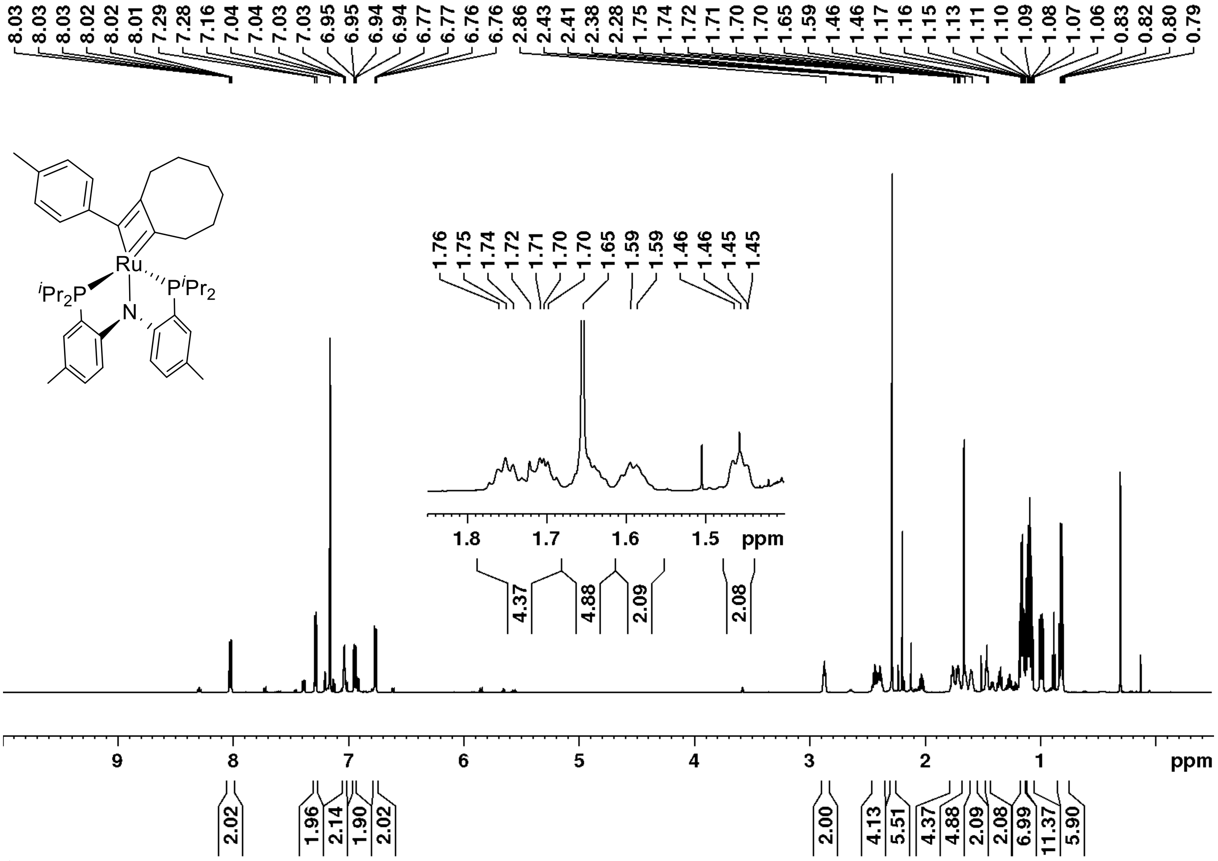

^1H NMR spectrum of metallacyclobutadiene **18** in C_6D_6 at 600 MHz at 298 K

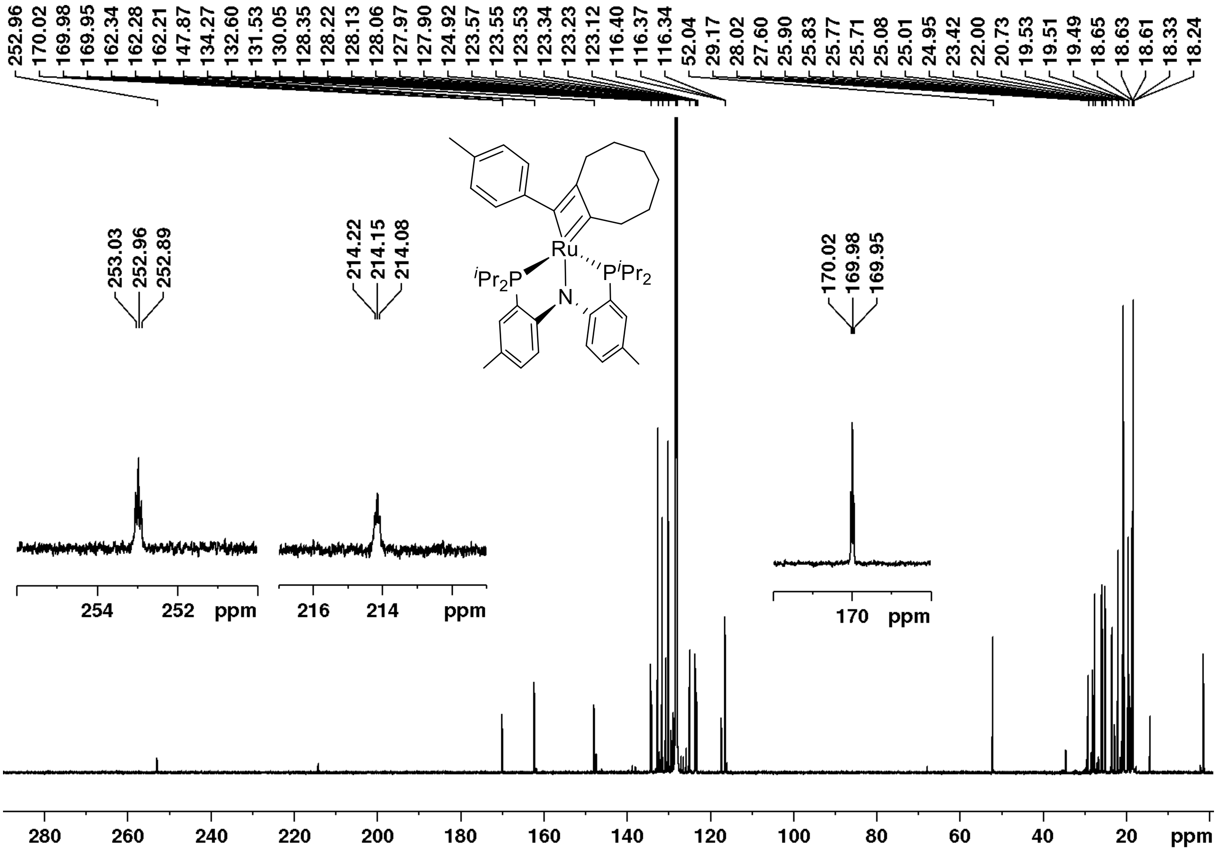

$^{13}C\{^1H\}$ NMR spectrum of metallacyclobutadiene **18** in C_6D_6 at at 151 MHz at 298 K

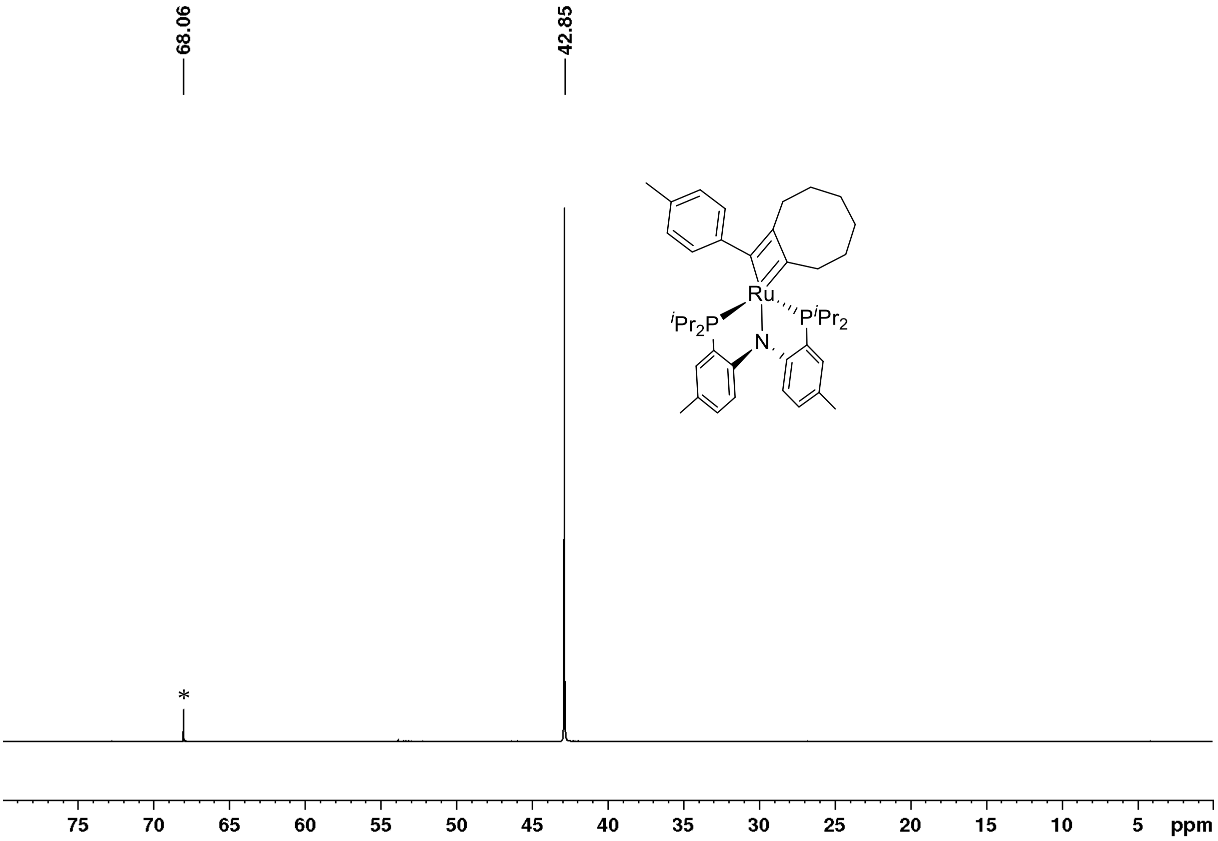

$^{31}P\{^1H\}$ NMR spectrum of metallacyclobutadiene **18** in C_6D_6 at 243 MHz at 298 K. * unreacted Ru alkylidyne **12**

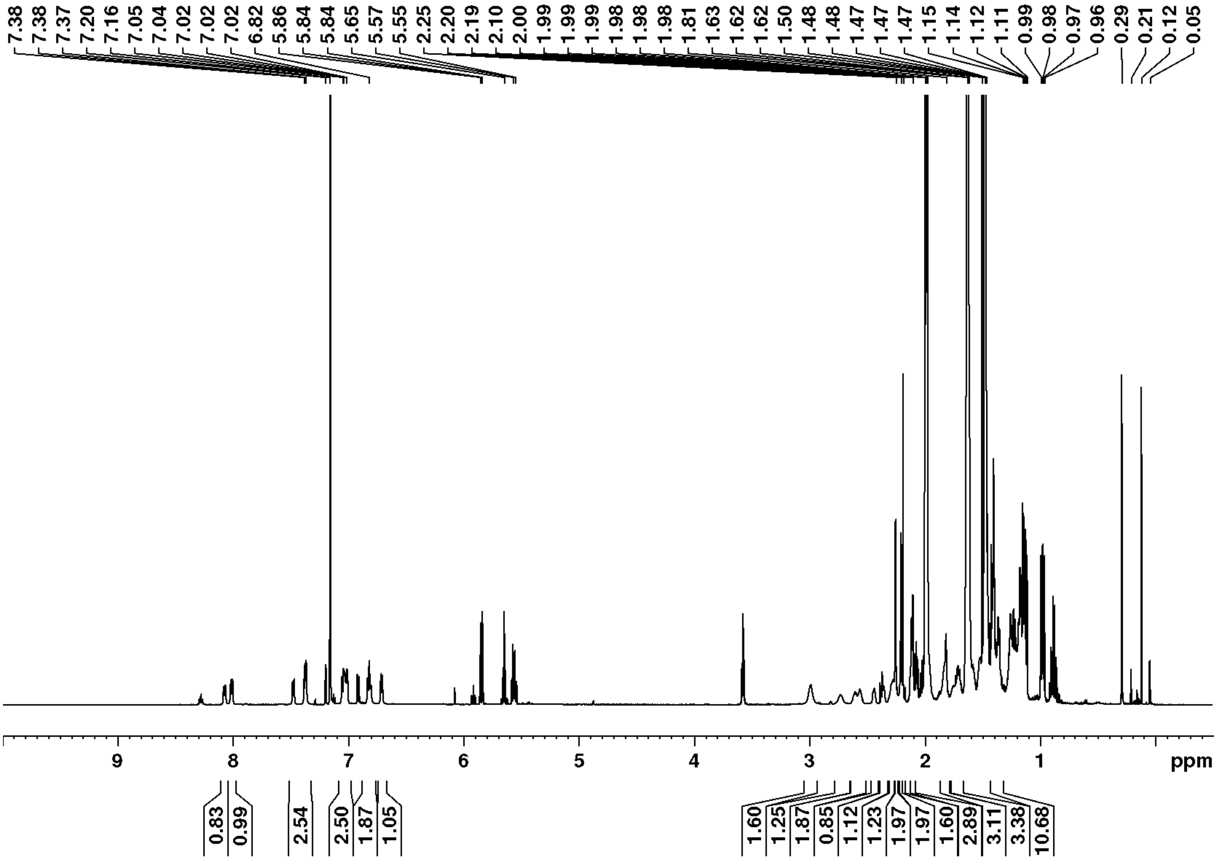

In situ ^{1}H NMR spectrum of the reaction of ruthenium alkylidyne **12** with excess cyclooctyne (10 equiv.) in C_6D_6 at 600 MHz at 298 K.

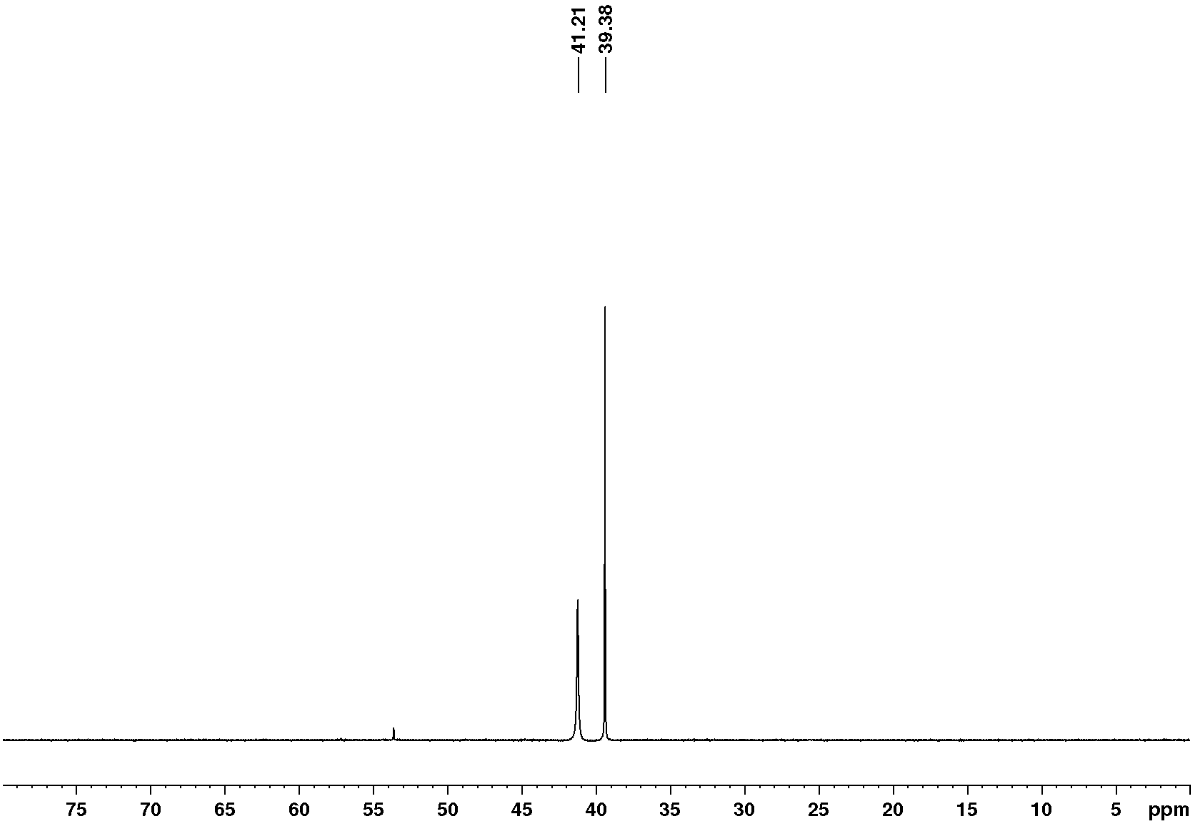

In situ ^{31}P{^{1}H} NMR spectrum of the reaction of ruthenium alkylidyne **12** with excess cyclooctyne (10 equiv.) in C_6D_6 at 243 MHz at 298 K.

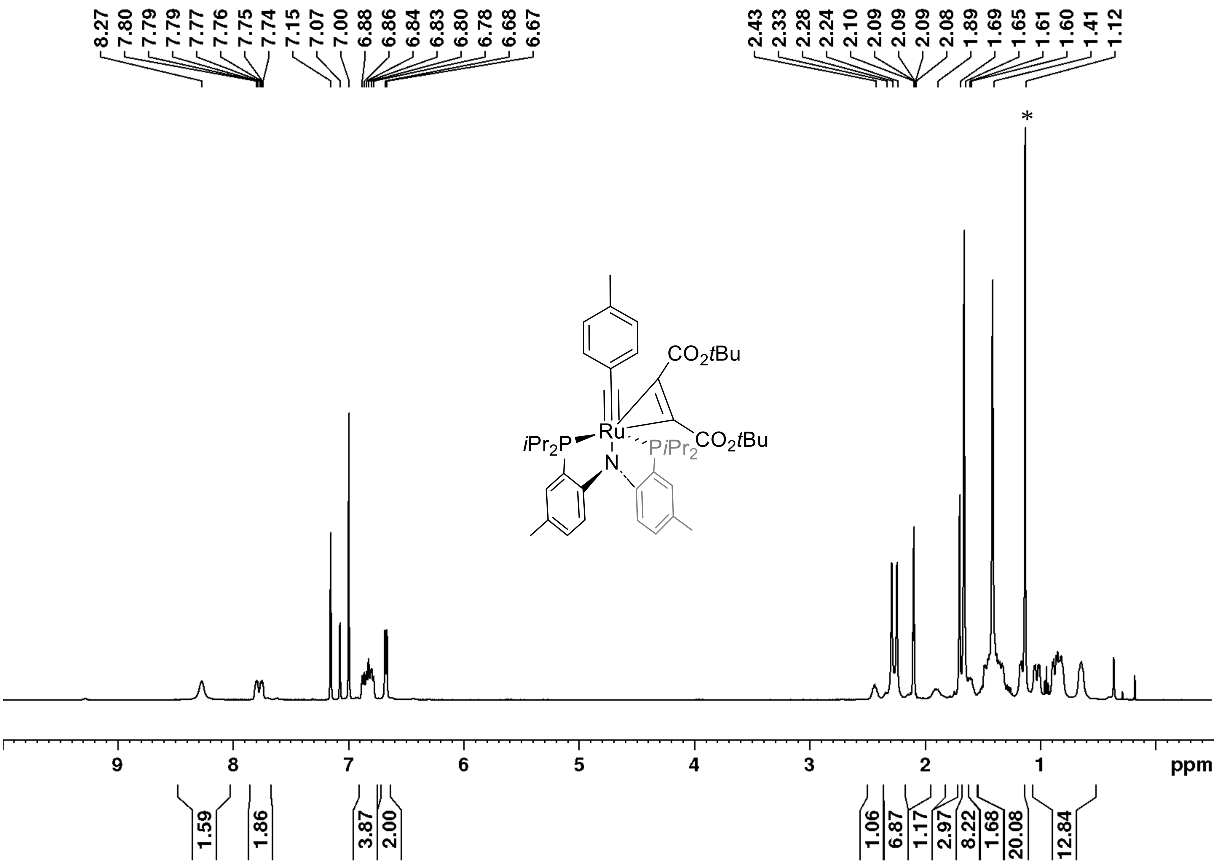

^{1}H NMR spectrum of ruthenium alkyne-alkylidyne complex **23** in [D8]-toluene at 500 MHz at 203 K. * free alkyne

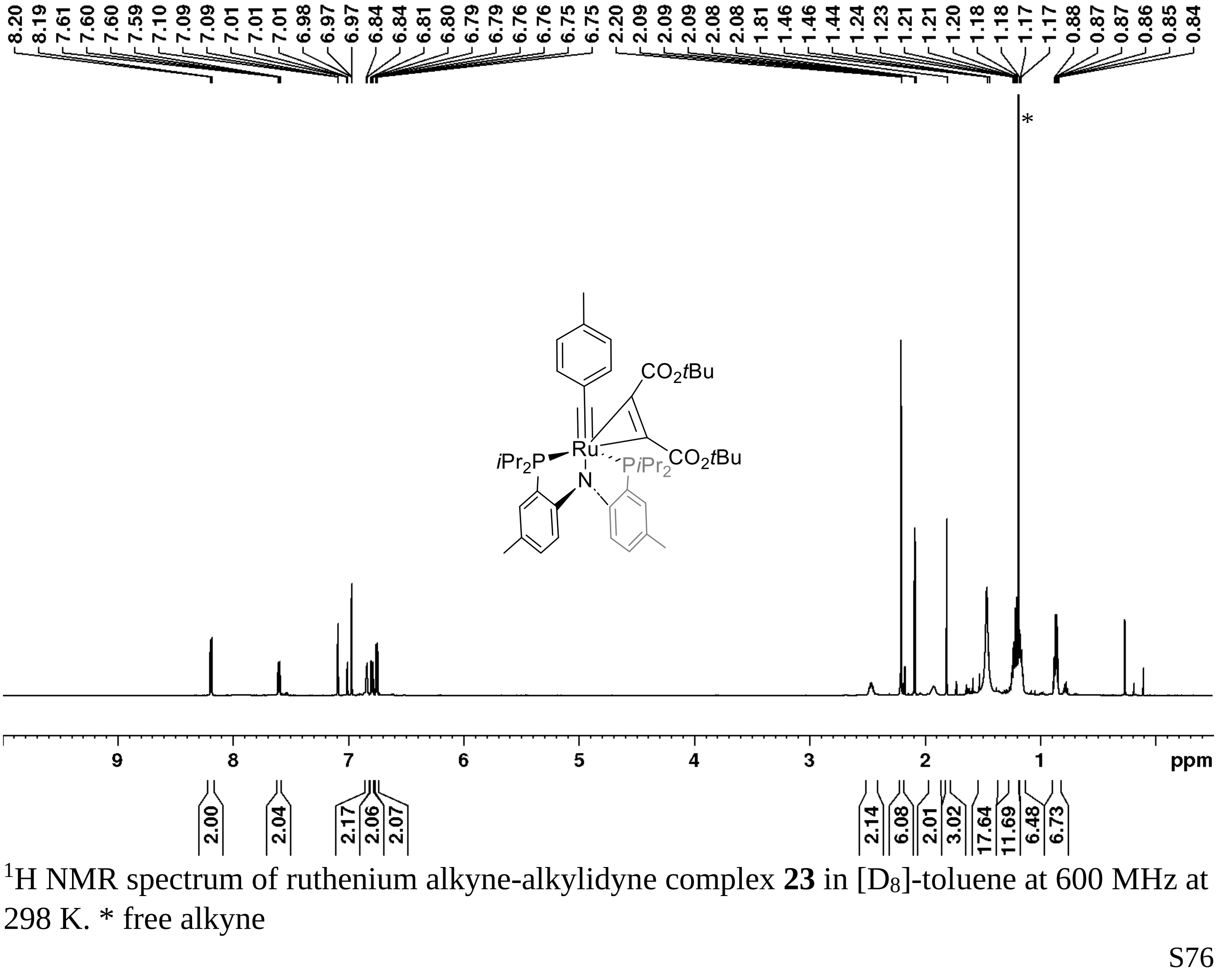

^{1}H NMR spectrum of ruthenium alkyne-alkylidyne complex **23** in [D8]-toluene at 600 MHz at 298 K. * free alkyne

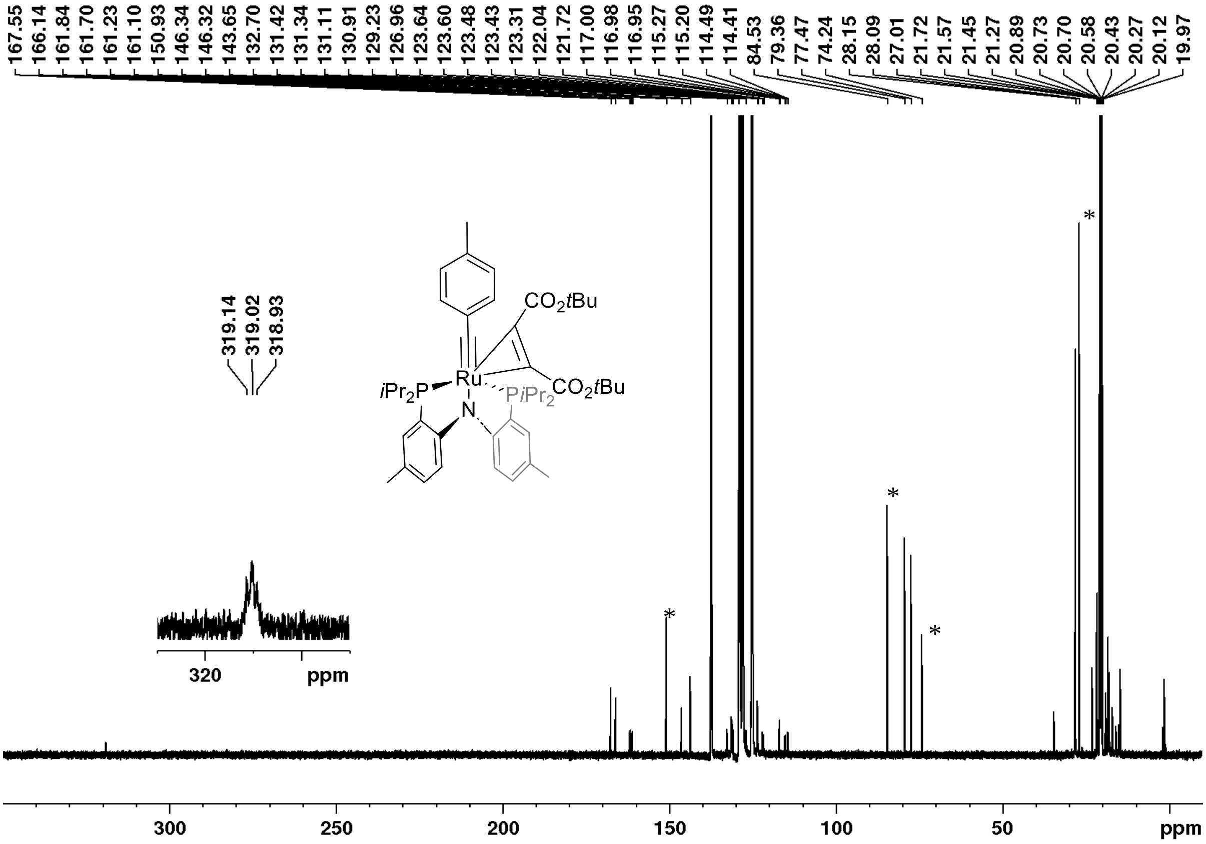

$^{13}C\{^{1}H\}$ NMR spectrum of ruthenium alkyne-alkylidyne complex **23** in [D8]-toluene at 126 MHz at 203 K. * free alkyne

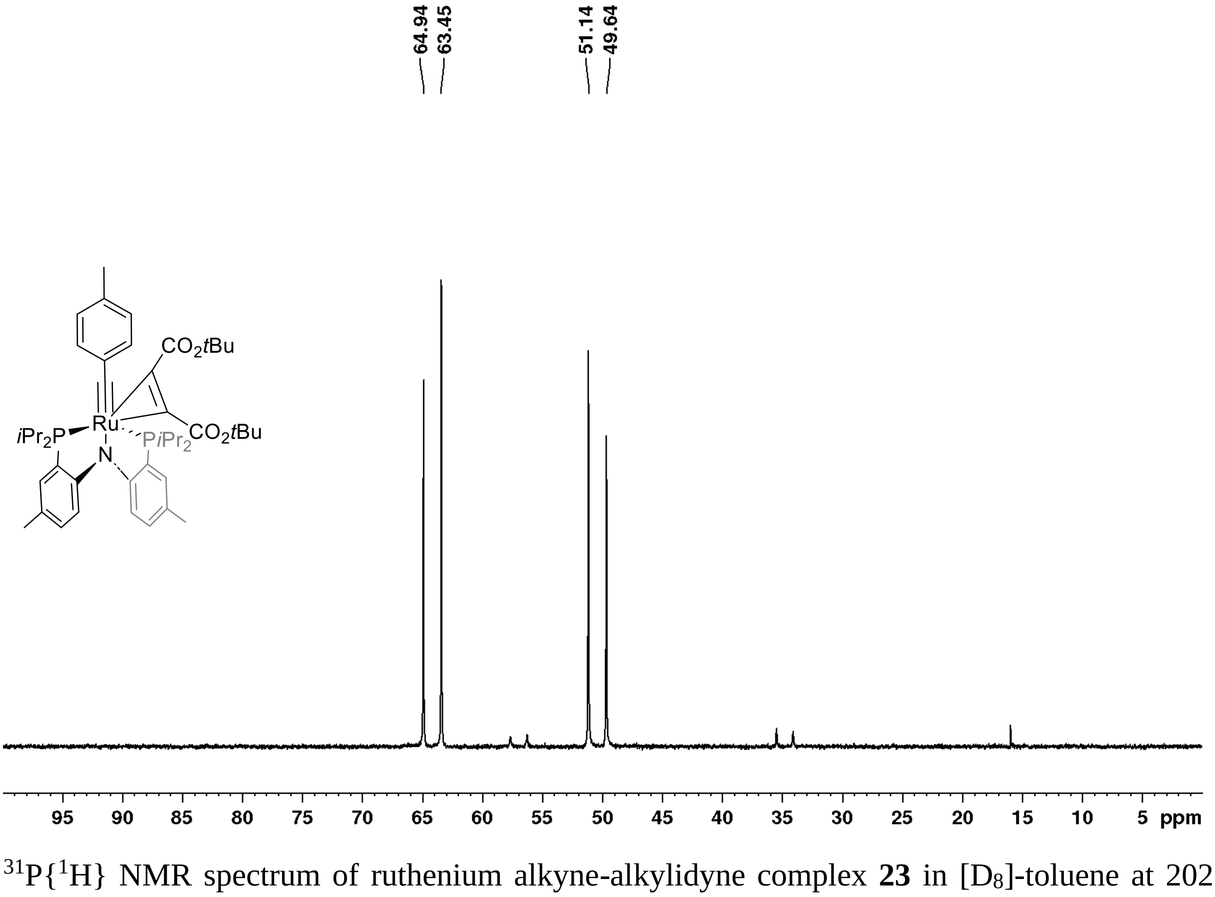

$^{31}P\{^{1}H\}$ NMR spectrum of ruthenium alkyne-alkylidyne complex **23** in [D8]-toluene at 202 MHz at 203 K

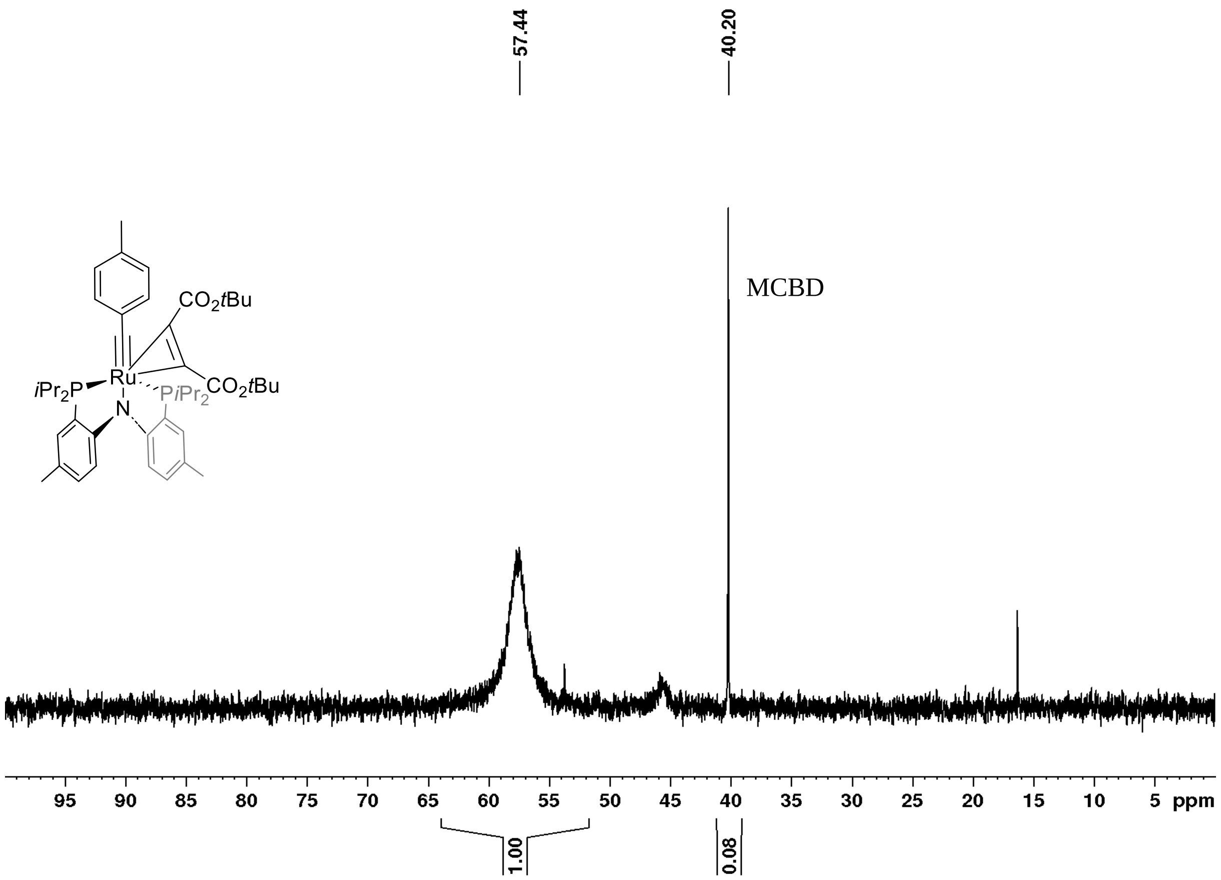

$^{31}P\{^1H\}$ NMR spectrum of ruthenium alkyne-alkylidyne complex **23** in [D$_8$]-toluene at 202 MHz at 298 K.

References

(1) A. M. Borys, *Organometallics* **2023**, *42*, 182–196.

(2) B. Peng, X. Huang, L.-G. Xie, N. Maulide, *Angew. Chem. Int. Ed.* **2014**, *53*, 8718–8721.

(3) L. Brandsma, H. D. Verkruijsse, *Synthesis* **1978**, *290*.

(4) G. R. Fulmer, A. J. M. Miller, N. H. Sherden, H. E. Gottlieb, A. Nudelman, B. M. Stoltz, J. E. Bercaw, K. I. Goldberg, *Organometallics* **2010**, *29*, 2176–2179.

(5) A. G. Brook, P. F. Jones, *Can. J. Chem.* **1969**, *47*, 4353–4358.

(6) S. Han, S. R. Kass, *J. Chem. Soc., Perkin Trans. 1* **1999**, 1553–1558.

(7) a) K. Yamamoto, S. Suzuki, J. Tsuji, *Tetrahedron Lett.* **1980**, *21*, 1653–1656; b) K. Yamamoto, A. Hayashi, S. Suzuki, J. Tsuji, *Organometallics* **1987**, *6*, 974–979.

(8) W. Weng, L. Yang, B. M. Foxman, O. V. Ozerov, *Organometallics* **2004**, *23*, 4700–4705.

(9) For alkyne-ruthenium complexes in which the signals of the bound triple bond and that of the free ligand have very similar ^{13}C NMR shifts, see: M. A. Bennett, G. A. Heath, D. C. R. Hockless, I. Kovacik, A. C. Willis, *Organometallics* **1998**, *17*, 5867–5873.

(10) a) F. Neese, *WIREs Comput. Mol. Sci.* **2012**, *2*, 73–78; b) F. Neese, F. Wennmohs, U. Becker, C. Riplinger, *J. Chem. Phys.* **2020**, *152*, 224108; c) F. Neese, *WIREs Comput. Mol. Sci.* **2025**, *15*, e70019.

(11) a) A. D. Becke, *J. Chem. Phys.* **1993**, *98*, 1372–1377; b) F. Weigend, *Phys. Chem. Chem. Phys.* **2006**, *8*, 1057–1065; c) R. Izsák, F. Neese, *J. Chem. Phys.* **2011**, *135*, 144105. d) F. Weigend, R. Ahlrichs, *Phys. Chem. Chem. Phys.* **2005**, *7*, 3297–3305; e) C. Lee, W. Yang, R. G. Parr, *Phys. Rev. B* **1988**, *37*, 785–789; f) M. Garcia-Ratés, F. Neese, *J. Comput. Chem.* **2019**, *40*, 1816–1828; g) M. Garcia-Ratés, F. Neese, *J. Comput. Chem.* **2020**, *41*, 922–939; h) D. Andrae, U. Häußermann, M. Dolg, H. Stoll, H. Preuß, *Theor. Chim. Acta* **1990**, *77*, 123–141; i) E. Caldeweyher, C. Bannwarth, S. Grimme, *J. Chem. Phys.* **2017**, *147*, 034112.

(12) S. Grimme, *Chem. Eur. J.* **2012**, *18*, 9955–9964.
